# Scientific novelty beyond the experiment

**DOI:** 10.1111/1751-7915.14222

**Published:** 2023-02-14

**Authors:** John E. Hallsworth, Zulema Udaondo, Carlos Pedrós‐Alió, Juan Höfer, Kathleen C. Benison, Karen G. Lloyd, Radamés J. B. Cordero, Claudia B. L. de Campos, Michail M. Yakimov, Ricardo Amils

**Affiliations:** ^1^ Institute for Global Food Security, School of Biological Sciences Queen's University Belfast Belfast UK; ^2^ Department of Biomedical Informatics University of Arkansas for Medical Sciences Little Rock Arkansas USA; ^3^ Department of Systems Biology Centro Nacional de Biotecnología (CSIC) Madrid Spain; ^4^ Escuela de Ciencias del Mar Pontificia Universidad Católica de Valparaíso Valparaíso Chile; ^5^ Department of Geology and Geography West Virginia University Morgantown West Virginia USA; ^6^ Microbiology Department University of Tennessee Knoxville Tennessee USA; ^7^ Department of Molecular Microbiology and Immunology Johns Hopkins Bloomberg School of Public Health Baltimore Maryland USA; ^8^ Institute of Science and Technology Universidade Federal de Sao Paulo (UNIFESP) São José dos Campos SP Brazil; ^9^ Institute of Polar Sciences, ISP‐CNR Messina Italy; ^10^ Department of Molecular Biology, Centro de Biología Molecular Severo Ochoa (CSIC‐UAM) Nicolás Cabrera n° 1, Universidad Autónoma de Madrid Madrid Spain; ^11^ Department of Planetology and Habitability Centro de Astrobiología (INTA‐CSIC) Torrejón de Ardoz Spain

## Abstract

Practical experiments drive important scientific discoveries in biology, but theory‐based research studies also contribute novel—sometimes paradigm‐changing—findings. Here, we appraise the roles of theory‐based approaches focusing on the experiment‐dominated wet‐biology research areas of microbial growth and survival, cell physiology, host–pathogen interactions, and competitive or symbiotic interactions. Additional examples relate to analyses of genome‐sequence data, climate change and planetary health, habitability, and astrobiology. We assess the importance of thought at each step of the research process; the roles of natural philosophy, and inconsistencies in logic and language, as drivers of scientific progress; the value of thought experiments; the use and limitations of artificial intelligence technologies, including their potential for interdisciplinary and transdisciplinary research; and other instances when theory is the most‐direct and most‐scientifically robust route to scientific novelty including the development of techniques for practical experimentation or fieldwork. We highlight the intrinsic need for human engagement in scientific innovation, an issue pertinent to the ongoing controversy over papers authored using/authored by artificial intelligence (such as the large language model/chatbot ChatGPT). Other issues discussed are the way in which aspects of language can bias thinking towards the spatial rather than the temporal (and how this biased thinking can lead to skewed scientific terminology); receptivity to research that is non‐mainstream; and the importance of theory‐based science in education and epistemology. Whereas we briefly highlight classic works (those by Oakes Ames, Francis H.C. Crick and James D. Watson, Charles R. Darwin, Albert Einstein, James E. Lovelock, Lynn Margulis, Gilbert Ryle, Erwin R.J.A. Schrödinger, Alan M. Turing, and others), the focus is on microbiology studies that are more‐recent, discussing these in the context of the scientific process and the types of scientific novelty that they represent. These include several studies carried out during the 2020 to 2022 lockdowns of the COVID‐19 pandemic when access to research laboratories was disallowed (or limited). We interviewed the authors of some of the featured microbiology‐related papers and—although we ourselves are involved in laboratory experiments and practical fieldwork—also drew from our own research experiences showing that such studies can not only produce new scientific findings but can also transcend barriers between disciplines, act counter to scientific reductionism, integrate biological data across different timescales and levels of complexity, and circumvent constraints imposed by practical techniques. In relation to urgent research needs, we believe that climate change and other global challenges may require approaches beyond the experiment.

## Graphical abstract

Practical experiments drive important scientific discoveries in biology, but theory‐based research studies also contribute novel—sometimes paradigm‐changing—findings. Here, we appraise the roles of theory‐based approaches focusing on the experiment‐dominated wet‐biology research areas of microbiology. Theory‐based studies can transcend barriers imposed by differences between disciplines, act counter to scientific reductionism, integrate biological data across different timescales and levels of complexity, and circumvent constraints imposed by practical techniques. In relation to urgent research needs, we believe that climate change and other global challenges necessitate approaches beyond the experiment.

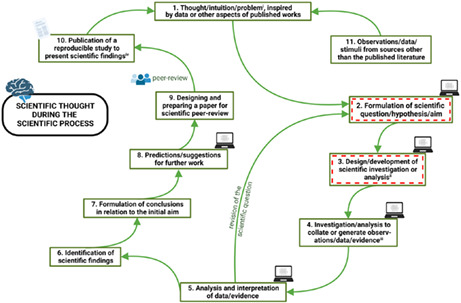

## INTRODUCTION

Microbiology is primarily an experiment‐led scientific discipline, and lucid and innovative thinking plays a key role in this practical experimentation. Whereas most researchers are involved in laboratory‐ or field‐based experiments, some research fields require approaches based on thought or theory alone. These can include modelling (e.g., Aldridge et al., 2006; Poliseli et al., 2022), in‐silico studies of molecular dynamics (Crippa et al., 2022), and the meta‐analyses of publicly available bioinformatics databases (Cremin et al., 2022; Prakash & Taylor, 2012). Other fields where theory‐based approaches are used, albeit that practical experiments are more‐commonly used, include host–pathogen interactions (Foster et al., 2017), cell physiology and stress phenotypes (Brown, 1990), symbiotic interactions (Graf et al., 2021), growth and survival (Madigan et al., 2021), and competitive interactions between microbes (Cray et al., 2016).

**FIGURE 1:**
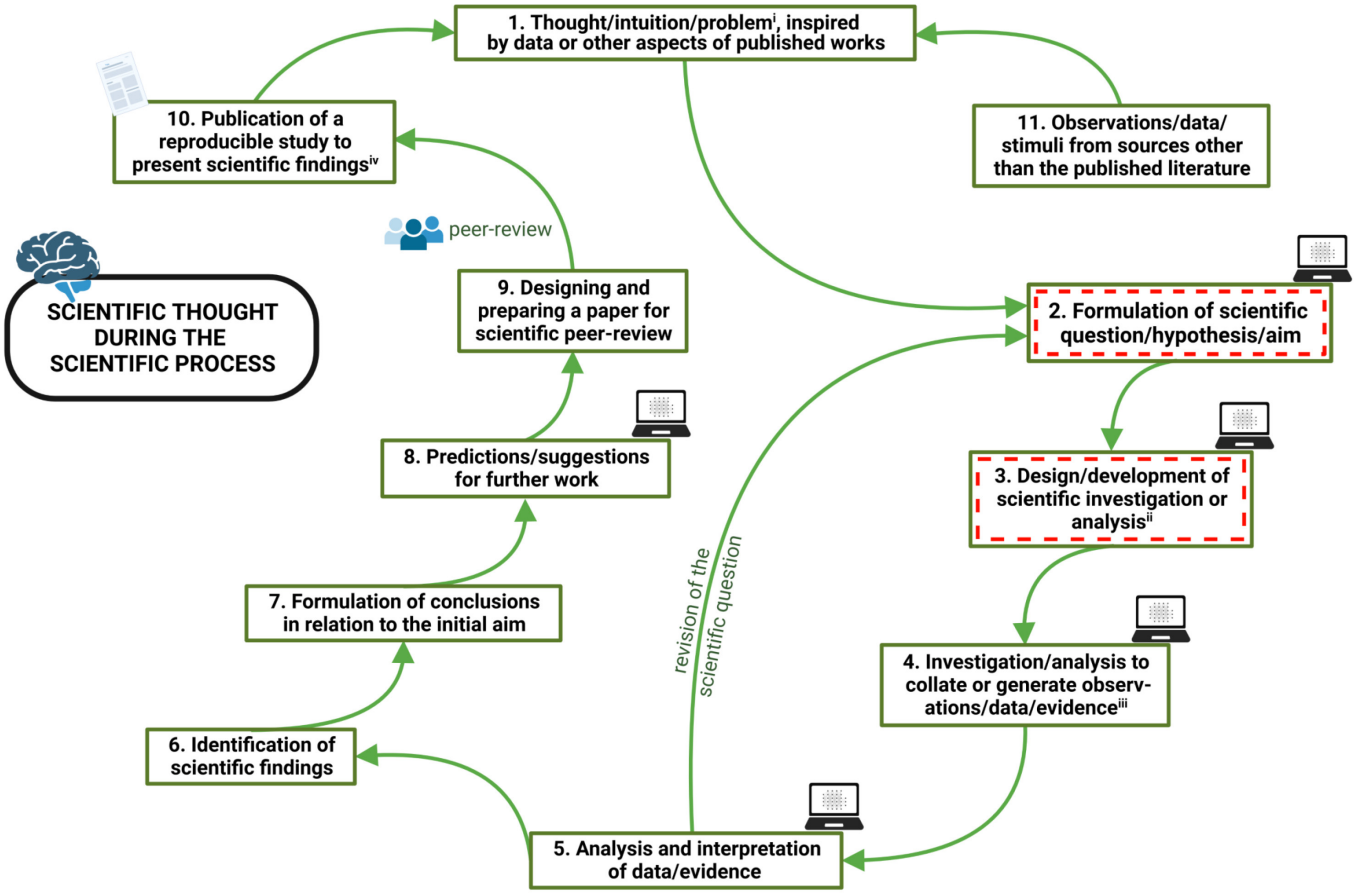
Basic components and tenets of the scientific process, showing that scientific thought is needed throughout (green), and where practical experiments can also be required (red dashes). Stages where artificial intelligence may be most‐useful are indicated by laptop icons. Chance and serendipity can also play important roles in the scientific process, potentially at every stage (see main text). ^i^ Commonly the starting point; this might also involve the identification of a gap in current knowledge. ^ii^ With methodological development if needed. ^iii^ Typically repeated to independently validate the results. ^iv^ These findings could take the form of data, a model, a new hypothesis, etc. We believe that, in theory at least, artificial intelligence could be utilised or developed for involvement in any of steps 1 to 11 (see main text).

**FIGURE 2:**
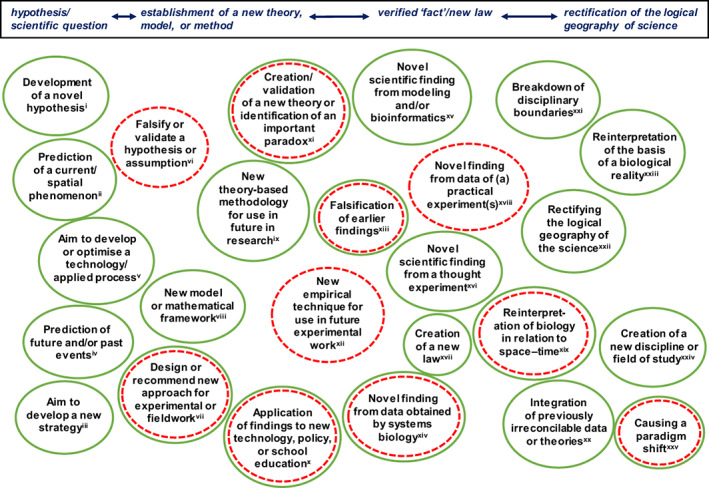
Some types of scientific findings/novelty (i to xxv) that published studies can yield; N.B., points i to xxv are not necessarily mutually exclusive. Green indicates those most commonly originating from theoretical approaches; red dashes indicate those most often arising from practical experiments (see also Figure 1); but theoretical methodologies and practical experiments often in reality co‐occur. Each is arranged approximately according to its degree of establishment (hypothesis/question versus theory versus established fact/law versus logical geography), but the scientific process is not always a clear linear progression, and we do not imply that establishing new facts or laws is necessarily less‐concrete than a rearrangement in the logic of science. Display entries i to xxv are pertinent to Ames (1939) [ii, iv, xi, xix, xxi, xxiii, xxiv]; Ryle (1949) [ii, vi, xi, xx‐xxiii, xxv]; Turing (1950) [i, ii, v, vi, ix, x, xvi, xxi, xxiv]; Anderson (1952) [i‐iv, vi, viii, ix, xi, xvi, xix‐xxv]; Sagan (1967) [i, iv, vi, ix, xi, xiii, xviii, xix, xxii, xxiii, xxv]; Levinthal (1969) [i, ii, iv, vi, xi, xv, xvi, xix, xxiii]; Monod (1971) [i, iv, vi, xix, xxii, xxiii, xxv]; Schwartz and Cantor (1984) [ii, v, vii, xii, xvi, xxiv]; Clark (1994) [i, iv, xvi, xxi, xxv]; Clegg et al. (1998) [vii, ix, xx, xxi]; Hallsworth (1998) [i, ii, vi, xi, xiii, xxii, xxv]; McKay (2004) [iii, ix, x, xvi, xxiii, xxv]; Casadevall (2005) [i, iv, xi, xvi, xxii‐xxv]; Pedrós‐Alió (2006) [ii, iii, vii, xx, xxiii]; Ball (2008) [i, ii, iv, ix, xix, xxi‐xxiii, xxv]; Price (2009) [i, iv, vi, viii, xiii, xv, xix, xxi, xxiii, xxv]; Rockström et al. (2009) [i, ii, iv, x, xix, xxi, xxiii, xxv]; Partida‐Martínez and Heil (2011) [i, vi, vii, xvi, xxv]; Cray, Bell, et al. (2013) [i, ii, vi, xi, xxii, xxv]; Lloyd et al. (2013) [i‐iii, vi, vii, xviii]; Oren and Hallsworth (2014) [i, ii, iv, vi, ix, x, xvi, xviii, xix]; Price and Sowers (2004) [iv, vi, viii, ix, xviii, xix]; Cray, Houghton, et al. (2015) [ii‐iv, vii‐ix, xii, xviii, xix]; Schuur et al. (2015) [i‐iv, vi, ix, x, xix]; Hug et al. (2016) [ii, iv, viii, xv, xxii, xxiii, xxv]; Lennon et al. (2017) [ii, vi, xvi, xviii]; Williams et al. (2017) [i, ii, iv, vi, xv]; Banerjee et al. (2018) [i, ii, vii, xix, xxii, xxiii]; Cavicchioli et al. (2019) [ii, iv, x, xix, xxii‐xxv]; Cross et al. (2019) [iii, xii, xvi, xviii, xxii]; O'Malley et al. (2019) [i, iii, iv, vi, ix, xii, xv, xviii, xix, xxii, xxiii, xxv]; La Cono et al. (2020) [vi, xiii, xv, xviii, xxii]; McGenity et al. (2020) [iii, x, xxv]; Das et al. (2021) [i, iii, v, vi‐viii, xv]; Hallsworth (2021) [ii, vi, x, xvi]; Hallsworth, Koop, et al. (2021) [ii, iv, vi, ix, xii, xv, xx, xxi]; Lloyd (2021) [vi, xi, xvi, xix, xxii‐xxv]; Mestre and Höfer (2021) [i, ii, iv, xi, xvi, xix, xxi‐xxiii, xxv]; Pedrós‐Alió (2021) [ii, iv, vi, xix, xxii, xxiii, xxv]; Timmis and Ramos (2021) [ii, iii, iv, vii, x, xxi, xxii]; Gao and Wu (2022a) [i, iv, vi, xv, xix, xii, xiii, xv]; Giovannelli et al. (2022) [i‐iv, vii, ix, xix, xxi, xxiv, xxv]; Hallsworth (2022) [i, ii, iv, vi, xi, xix, xxi‐xxv]; Mills et al. (2022) [i, v, vi, xv, xviii]; and Timmis and Hallsworth (2022) [i, iv, vi, x, xvi, xxi, xxiii].

Even those fields dominated by practical experimentation—known as ‘wet biology’ (Kahlem & Birney, 2006; Zannoni, 2018)—can benefit from alternative approaches such as thought experiments or other concept‐based and theory‐based studies. Indeed, there are instances when practical experimentation is not useful to address a scientific question, for example about the metabolism and physiology of uncultivatable microbes (Williams et al., 2017). Some of the most‐novel scientific findings were made during individual studies that used thought‐based approaches without any further practical experiments (e.g., Sagan, 1967). Such studies are nevertheless rational and empirical in as much as they draw from observation, earlier data (from fieldwork, laboratory experiments, etc.), or other types of evidence. In fact, all types of scientific study usually involve the same key elements of the scientific process (Figures 1 and 2). These include identification of a gap in current knowledge and formulating a question or hypothesis; devising an approach to test or address this (which involves the use of scientific controls where appropriate); carrying out the study to generate data/evidence; identifying novel scientific findings; drawing conclusions in relation to the initial aim/hypothesis (Figure 1; Prosser, 2022).

This said, some in the scientific community do not utilise the full range of research approaches available. For example, some of our colleagues and collaborators who are experimentalists and/or focus on applied research have expressed cynicism about studies that are theory‐based (see also Norton, 2004a; Weber, 2004, 2018). This is consistent with the views of some institutions that consider research articles as, by definition, original ‘primary literature’ and review articles as, by definition, descriptive or unoriginal ‘secondary literature’ (Supplementary Text ‘Institutional views of research articles versus review articles’). Some papers about the nature of knowledge also detail the degree to which theory‐based approaches can be overlooked by many research scientists (Howe, 2004; Schlaepfer & Weber, 2018) (though this can also occur with published studies of practical experiments).

We ourselves have heard colleagues and collaborators dismiss innovative studies that yielded novel scientific findings that were paradigm‐shifting—but were not derived directly from practical experiments—as ‘nothing more than ideas’, ‘qualitative science’ or ‘akin to the social sciences’; on a previous occasion, one colleague even asked, ‘how were you able to publish an article without any hard data?’. Similarly, one of our collaborators who describes themself as an experimentalist said of thought‐based or other kinds of theory‐based studies that they are carried out more rapidly than practical experiments so can be of low quality and ‘can damage the idea of science’. We agree that any rushed work—practical experiments or theory‐based—is likely to be of compromised quality. However, some of the theory‐based work highlighted in the current article (e.g., Cray, Bell, et al., 2013 and those by Casadevall) spanned almost two decades. To take a famous example, some studies by Charles R. Darwin took 20–40 years to carry out and publish (van Wyhe, 2007). At times, practical experimental studies have been regarded as ‘basic research’ in juxtaposition to other types of studies that were not (Zannoni, 2018). However, the studies highlighted in the current article have made advances in biology despite, or more accurately because of, their theory‐based approaches. This is true both for those works highlighted that were carried out in silico (Das et al., 2021; Gao & Wu, 2022a; Hug et al., 2016; O'Malley et al., 2019; Williams et al., 2017) and those that were not (e.g., Ball, 2008; Casadevall, 2005; Cray, Bell, et al., 2013; Hallsworth, 1998; McKay, 2004; Mestre & Höfer, 2021; Monod, 1971; Partida‐Martínez & Heil, 2011; Price, 2009; Price & Sowers, 2004; Sagan, 1967; Schuur et al., 2015).

Not only is thought an integral part of practical experimentation, but experimental data feed scientific thinking; and theory‐based science creates new lines of practical experimentation (Figure 1; Goldstein, 2022; Prosser, 2022). Furthermore, some of the most‐acclaimed scientific works were thought‐based. These include Darwin's book *On the Origin of Species by Means of Natural Selection, or the Preservation of Favoured Races in the Struggle for Life* (Darwin, 1859); Einstein's paper on *E* = *mc* ^2^ (Einstein, 1905); the theory of endosymbiosis of Lynn Margulis (Margulis, 1970; Sagan, 1967); Agnes Arber's book *The Natural Philosophy of Plant Form* (Arber, 1950); the paper that pre‐dates artificial intelligence about whether machines/computers can think (Turing, 1950); Jacques Monod's work of natural philosophy explaining how life arose from chance events *Chance and Necessity: Essay on the Natural Philosophy of Modern Biology* (Monod, 1971); the papers that elaborated the Gaia hypothesis (Lovelock, 1972; Lovelock & Margulis, 1974, see Urgent global challenges below); Gilbert Ryle's book *The Concept of Mind* (Ryle, 1949); and the paper that elucidated the structure of DNA (Watson & Crick, 1953). There seems to be a contradiction therefore between the general recognition that scientific novelty emerges from theory‐based studies such as these and the notion of scientific outputs that do not report practical experiments as, by definition, secondary literature.

### Thought (along with chance) drives the scientific process

During the progression of scientific knowledge as a whole, there is the proverbial chicken‐and‐egg question: does scientific thinking necessarily come first or practical experimental work? Whereas both often seem to come together, practical science usually involves the formulation of a question or hypothesis (through intuition and/or thought) followed by the design of appropriate and robust experiments (based on lucid scientific thought), then the analysis and interpretation of data to reveal scientific findings (again, thought is essential), and culminating in conclusions and predictions or suggestions for further work (all of which are dependent on the clarity of scientific thought; Figure 1). In addition, of course, chance and serendipity can play key roles in the scientific process. This said, it is the ability of the alert and thoughtful researcher to recognise novelty within a chance event that can make the difference between a profound discovery or none at all (Beveridge, 1950; Copeland, 2019; Foletti & Fais, 2019). It may be that the most‐famous example of this in (experimental) biology is the discovery of penicillin by Sir Alexander Fleming (Fleming, 1929; Foletti & Fais, 2019). In terms of serendipitous circumstances, recent examples include the opportunities provided due to the (otherwise disastrous) COVID‐19 pandemic, as described, for example, for the Mestre and Höfer (2021) study in section the Re‐evaluating space and time section of the current manuscript, below.

### COVID‐19 and the current article

The recent coronavirus pandemic (COVID‐19) has resulted in prolonged periods of lockdown worldwide, throughout the period from 2020 to 2022 (Coccia, 2022; Diffenbaugh et al., 2020), during which time many scientists, including the authors of the current manuscript, have been unable to access their laboratories. This has caused considerable disruption of the scientific enterprise (Deryugina et al., 2021; Korbel & Stegle, 2020; Myers et al., 2020), with an overall reduction in the number of new research projects initiated during the pandemic (Gao et al., 2021). Undergraduate research projects have in general had to be compromised or cancelled, yet they are designed to provide early‐career research opportunities and boost students' motivation and confidence and are integral to a student's degree programme. Mentors of university‐student research projects have needed to provide ‘dry’ research projects that can be carried out remotely and/or virtually (Elmer & Durocher, 2020; Gao et al., 2021).

Resources were developed to help students and supervisors move their research forward in settings remote from the laboratory, including the design of research‐focused projects based on systematic reviews and meta‐analyses of published data and other forms of literature review, mathematical modelling and computer simulations, data‐mining, and analyses of other types of previously collected datasets (Elmer & Durocher, 2020; Lewis, 2020). Designing a virtual research project can be a challenge for some research topics and for academics who usually depend on wet‐laboratory experimentation; even more so because, in many cases, some prior knowledge of practical scientific work is needed for most students to be able to engage in theoretical processes. However, this pandemic‐induced situation provides an opportunity to teach students to generate and progress scientific knowledge in ways other than wet‐laboratory experimentation.^1^ It is the experience of the current authors that the added time and energy available to those working from home under lockdown, and the need to produce outputs, has likely encouraged theory‐based studies that are not based directly on laboratory‐derived data (e.g., Hallsworth, Koop, et al., 2021; Lloyd, 2021; Pedrós‐Alió, 2021; Mestre & Höfer, 2021; see below). Paradoxically, the global human‐health catastrophe and economic crisis caused by COVID‐19 might have encouraged additional flexibility and creativity within the scientific process.

The authors of the current article are experimental biologists or geologists, with several of us also involved in field observation (see Supplementary Text ‘Research foci of the authors’). However, we are not experimentalists who value practical experimentation to the exclusion of theory‐based work. Here, we argue that scientific progress depends on scientific thought in addition to—and at times instead of—practical experimentation. We appraise the roles of theory‐based approaches in microbiology focusing largely on ‘wet biology’ areas of microbiology that are usually led by practical experiments—such as growth, survival, cell physiology, competitive and symbiotic interactions between microbes, and host–pathogen interactions—but also include some studies in the fields of bioinformatics, planetary health and astrobiology. We focus primarily on individual microbiology‐related studies that pose a novel scientific question or hypothesis and reveal new scientific findings without carrying out practical experiments.

Interviewing the authors of innovative papers, and drawing from our own research experiences, we show that scientific novelty can come from theory‐based research whether carried out alone or in combination with wet‐biology experiments or practical fieldwork. We augment these arguments with comments on the roles of thought experiments and natural philosophy, logic and linguistics in microbiological research; using theory when this is the most‐scientifically robust and most‐direct route to scientific novelty, whether practical experiments or not; and the potential to enhance human creativity in scientific innovation with artificial intelligence technology (though these categorisations are not mutually exclusive). The current article is not primarily a study of the philosophy of science, but is a ‘situational–functional’ examination of the value of thought experiments and other types of studies beyond the experiment can have for the science of microbiology, especially in wet‐biology research areas. It does not in any way propose that microbiology as a whole would benefit from less practical experimentation, but rather argues that some research topics might benefit from greater use of studies based on thought experiments and other types of theory‐based approaches. For example, we also propose that some current global challenges that require urgent solutions—not least anthropogenic climate change—can benefit from scientific approaches beyond the experiment.

## THOUGHT EXPERIMENTS

Practical laboratory work and/or fieldwork are not necessarily implicit in the concept of the experiment.^2^ For instance, there are thought‐based experiments that typically focus on manipulating a variable, albeit mentally, to see what might happen. Whatever the type of research approach, the formulation of a hypothesis provides a proposition that acts as a starting point for further investigation. Hypotheses, if/once supported by sufficient data, can progress to be regarded as a theory which is a supposition or set of ideas/principles that can explain an issue (and are usually underpinned by some evidence: Figure 2; Beveridge, 1950). Ultimately, if rigorously and universally validated/verified, such knowledge becomes known as fact, or—if a statement of a principle or equation—becomes known as a law (Figure 2). At each stage (whether hypothesis, theory, or law), further evidence or knowledge can be acquired via thought‐based or other theory‐based approaches, laboratory experimentation, field observations, other sources of evidence, or a combination of these (Figures 1 and 2; Beveridge, 1950; Goldstein, 2022).

The use of thought experiments in science has a long and distinguished pedigree; thought experiments likely have been carried out in some form or another throughout human history (for examples of classical thought experiments, see Box 1 and Sorensen, 1992). Notably, some thought experiments generate data including Levinthal (1969) (Box 1), as do some studies that produce models (Picoche & Barraquand, 2022) or examine the quantitative feasibility of proposed experimental work (Carney et al., 2020). Other theoretical approaches that generate data include studies of growth kinetics where exponential growth is assumed to occur via a doubling of cell number based on identical cells dividing into two and assuming no limitation due to nutrients or stress(es); this generates the quantitative prediction of exponential growth and, if doubling time is known, a quantitative prediction of specific growth rate. In relation to microbiology, more‐recent thought experiments have been carried out on diverse topics including dinosaur‐versus‐mammal evolution (Casadevall, 2005); plant–microbe interactions (Partida‐Martínez & Heil, 2011); metal cycling in the deep Earth (Edmonds et al., 2018); temporal aspects of the microbial biosphere (Box 2); the origins and tenacity of microbial life (Price, 2009); global ecology and planetary function (Lovelock & Margulis, 1974); potential impacts of climate change (Timmis & Hallsworth, 2022); and astrobiology (Clark, 1994; McKay, 2004).

BOX 1 **Examples of classical thought experiments.^3^**

Famously, the thought experiment of Galileo di V.B. de’ Galilei that involved balls rolling across a sloping surface in the absence of friction effectively led to the understanding of inertia that was later developed by Sir Isaac Newton (Einstein & Infeld, 1938). The thought experiments of Albert Einstein are legendary and ultimately led to the theory of general relativity. Another classic thought experiment, best known as ‘Schrödinger's cat’, was devised by Erwin R.J.A. Schrödinger to illustrate quantum superposition (Schrödinger, 1935). Other theory‐based articles in the discipline of physics have yielded profound findings, not least the work by Nobel Prize winners François Englert and Peter W. Higgs who predicted the existence of the Higgs boson subatomic particle (Englert & Brout, 1964; Higgs, 1964), which was confirmed empirically in 2012 after which the Nobel Prize was awarded to Englert and Higgs. Another example in physics is the paper of Steven Weinberg on the unification of weak and electromagnetic interactions (Weinberg, 1977) for which he also won the Nobel Prize. Even in present‐day physics, a considerable fraction of the science has not been demonstrated experimentally (e.g., dark energy).

In biochemistry, the so‐called Levinthal's Paradox is a classic and original thought‐based study that subsequently inspired different experimental approaches to answer the proposed question: ‘How to fold graciously’ (Levinthal, 1969). In his communication to a meeting on the use of Mössbauer spectroscopy in biological systems, Cyrus Levinthal presented his calculations of possible conformations for a large protein (Levinthal, 1969). In these calculations—if we consider a small protein with 101 residues and for each residue, we assume three different conformations—it would take 10^27^ years for the protein to try out all its possible states before finding its optimal configuration (at a rate of 10^13^ configurations per second). This in turn indicates that if proteins fold via random motions, the timescales to achieve their functional in‐vivo conformations would be implausibly long, implying that in reality there must be a folding system that allows proteins to obtain the biologically functional conformation in a rapid and efficient way. According to Toews (2022): ‘To frame that figure more vividly, it would take longer than the age of the universe for a protein to fold into every configuration available to it, even if it attempted millions of configurations per second’.

The short length and straightforward nature of the Levinthal (1969) conference‐proceedings paper belie its importance as a pioneering and impactful research study of a phenomenon that underpins the metabolic processes of cellular systems. The protein‐folding conundrum is even more perplexing in complex systems like the ribosome, in which many proteins and nucleic acids have to interact in a proper conformation to generate a functional particle. Levinthal's suggestion was that the process is nucleated by local structures that fold rapidly. Advances in technology and algorithms have produced great advances in protein structure prediction, but protein folding is a much more‐complex problem to solve. Different computational strategies have been used since the proposal of the paradox but even though important progress has been made, like showing that the protein energy landscape is funnel‐shaped (Dill, 1987), to the best of our knowledge a solution to the paradox has not yet been obtained (Gershensen et al., 2020). However, artificial intelligence has gone some way to resolving this computing paradox (Toews, 2022).

Box 2 **The microbial relationship with time.**

Prior to 2021, one of us (C.P.‐A.) had been exploring the implications of a large rare biosphere (see the Supplementary Text: ‘Diversity and ecology of marine microbes’) during which time he realised that a portion was locked within ice yet ready to resume activity if re‐released. C.P.‐A. also realised that no existing research article systematically examined microbial survival in this way, albeit that pertinent experimental data already existed. Therefore, additional practical experiments would have been redundant. Furthermore, any new experiments would have also taken a considerable budget and some years to plan and execute. It is for these reasons that C.P.‐A. wrote the concept paper ‘Time travel in microorganisms’ (Pedrós‐Alió, 2021). It was his re‐evaluation of the survival and revivability of inactive microbes in ice (and other places) in the context of time travel that brought fresh insights into this aspect of microbiology, including the implications for infectious diseases, pan‐genomes, and the rare biosphere (Pedrós‐Alió, 2021).

Consistent with Pedrós‐Alió (2021) is the Crystal Ball article written by one of us (K.G.L.): ‘Time as a microbial resource’ that focuses on the strategic behaviours of microbes that move through time via long periods of inactivity (Lloyd, 2021). The article's short title goes a long way towards explaining this potent aspect of microbial ecology. K.G.L. had been thinking for some years about the implications of measurements that suggested 30‐ to 300‐year turnover times for cells within marine‐sediment microbial communities (Hoehler & Jørgensen, 2013) and wanted to make a detailed exploration of this phenomenon. The Lloyd (2021) study is a kind of thought experiment that effectively proposes the hypothesis that evolution arises from natural selection driven by extremely slow events, such as the gradual movement of tectonic plates. It concludes that a lack of an upper limit on lifespan means that the selective features driving the diversification of subsurface microbes may be similarly unlimited. The article by K.G.L. can potentially result in more researchers becoming less constrained by the timescales over which biology is assumed to act so that we expand future studies into other timescales: see also Price (2009), Müller et al. (2014), Hallsworth (2022), and Schreder‐Gomes et al. (2022). We predict that articles such as these will also stimulate modelling studies, fieldwork, and further lines of experimentation in the laboratory (Figure 2). One intriguing question, for example, is: what are the impacts of this suspended animation on the genome evolution of trapped microbes that are periodically re‐released into the biosphere?

The study ‘Fungal virulence, vertebrate endothermy, and dinosaur extinction: is there a connection?’ (Casadevall, 2005) put forward the hypothesis that differences in susceptibility to fungal infection—in particular the susceptibility of reptiles and stronger resistance of mammals—were responsible for the success of the mammalian lineage since the Cretaceous Era. As a consequence, once the dinosaurs were exterminated due to the cataclysmic impact of the Chicxulub meteorite (and its consequences), there was no second ‘Age of the Reptiles’.

We asked Arturo Casadevall (Johns Hopkins University, MD, USA) where the idea for this study had come from. For more than two decades, Casadevall had been fascinated by why fungal pathogens of mammals are rare whereas threats to ectothermic organisms such as plants, insects, and frogs and other ectothermic vertebrates are commonplace. Also, he was preoccupied with the thought that there had to be a selection mechanism to explain why reptile dominance was not restored following the Cretaceous–Tertiary extinction event but was instead replaced with endothermic mammals despite their lower rates of reproduction and higher energy requirement—to maintain higher body temperatures—relative to the reptiles. Given that most fungal species are not able to thrive at mammalian body temperatures (Robert & Casadevall, 2009), an emergence of fungal infections in reptiles following the Cretaceous–Tertiary extinction might have contributed to the selection pressure for the rise of mammals. Hence, Casadevall elaborated his hypothesis (Figure 2; Casadevall, 2005), which has since led to further studies and is now regarded as a theory (Casadevall, 2012, 2016; Casadevall & Damman, 2020; Robert & Casadevall, 2009). It is difficult to do practical experiments for temporally past geologic events that are now remote (only inference from the rock or fossil data is possible). Nevertheless, this microbiology thought‐experiment became an investigation in which diverse lines of evidence converged into a novel scientific finding that is profound, especially for humans, because it ultimately facilitated the emergence and success of humans as a species. It also presents an explanation for the emergence of endothermy in biology.

The theory has considerable explanatory power but also makes worrisome predictions. According to Casadevall, the remarkable resistance of humans to fungal disease is due to the twin pillars of their relatively high body temperature and advanced immunity (Casadevall, 2020). Fungal diseases did not emerge as a significant medical problem until the mid‐20th Century when advanced medical procedures and drug therapies resulted in a high number of immunocompromised individuals who had lost their immunity pillar. Losing the temperature pillar is unlikely since humans cannot survive colder body temperatures for a long time. However, the temperature pillar can be defeated if fungal species with pathogenic potential adapt to mammalian body temperatures. Although most fungal species cannot tolerate (or at least do not thrive) at temperatures approaching 40°C, and are thus not pathogenic for mammals (Robert & Casadevall, 2009), global warming could plausibly lead some species to evolve adaptions to higher temperatures (Garcia‐Solache & Casadevall, 2010). Casadevall et al. (2019) recently proposed that the sudden appearance of a new fungal pathogen *Candida auris* was facilitated or driven by global warming and, if this is correct, we can likely expect more fungal diseases to emerge during the current century.

The field of astrobiology in particular benefits from science not based on practical experimentation, including various types of thought experiments, as exemplified by the studies of McKay (2004), below, and Clark (1994), in the Supplementary Text ‘Acid brines and habitability of Mars’. Another thought experiment, by Partida‐Martínez and Heil (2011), considered the role of microbes in plant health: ’The microbe‐free plant: fact or artefact?’. We asked Laila P. Partida‐Martínez (Cinvestav Irapuato Unit at Center for Research and Advanced Studies of the National Polytechnic Institute, Irapuato, Mexico) how this study came about. She was inspired by the realisation that symbioses are everywhere, whereas there is a widely held assumption that they are rather the exception. Partida‐Martínez had worked, for example, with the plant‐associated fungus *Rhizopus microspores*, and found that this fungus also lives in association with bacteria. The study of Partida‐Martínez and Heil (2011) considered mycorrhizal fungi and *Rhizobium* and other diazotrophs, as well as bacterial and fungal endophytes. It found that microbes can impose a cost on the host plant, but that there are overwhelming benefits in relation to plant nutrition, plant‐growth rate, plant resistance to stresses, and plant survival and reproductive success. These are affected by diverse mechanisms, some of which are direct and others indirect, and the costs and benefits of the microbiome are complex, dynamic, and context‐dependent (Partida‐Martínez & Heil, 2011). The authors concluded that the microbe‐free plant is an artefact (used, for instance, in some experiments) and advised that it would not be suitable to use as experimental control in practical experimental studies.

The Partida‐Martínez and Heil (2011) study also has implications for the microbiomes of other eukaryotic organisms that are dependent on microbes, including humans, and what conditions facilitate the emergence of pathogenic activity. At the time that the thought experiment was carried out, terms such as ‘plant holobiont’ and ‘plant microbiome’ were not commonplace and people did not think widely about whether plant health was dependent on microorganisms. The study has thus far been cited 400 times (Google Scholar; December, 2022). Since the time of its publication, Partida‐Martínez has been interested in plant holobionts and started deciphering the microbiomes of *Agave* species and cactus species. Her studies on these desert plants have described the phylogenetic diversity within their microbiomes (Coleman‐Derr et al., 2016; Fonseca‐García et al., 2016), and their functions including the discovery of microbial volatiles capable of promoting plant growth and the formulation of synthetic communities that impact microbial diversity and plant fitness (Camarena‐Pozos et al., 2019, 2021; Flores‐Núñez et al., 2020, 2022). Partida‐Martínez and her group also discovered that *Rhizopus microsporus* harbours both bacterial and viral symbionts and that both are important for its biology (Espino‐Vázquez et al., 2020). The article of Partida‐Martínez and Heil (2011) has contributed to the understanding that plants (especially) are not individual isolated organisms, but are effectively holobionts that are an assemblage of taxa functioning as an ecophysiological unit (Flores‐Núñez et al., 2022).

Thought experiments typically utilise data from practical experiments, observations, and/or other empirical measurements. They can give rise to new lines of practical experimentation as evidenced by the hundreds of research articles that have already cited the studies of Partida‐Martínez and Heil (2011) and Clark (1994), Casadevall (2005), and McKay (2004) that are discussed elsewhere in the current article. Some interesting findings and speculations are overlooked or ignored because they are advanced for their time; see the comments about McKay (2004) below and those about Ames (1939) in the Supplementary Text ‘Oakes Ames (1874‐1950)’. This was also the case of Darwin's premonitions about life in different extreme environments based on his observations of microorganisms in a sample from an Argentinian salt pond (Darwin, 1839). Although future experiments searching for the limits of life, which led Woese and Fox (1977) to discover the *Archaea*, did not recognise the importance of Darwin's premonitions, recent research searching for life in the oligotrophic deep continental subsurface (Escudero et al., 2018) has recognised the veracity of Darwin's speculations, which after 200 years have been entirely validated. Each of these thought‐experiment studies has redefined the way that we understand the gestalt of a topic/field and, in this way, each has redefined its respective paradigm.

### Studies that are highly provocative

Some thought‐based studies, carried out to address an important scientific question, conclude that their respective question could not be answered. These include studies relating to the nature of life such as those of Schrödinger (1944), Margulis and Sagan (1995), and McKay (2004). These and other cases—including those thought‐based studies mentioned above—can nevertheless drive scientific progress by posing an unresolved question or conundrum that stimulates new lines of experimentation. Some such questions have such universal appeal and potency that they capture the attention and provoke the thoughts of scientists across disciplines as well as the general public.

The study by Christopher P. McKay (NASA Ames Research Center, CA, USA) focused on the search for life elsewhere in the Solar System: ‘What is life—and how do we search for it in other worlds?’ (McKay, 2004). Importantly, McKay systematically identifies the properties of structural molecules used by life on Earth—such as amino acids and lipids—as key targets for life detection, and offers a way to distinguish a second genesis of life. This was a sharp departure from the growth‐based methods used for life detection in the 1970 s Viking missions to Mars (the only previous example of a life‐search mission) and has led to a new paradigm in life detection (Figure 2).

McKay explained to us that the approach of his 2004 study was in contrast to the search for morphological fossils, such as stromatolites, as the basis for life detection advocated by planetary geologists. Instead, it built on the suggestion of James E. Lovelock who proposed a molecular method using lipids, rather than a growth method, for the search for life on Mars (Lovelock, 1965). At the time of the McKay (2004) article, there was little focus on life‐detection missions so the paper received few citations and, in general, attracted little attention. However, interest grew dramatically a few years later as missions for life detection to Enceladus, Europa, and Mars were developed. It is therefore not surprising that Hou and Yang (2020) identified this paper as an example of a ‘Sleeping Beauty’ a paper that receives little notice for five or more years and then experiences rapid growth in citations and social‐media activity.

Whereas the work of Lovelock inspired the study of McKay (2004), one of Lovelock's most‐original ideas to detect signs of life on Mars was perhaps the proposal to detect atmosphere composition and thereby evaluate its chemical disequilibrium. Mars's atmosphere composition, as detected by radio astronomy, was almost in chemical equilibrium—in contrast with that of the Earth—which allowed him to conclude the absence of life on Mars (Lovelock & Giffin, 1969). Like many research papers that represent a step change, the work of Lovelock was often highly provocative, including his work relating to Gaia which is discussed below. Other thought‐based studies that pose provocative scientific questions include Turing (1950), Sagan (1967), Levinthal (1969), Casadevall (2005); Partida‐Martínez and Heil (2011), Cavicchioli et al. (2019) and Hallsworth (2022), which are discussed in the current manuscript, and Martin and Müller (1998), Danchin (2021) and Lauber et al. (2021).

## NEW PARADIGMS BY RECTIFYING LOGICAL GEOGRAPHY

Changing a paradigm is, as Kenneth Timmis (Technical University of Braunschweig, Germany) once remarked to one of us (John E. Hallsworth; J.E.H.), ‘the greatest thing in science’ (see also Figure 2). This can occur via thought experiments or practical experiments, but some paradigm shifts occur via studies that make a logical rearrangement of existing knowledge in a particular area of science.

There are few better examples of this than the treatise ‘Water as an active constituent in cell biology’ by Ball (2008). Physiologically active cells are primarily composed of water, and this water is intimately involved in virtually every structural and functional interaction/reaction within the entire system, including those driven by hydrophobic forces. Water pervades, and acts throughout, the entire cell at all levels; from thermodynamics to ecological activities and, not least, because of the electron distribution within water molecules. However, the avid reader of many biochemistry or molecular biology texts might sometimes be forgiven for not realising that water is even present. This is because, ever since the inception of modern biochemistry, the cell has been widely viewed as a structure composed of biomacromolecules with reactions driven by enzymes, a metabolism that occurs via various pathways and biochemical reactions with nucleic acids as part of this biomacromolecule‐driven structural–functional view of the cell. In general, research studies and textbooks have not acknowledged that these activities all take place within an aqueous matrix (and its hydrophobic domains) that virtually without exception entails the intimate involvement of water molecules.

There have been some seminal texts relating in some way or other to water, not least *The Structure and Properties of Water* (Eisenberg & Kouzmann, 1969) and *Microbial Water Stress Physiology: Principles and Perspectives* (Brown, 1990). However, it was not until 2008 that the entire cell and its metabolic activities were reinterpreted in the context of the water that pervades, partakes in, and controls much of the cellular system (Ball, 2008). Philip Ball (London, UK), with a BSc in Chemistry and a PhD in physics, is an expert in the role of water within living systems. We asked Ball what motivated the 2008 study and he explained that after having written the book *H*_*2*_ *O: A Biography of Water* (Ball, 1999), he was invited to a small meeting in Italy in 2004. This meeting was convened to discuss the question of whether water is a ‘biophilic’ molecule that seems uniquely attuned to supporting life; revisiting the question first posed by Lawrence Henderson in *The Fitness of the Environment* (Henderson, 1913). Ball was most fascinated by the role of water in living cells, so focused his talk at the 2004 meeting on this topic. He later decided to try to publish the wider story as a review article that integrated and interpreted much of what was then known on the question of how water features in molecular and cell biology (Ball, 2008).

Ball (2008) argued that water is not, as often presented in the textbooks, a passive backdrop and solvent in which life's molecular processes unfold. Rather, it is an active constituent in those processes, in a host of ways. For this reason, we cannot truly consider molecular mechanisms in biology without taking explicit account of the role of solvation, sometimes at the resolution of individual water molecules. The article has already been cited more than 1600 times according to the publisher's website (December, 2022), and has almost certainly helped focus a broad and diverse research community on the importance of hydration and water dynamics for biomolecular function. The field has only grown since the 2008 paper, as reflected for example in the highly successful and international RESOLV program coordinated by the Ruhr University Bochum (Germany), which aims to establish the discipline of ‘solvation science’, with applications ranging from cell biology to industrial catalysis and electrochemistry. Ball explained to us that: ‘In my own mind, the considerations raised in the review article have also cast new light on the astrobiological aspects of water ‐ a topic on which I have written several times subsequently, and which is now (with the observation of exoplanet atmospheres) more relevant than ever’.

### Re‐evaluating space and time

Biological processes can occur over vast timescales, yet practical experimentation is in general restricted to the present. Nevertheless, theory‐based studies have characterised key aspects of the relationship of microbes with time, and the potential microbiome of a future Earth in the event of a runaway greenhouse effect (Timmis & Hallsworth, 2022; see Urgent global challenges below). The human senses relate primarily to spatial phenomena. As explained by Klein (2004), whereas we are not able to experience time itself (we cannot directly hear, see, smell, taste, or touch time), we instead experience other things through time. We do, therefore, know the consequences of time first‐hand, such as the duration of things, but not time itself. Thus, we need abstraction beyond the physical world—usually at a higher cognitive level—to imagine time in (what feels like to us) a more‐direct, more‐immediate sense (Klein, 2004). The authors of the current article believe that researchers too relate readily to spatial aspects of microbiology, but have to conceptualise the temporal aspects. Therefore, scientific terminology tends to be more space‐orientated than time‐orientated and this in turn reinforces our spatially‐biased thinking.

Examples include terms such as ‘ecosystem’, ‘habitat’, and ‘biosphere’ that are biased, in as much as they give a sense of entities that *exist* (in space) rather than *occur* (in time). They in turn reinforce our spatially‐biased worldview that microorganisms exist in a specific place rather than the more accurate view that microorganisms are temporally subject to conditions (or sets of conditions) that in some cases change continually. Do we, therefore, accept that a microbe's ‘habitat’ may change, possibly even on a timescale of less than 1 second? Or, do we now try to abandon what seems to be a 19th‐Century culture amongst biologists of defining an organism by/within a specific habitat? Arguably, the concept of an organism's habitat as a location is not conducive to properly understanding the temporal aspects of the biology of many of the microbial systems in nature.

This problem was recently discussed in the context of water activity [the effective concentration of water molecules]: ‘The water‐activity boundary for [active] life is, in terms of thermodynamics, a concrete phenomenon, yet one that can be both dynamic and ephemeral. For example, the water of some microbial habitats that contain sugar (e.g., nectar; Lievens et al., 2015; Witt et al., 2013) can evaporate causing rapid and profound changes in water activity. The same phenomenon occurs in sea spray, on rock surfaces, and in hypersaline brine systems (Benison et al., 2007; Michaud et al., 2014). Similarly, microbes within bioaerosols may experience being in pure water (water activity = 1) and as the droplet evaporates, the cell may be without liquid water (Mao et al., 2007; Verreault et al., 2008); [it, therefore, passes through the entire thermodynamic range for an active life on Earth, and can do so within a fraction of a second]. This illustrates how fraught the notion of a spatial limit for life can be. It also underlines that the edge of Earth's biosphere is [often being played out] all around us…’ (Hallsworth, 2019).

Numerous examples of using time (rather than spatially‐oriented concepts) as the primary lens through which to analyse microbiology‐related science are discussed in the current article (e.g., Casadevall, 2005; Hug et al., 2016; McKay, 2004; Monod, 1971; O'Malley et al., 2019; Price & Sowers, 2004; Rockström et al., 2009; Schuur et al., 2015; Timmis & Hallsworth, 2022). In other examples, theoretical limits have been estimated for the apparently indefinite time periods that microbes survive within ice, brines, and other environments (Price, 2009), and recent papers focused on how microbes use time to their advantage (Box 2) and the way in which water acts to preserve their cells (Hallsworth, 2022). Other studies focus on time in relation to protein folding (Levinthal, 1969), microbial interactions (Cray, Houghton, et al., 2015), evolutionary biology (Gao & Wu, 2022a; Sagan, 1967), climate change (Cavicchioli et al., 2019), and planetary‐scale microbial ecology (Mestre & Höfer, 2021).

The study by one of us (Juan Höfer; J.H.) and his colleague ‘The Microbial Conveyor Belt: connecting the globe through dispersion and dormancy’ is about the way in which there is an interplay between space and time in relation to how and when microbes function as they circulate around the environment (Mestre & Höfer, 2021). It focuses on the ecology of microbes that proliferate only at moments in time/space when conditions are favourable. The authors realised that an idea of Mestre's regarding the dispersion of marine microbes (based on her previous work) could be expanded to the whole biosphere, and realised that it resembled the concept of global biogeochemical cycles. Mestre and Höfer then went on to investigate common points between these two concepts while thinking about the implications of the idea and looking for previous findings in the published literature that might support it.

The main thesis/theory is that dormancy and dispersal of microbes, and the moments in time when they become active, are key drivers of ecosystem function throughout much of Earth's biosphere. The authors termed this *The Microbial Conveyor Belt* (Mestre & Höfer, 2021). Various ecological–evolutionary implications follow from this idea—that is arguably so well‐supported by previous lines of evidence that it could be regarded as a theory—including that microbial dispersion is not always random/stochastic because it has been, and is, to some degree selective for specific microorganisms. It also leads to the question of how important the Microbial Conveyor Belt has been in the evolutionary trajectories of both microbes and ecosystems. Similarly, the implications of this process for the global resilience of ecosystem functioning are important and need to be addressed in the context of increasing anthropogenic impacts on environmental and planetary health. Given the cross‐disciplinary implications of the Mestre and Höfer (2021) study, it will likely also inspire future experimentation in research areas such as ecology, microbiology, evolutionary biology, oceanography, geology, atmosphere science, and astrobiology. We believe it likely that this study has provides a useful framework for others to explain their past and future results (e.g., Gittins et al., 2022).

Mestre and Höfer (2021) author J.H. is quite certain that publishing the paper would have been very difficult—perhaps impossible—without the world slowing down during the COVID‐19 lockdowns; Mestre and Höfer had the opportunity to sit and discuss ideas more frequently because they spent the lockdown together with little distraction for a period of ~6 months after returning from Antarctic fieldwork. This (involuntary) team‐building retreat gave Mestre and Höfer extra time to reach the final publishable theory; the pandemic had created a fertile environment/time in which these ideas proliferated. This—quite fittingly—mimics the periodic proliferation(s) of life within the Microbial Conveyor Belt itself.

Also related to space and time is the research study of J.E.H. ‘Water is a preservative of microbes’ (Hallsworth, 2022). He was inspired to think about this topic around 2014 when considering the long‐term survival of microbial cells trapped in the hypersaline fluid inclusions of mineralised NaCl (halite). It occurred to him that, even in NaCl‐saturated brine, ions have a hydration shell so are not usually in direct contact with the cell's macromolecular systems which, conversely, are for the most part also hydrated. So, most of the interactions between ions and biomacromolecules are mediated by water. Indeed, much of what happens in a cell is mediated by water as described above (Ball, 2008; see also Ball, 2013; Ball, 2017a; Bosch et al., 2021; Brown, 1990; Crowe et al., 1992; Hallsworth, 2018; Maurer & Oostenbrink, 2019). It seemed to J.E.H. that water is likely the active principle that preserves the structure and viability of the microbial cell in brines. Furthermore, water in the form of water‐ice preserves cells in a viable condition, whether in −70°C freezers or in permafrost and other parts of Earth's cryosphere. Whereas some halophile experts believed that it is just the salts (not the water) that in some way preserve cells, some psychrophile experts believed that it is simply low temperature (not the water) that preserves those cells within the ice.

However, there is evidence that H_2_O facilitates the long‐term survival of microbial cells in all the basic types of water‐based milieux: pure water, freshwater systems, seawater, brines, ice/permafrost, sugar‐rich aqueous milieux, and even in vapour‐phase water (Hallsworth, 2022) including circumstantial evidence that viable cells may survive in brines for timescales measured in hundreds of millions of years (e.g., Schreder‐Gomes et al., 2022). Via a theory‐based analysis, J.E.H. also identified the modes‐of‐action of water as a preservative that operate at various scales, from the molecular level to the planetary scale. Water maintains biomacromolecular systems and the cell's structural integrity, buffers against thermodynamic extremes (in part due to its low specific gravity and high specific heat capacity), mitigates against events that can rupture the cell membrane (desiccation–rehydration, freeze–thawing, thermal shock, etc.), reduces oxidative damage by preventing cellular dehydration, reduces the penetration of ultraviolet radiation, dilutes solute stressors and toxic substances, is good at electrostatic screening thus preventing damage to the cell by the electrostatic fields of some ions, and mediates moderate cellular stresses that can invigorate and rejuvenate cells, etc. (Hallsworth, 2022). In addition, water acts effectively as a buffer of the water activity of those saturated brines that are dominated by a single salt via the dissolution and precipitation of the salt as temperature changes (Winston & Bates, 1960), thus protecting microbes in brines against extremes.

The paper also acknowledges the paradox that water in some ways can be deleterious to the cell; like oxygen, water can act to damage/destroy as well as to facilitate and maintain life (Hallsworth, 2022). Amongst other implications, the study re‐evaluated the microbiology of space and time (see also Figure 2) and discusses the large‐scale release of preserved microbes—including those released from melting permafrost—that is caused by global climate change. Climate change is also causing the reactivation of large‐scale microbiomes due to nutrient release into environments that were hitherto nutrient‐deplete (Hallsworth, 2022). Whereas the idea for this study preceded the COVID‐19 pandemic, it was the lockdowns that provided the time and opportunity to carry out this research work.

Two ground‐breaking papers by physicist P. Buford Price (University of California, Berkeley, CA, USA) explored the tenacity and persistence of psychrophilic microorganisms in the context of space and time, thus contributing to knowledge of the spatio‐temporal limits of Earth's functional biosphere: ‘Microbial genesis, life and death in glacial ice’ (Price, 2009) and ‘Temperature dependence of metabolic rates for microbial growth, maintenance, and survival’ (Price & Sowers, 2004). In the Price (2009) study, a thought‐experiment approach is used to reveal the possible ways in which microbes moved into, colonised, and persisted within the ice of Earth's cryosphere. In addition, arguments that nucleic acids might have originally formed within the ice are elaborated. Price (2009) uses Arrhenius calculations to determine that metabolic rates at subzero temperatures match rates of cell‐damage repair (in relation to DNA depurination and amino‐acid racemisation). One of the main implications is that microbial cells can survive in different types of ice potentially for millions of years.

In the earlier study, by Price and Sowers (2004), the authors also carried out analyses based on Arrhenius equations but these were for rates of microbial growth, metabolism, and biochemical reactions at subzero temperatures (see Figure 1 of their paper). The datasets used for this purpose, from previously published studies, included data for microorganisms from the ice‐covered Antarctic Lake Bonney, South Pole snow and ice, supercooled clouds, salt marsh, and marine sediment. The study determined that there is no absolute lower temperature limit for metabolism, but did derive an approximation for the low‐temperature limit for an active life on Earth based on the near‐zero rates of cellular activity (Price & Sowers, 2004). This value (−40°C) was recently used as the lower limit for life when Hallsworth, Koop, et al. (2021) designed their study relating to the habitability of planetary atmospheres described in Insights relating to thermodynamic parameters below.

### Natural philosophy can drive scientific progress

The thought experiments discussed above tended to take a more‐holistic (less‐reductionist) approach to scientific problems than is typical of many present‐day scientific studies. In a small fraction of life‐sciences studies, however, biological research is not carried out in isolation from philosophy (Box 3). In these cases, there is an undercurrent of inter‐ or transdisciplinarity such as in the works of Daniel C. Dennett III (Tufts University, MA, USA) and Maureen A. O'Malley (University of Sydney, Australia). Theory‐based studies can also enable transdisciplinarity such as in the case of microbiology‐related analyses of sociopolitical issues (Anand et al., 2023; Diamond, 2005) and education of school children (McGenity et al., 2020; Timmis et al., 2019). There is also a natural philosophy mode‐of‐inquiry employed in most of the studies highlighted in the current article. Collectively, they illustrate how thought experiments and other theory‐based studies can drive scientific progress (even in the present day) in ways that most practical experimentation cannot. For example, studies that utilise thought processes and/or theory‐based analyses frequently transcend the reductionism of modern science and mitigate against the channelled and constrained thought processes that language can create (see Inconsistencies in language and logic as a trigger for scientific investigation below). In this way, research studies not restricted to the framework(s) provided by practical experimentation can more easily enable the syncretistic integration and interpretation of polyphonic lines of evidence as described in Box 4 (see also Implications and perspectives sections). In doing so, such studies can lead to novel scientific findings and provide a more‐holistic comprehension of biology.

Box 3 **Natural philosophy is both classical and modern.**

Prior to the era of modern science, the study of the natural and physical world and the universe was known as the *natural sciences* (Cahan, 2003). Prior to this (from around the time of Aristotle to the 19th Century), the more‐holistic and essentially philosophy‐based study of the natural and physical world/universe was known as *natural philosophy* (Cahan, 2003). It should be noted that, prior to modern science, the term ‘philosophy’ retained its original meaning (the love of wisdom), and was not used in the narrow sense as it is today (the speculation‐based study of the nature of knowledge, reality, and existence). Natural philosophy predates the time when philosophy, science, and art came to be viewed as disparate domains and is now seen by some as a historical and anachronistic phenomenon (Lüthy, 2000).

In essence, even in the context of present‐day studies, natural philosophy describes an approach that is without the fragmentation—and the resulting artificiality, artifice, and boundaries that fragmentation causes—created by the separation of modern philosophy, modern science, and the modern arts; those subdivisions created by individual scientific disciplines; and the additional divisions caused by reductionism within modern science (Sloane, 1945). It is also noteworthy that published studies of the natural philosophy era (e.g., Darwin, 1839) were typified by a clarity of language and logical flow by comparison with many scientific papers of the present day. This may be because reductionism has encouraged many authors to write in an inaccessible, codified manner that only their immediate colleagues and peers—who work on the exact same topic—can understand (Ball, 2017b; Barnett & Doubleday, 2020; Chawla, 2020; Plavén‐Sigray et al., 2017).

The use of interdisciplinary and transdisciplinary approaches (Ng, 2022) to some extent can counter the fragmentation of science that has occurred since the inception of modern scientific research. Whereas multidisciplinary studies usually involve experts from different areas of science working in collaboration, interdisciplinary or transdisciplinary approaches represent a truly integrated and seamless activity within which (conceptually at least) elements are derived from diverse disciplines (Cockell, 2002; Nissani, 1995; Parro et al., 2020; Taşkın & Aydinoglu, 2015). To some degree, inter‐ and trans‐disciplinary research is a return towards natural philosophy and typifies the work of people regarded as polymaths or Renaissance intellectuals (see Supplementary Text ‘Inter‐ and trans‐disciplinary scientists).

The terms *interdisciplinarity* and *transdisciplinarity* are often used interchangeably, and these approaches are fundamental in areas such as planetary health and sustainability or astrobiology (Parro et al., 2020). The concept of transdisciplinarity, however, is more frequently used when there are elements of qualitatively different disciplines such as the sciences and social sciences (as in astrobioethics, for example: Chon‐Torres, 2021). Work that is inter‐ or transdisciplinary can benefit climate‐change studies (Serrao‐Neumann et al., 2021; Weart, 2013) and studies of human health and health interventions including some of those carried out according to the ethos of the ‘One Medicine’ or ‘One Health’ concept (e.g., Kusters et al., 2020; Schwabe, 1964).

Box 4 **Creativity in scientific research.**

In his treatise on the creative process *The Hidden Order of Art* (Ehrenzweig, 1970), Anton Ehrenzweig explains that the pursuit of creativity/novelty generally requires a holistic vision made by connecting disparate pieces of concrete/objective information (Supplementary Text ‘The Child's Vision of the World’) and, conversely, observations or data that enter the mind can trigger syncretistic understanding and creative insight (Supplementary Text ‘Training Spontaneity Through the Intellect’). The same modes‐of‐perception are needed in science, and also when transferring knowledge or phenomena to other systems/situations. As Ehrenzweig explains: ‘The study of…the scanning process in science [and of art's unconscious substructure] offers the needed opportunity for observing…creative techniques…and the way in which it makes use of the dispersed structure of unconscious perception. The chaos of the unconscious is as deceptive as the chaos of outer reality [and] we need the less differentiated techniques of unconscious vision to become aware of their hidden order [in other words, conscious thought tends to artificially fragment objective reality]. The scientist has to face [this] fragmentation of physical reality…’ (Ehrenzweig, 1970; for a full quotation, see Supplementary Text ‘The Child's Vision of the World’).

Whereas perhaps obvious to many, it is important to realise that scientific novelty depends in large part on the creative process, which in turn frequently requires the attention of the unconscious mind. In this way, creativity is an inherent product of the human mind and human experience. Another perhaps obvious (yet important) is that human creativity is not something that emerges automatically from within the datasets produced by practical experiments. This said, rational scientific thought and intuition are not in any way juxtaposed to experimentalism: scientific progress as a whole ultimately depends on both practical experiments *and* thought (Figure 1) as discussed by author William I.B. Beveridge in his book *The Art of Scientific Investigation* (Beveridge, 1950), even if these two elements do not always coincide within each individual study.

At our current stage of modern science, we have an overabundance of scientific data. Indeed, across the life sciences, we have more information than we can currently understand (see Human creativity and use of ‘creative’ computational technologies below) and, in general, scientific thought is required to make sense of it. This is well‐illustrated by some of the studies that analyse data derived from genomic sequencing to answer unexplored scientific questions (e.g., Hug et al., 2016 and Gao & Wu, 2022a that are discussed below).

Another example is provided by the early work of Margulis (formerly Lynn Sagan), a geneticist who elucidated the evolutionary origin of the eukaryote cell in relation to microbial symbionts that came to function as intracellular organelles (Margulis, 1970; Sagan, 1967). Whereas this finding was in part made using evidence and ideas of others (as discussed by Dorion Sagan, the son of Lynn Margulis, in Sagan, 2021), the tenacious and meticulous approach of Margulis drove her to produce perhaps the most‐convincing treatise on this topic: ‘On the origin of mitosing cells’ (Sagan, 1967). This paper can be considered a landmark of the endosymbiotic theory, yet it was only published in the *Journal of Theoretical Biology* after many rejections from other journals. The molecular demonstration that the eukaryotic organelles mitochondria and chloroplasts are descendants of bacterial cells confirmed that her endosymbiogenesis theory was correct (Sagan, 1967). She later went on to defend cooperation (rather than competition) between species as the most‐important evolutionary driver (Margulis, 1998), consistent with the insights into the Pareto Principle from Timmis et al. (2023). Margulis's finding had far‐reaching effects, not least to show that evolution does not only occur via gradual Darwinian‐type changes arising from natural selection and (for some species) from sexual selection. As her son put it: ‘Margulis's influence on 20th Century biology is…breathtaking and owed [to her] familiarity with live microbes. In an age of increasing scientific specialisation the need for synthesis becomes greater’ (Sagan, 2021). We consider Margulis a natural philosopher, and being interested in the importance of gases as metabolic substrates and products of microbes, she met Lovelock with whom she began a productive collaboration not least in relation to the Gaia hypothesis of Lovelock and Margulis that is discussed in Urgent global challenges below.

In some cases, more practical experiments are needed to interpret biological aspects of empirical data that we already have; in other cases, computational techniques can be utilised (see below); and in yet other cases, theoretical, thought‐based research that utilises natural philosophy approaches is required. A famous example of the latter—in relation to the human system—is the classic book *The Concept of Mind* (Ryle, 1949) which is about René Descartes's concept of mind and body that gave rise to the erroneous notion of the mind existing *within* the body. Gilbert Ryle dubbed this dualistic concept ‘the ghost in the 2022 machine’. His book sought to rectify this flawed paradigm (that he explains is a logical category mistake) by examining how mental processes and physical processes are not two independent things. The book's introduction explains that: ‘The…arguments which constitute this book are intended not to increase what we know about minds, but to rectify the logical geography of the knowledge which we already possess’ (for the full quotation, see Supplementary Text ‘The Concept of Mind’). Ryle himself was a philosopher, and practical experimentation involves some elements of philosophy: formulating a hypothesis or question, interpreting data to discover the scientific finding(s), and reaching conclusions, predictions, and speculation about the future (Figure 1; Beveridge, 1950; Brüssow, 2022). As Maureen A. O'Malley recently observed in an email to J.E.H. ‘Everyone – microbiologists included – is doing something philosophical in his or her science, but it's not always recognised or dealt with explicitly’.

Some paradoxes that represent a potentially fallacious perception of reality (or an aspect of reality) can remain in place for decades or even centuries just because they were simply always perceived that way; quite often, people do not challenge what is familiar or accepted. Once again, language (including scientific terminology) tends to perpetuate our scientific thinking and worldview; such as in relation to space and time (see also Inconsistencies in language and logic as a trigger for scientific investigation below). One study that illustrates this is: ‘Concepts of the last eukaryotic common ancestor’ (LECA) (O'Malley et al., 2019). Like the origin of life and the last universal common ancestor (LUCA), the issue of LECA has long been a contentious and active area of research inquiry (Margulis et al., 2006; Weiss et al., 2016).

O'Malley et al. (2019) did not examine a particular phylogenetic reconstruction of early eukaryotic evolution, but instead examined the very nature of LECA and whether it could be understood as a single cell, an abstract phylogenetic state, a pan‐genomic population (a single population that is genetically heterogenous), or a consortium of organisms. The team was made up of a philosopher (O'Malley herself) and three (perhaps philosophically‐minded) evolutionary microbiologists. Following through with the conceptual/theoretical issues in relation to the scientific understanding of LECA, the authors found that LECA should be thought of as a pan‐genomic population rather than—as is the most‐commonly held view—a single cell. The study by O'Malley et al. discusses the implications for ecology, geography, fitness, and diversification of LECA, and it examines the implications for phylogenetic reconstructions of early eukaryote evolution and thereby provides what they describe as a ‘conceptual toolkit for developing theories of LECA and interpreting genomic datasets’ (O'Malley et al., 2019).

The ‘pan‐genomic population’ scenario had not been considered previously for LECA, so the 2019 study challenged standard views of what an ancestor is and how to conceptualise the origins of a major new lineage of life. Although yet to be universally accepted (Gabaldón, 2021), there is also a widespread acceptance that LECA was a pan‐genomic population as evidenced by the majority of the 26 citations that O'Malley et al. (2019) have thus‐far received (December, 2022).

The story of identifying and characterising an ecologically and phenotypically distinct group of organisms, microbial weeds, is another example of a finding that emerged from a natural philosophy approach: ‘The biology of habitat dominance; can microbes behave as weeds?’ (Cray, Bell, et al., 2013). Microbial weed species are those that systematically dominate the communities in open habitats of microbes. As for the ecology of plant weeds, open habitats are key to understanding the ecology of microbial weeds, but the respective definitions of microbial weeds versus plant weeds are subtly different (see Supplementary Text ‘Microbial weeds versus plant and animal weeds’; Table 1 of Cray, Bell, et al., 2013).

Apart from describing microbial weed species, a second outcome of this study was to define and identify open habitats of microbes: habitats that are resource‐rich and non‐extreme yet at least partially vacant (Cray, Bell, et al., 2013). A third outcome was to subtly refine the definition of plant weeds (see table 1 of Cray, Bell, et al., 2013), and a fourth outcome was to classify the antimicrobial substances deployed by some weed species based on their modes‐of‐action. The latter are volatile organic compounds that act as either hydrophobic or highly chaotropic stressors; biosurfactants; organic acids; moderately chaotropic solutes that are produced in bulk quantities, such as acetone and ethanol; and toxins (that have a target‐specific mode‐of‐action). This classification gave rise to further work relating to cellular stresses imposed by the metabolic products of biofuel‐producing microbes, including a decision tree to identify modes‐of‐action of inhibitory substances (Figure 1 of Cray, Stevenson, et al., 2015). This classification of antimicrobials has also formed the basis of other subsequent studies (e.g., Alves et al., 2015; Heinz et al., 2021; Noel et al., 2023; Yakimov et al., 2015).

The inception of the Cray, Bell, et al. (2013) study came about during the mid‐1990 s when J.E.H. noticed that some microbes are metabolically wired to systematically dominate their communities under specific environmental conditions. He had studied plant weed biology as an undergraduate in Applied Plant Sciences, with the author of the book *Plant Weed Biology* (Hill, 1977) as one of his lecturers: Thomas A. Hill (Wye College, University of London, England). The natural philosophy aspects of the Cray, Bell, et al. (2013) study were that it dealt with the topic holistically and at multiple levels (from microbial behaviour to biophysical properties of the environment; from temporal dynamics of population growth and other aspects of ecology to identifying the phenotypic traits and characterising underlying physiology, metabolism, biochemistry, and/or genetic traits) and utilised scientific approaches to deal with concrete biological evidence using an approach largely based on logic or philosophy. It perhaps seems remarkable that the concept of a weed species (studied intensively in plants already over the past 100 years – see Supplementary Text ‘Microbial weeds versus plant and animal weeds’) was not identified in microorganisms until this relatively recent study.

It would be impossible to do a single experiment to obtain data to test the hypothesis that microbes can behave as weeds; indeed, it could plausibly consume a 40‐ or 50‐year research career to properly achieve the aims of the Cray, Bell, et al. (2013) study using practical experimentation. Even if this experimental work was carried out, the data would not be novel in as much as these data in effect existed already in the published literature. Given this situation—that is similar to that faced by Pedrós‐Alió (2021) (see Box 2)—J.E.H. and co‐authors instead used extant data and their own observations, and in this way evaluated the evidence for weed behaviour, weed cellular and metabolic traits, and weed ecology of diverse types of microorganisms. Using 375 referenced papers, the authors re‐evaluated data and thereby defined open habitats of microbes; showed microbial habitat dominance in action; identified about 10 phenotypic traits that can facilitate dominance; and where possible identified the metabolisms, metabolites, proteins and genes that enable these phenotypic traits. Whereas microbial weed species do tend to be highly competitive, it should also be noted that cooperative interactions can also facilitate dominance in some cases (Timmis et al., 2023).

Cray, Bell, et al. (2013) can be seen as a new paradigm in microbial ecology because neither open habitats of microbes nor their weed biology had been identified previously. There had been occasional uses of the expressions ‘bacterial weeds’, ‘fungal weeds’, or ‘laboratory weeds’ in earlier papers (e.g., Eilers et al., 2000; Porter, 1931; Zengler et al., 2005) but they referred to a microbial contaminant on a Petri plate or in culture broth (a usage of ‘weed’ that parallels the way in which the term ‘plant weeds’ is used colloquially to describe unwanted plants growing in any specific location). In addition to the implications of weed ecology for natural ecosystems, many—if not most—of the microbial biotechnology sector arises from the weed traits (e.g., antimicrobials such as ethanol) of a small number of microbial weed species (Cray, Bell, et al., 2013; Cray, Stevenson, et al., 2015). Open‐habitat ecology of microbes has many implications in the context of food spoilage, wound microbiology, depletion of the microbiome and creation of open habitats by antibiotics, and community dynamics in natural habitats (Cray, Bell, et al., 2013). The 2013 microbial weed study has been cited about 240 times (Google Scholar; December, 2022) and used to inspire/interpret practical experimental work (e.g., Deroo et al., 2022; McLellan & Roguet, 2019) as well as thought experiments. For example, some months after the publication of Cray, Bell, et al. (2013), Aharon Oren (The Hebrew University of Jerusalem, Israel) contacted J.E.H., and suggested a thought experiment to understand the ecology of the halophilic archaeon *Haloferax mediterranei* because it appeared to have weed‐like traits yet was rarely found as an abundant or dominant taxon in brines (Oren & Hallsworth, 2014). It transpired that its weed‐like traits do allow dominance within some sediment communities (Lee et al., 2018); a finding consistent with the original microbial‐weed study which stated that ‘For weed species such as *Saccharomyces cerevisiae* (high‐sugar environments), *Haloquadratum walsbyi* (aerobic, hypersaline brines), and *Gonyostomum semen* and *Microcystis aeruginosa* (eutrophic freshwater) ability to dominate can be restricted to a specific type of habitat [or set of conditions]’ (Cray, Bell, et al., 2013).

Other studies that emerged from natural philosophy approaches include ‘Keystone taxa as drivers of microbiome structure and functioning’ (Banerjee et al., 2018) that focused on an ecological class of microbes based on their behaviour (as described in Supplementary Text ‘Towards an understanding of keystone microbes’) and ‘Marine microbial diversity: can it be determined?’ (Pedrós‐Alió, 2006), as described in Supplementary Text ‘Diversity and ecology of marine microbes’. In the context of microbial ecosystems, important advances are also being made (via both theory‐based and empirical approaches) based on the analysis of the biology of individual cells (Wood, 2022).

### Inconsistencies in language and logic as a trigger for scientific investigation

In the pursuit of lucid science and novel scientific findings, we suggest that analysing terminology and language can often provide a fertile basis for progressing science in both theory‐based studies and those arising from practical experimentation. It is easy to assume in science that progress can only be made by inventing and applying new laboratory techniques and simply generating more data rather than analysing and understanding whatever information we already have. This is akin to palaeontology where there is a tendency to search for ever more samples rather than analyse the vast reservoir of material already stored in museums that can be a rich source of information for studies of DNA, morphology, taxonomy, and use of computational techniques such as augmented reality (Bimber et al., 2002; Yang et al., 1997) or genomics and metagenomics where there is a drive to sample more and more places and obtain more and more sequences rather than analyse and make biological sense of those we already have.

There is much written about the limitations that language confers on thinking, including impacts on scientific progress in both practical experimental studies and those studies carried out beyond the practical experiment (Crombie, 1995; Holyoak & Morrison, 2005). The fact that artificial intelligence struggles with language attests to the major role that language plays in human thought processes (Hutson, 2021). Words are of course profoundly useful as they augment human memory; facilitate communication, bonding, and social complexity; and enable us to document and archive information, not least scientific information. However, language can in equal measure be deleterious because it conditions, restricts, and channels our thinking. Importantly, language also attributes logical categories to the subject that is expressed (Crombie, 1995; Holyoak & Morrison, 2005) so can act to propagate and perpetuate inaccurate or illogical ideas and interpretations. These can even persist across human generations so language can impede scientific lucidity and progress, as demonstrated by the examples of Ryle (1949) and McKay (2004). This phenomenon can occur regardless of which language is being used. Language affects thinking often in unnoticed and subtle ways that nevertheless are important because they can determine the nature and outcome(s) of the thought process (Gleitman & Papafragou, 2005). Famously, Einstein once said **‘**I very rarely think in words at all. A thought comes, and I may try to express in words afterwards’ (Calaprice, 2010).

Let us take the concept of *life* (McKay, 2004), which is a somewhat spurious linguistic construct that has caused—and continues to cause—much‐confused thinking. In the context of biology, cellular and organismal systems do of course *exist*. The biochemical, metabolic, and other life processes that organisms exhibit do of course *occur*. However, ‘life’ is a linguistic construct that is in every sense an artefact because life *per se* does not *exist*. Therefore, this noun—as useful as it can be—lacks meaning in the context of either logic or biology. Nevertheless, we seem to be somewhat stuck with such terms. In science, however, the ultimate challenge is to limit the ways in which language limits us.

The constraints and erroneous thinking that can arise from inconsistencies in language and logic can give rise to mistakes in relation to the logical geography of microbiology. One example is the confusion between the concepts of cellular ‘stress’ and cellular ‘toxicity’ (see Supplementary Text ‘Cellular stress and toxicity are conceptually and mechanistically distinct’) combined with the fact that water‐mediated cellular stress was (and often still is) assumed by microbiologists to be synonymous with—and confined to—osmotic stress.

Many studies have been carried out towards understanding the mechanism of ethanol‐induced inhibition of cellular systems, especially in *Saccharomyces cerevisiae*. However, ethanol has often been (erroneously) regarded primarily as a toxic substance (Lei et al., 2016; Mota et al., 2021; Scopes, 1989; van Uden, 1985). This assumption might have caused a lack of clarity for many decades about the precise mode‐of‐action of ethanol as a stressor, or how to mitigate against this cellular stress during industrial fermentations. Whilst working on *S. cerevisiae* (in 1994), J.E.H. noticed that this substance, widely referred to as ‘toxic’, was actually damaging the cell at multiple diverse sites (targeting all types of macromolecular systems), at different levels of biological complexity, and at higher concentrations than a toxic substance. Therefore, he reappraised the impacts of ethanol on the cell, and the cell's responses to these impacts: ‘Ethanol‐induced water stress in yeast’ (Hallsworth, 1998). Whereas this review described the way in which ethanol entropically disorders biomacromolecular structures (known as chaotropicity), he was not at that time familiar with the term ‘chaotropicity’. Nevertheless, it was perhaps the first description of chaotrope‐induced stress and stressresponse *in vivo* (for any type of organism).

This 1998 study re‐assessed terminology by re‐assigning ethanol from presumed toxin to being, in reality, a stressor (an issue explored in more detail in later studies; Cray, Stevenson, et al., 2015; Hallsworth, 2018; Hallsworth et al., 2003; Noel et al., 2023); and by challenging the assumption that water‐mediated cellular stresses are necessarily caused by osmotically active substances. J.E.H. stated that ‘The effect [of ethanol] on water‐availability was quantified several decades ago (Hodgman, 1944; Perry, 1963), but the pathological significance of ethanol‐induced water stress has rarely been recognised’ (Hallsworth, 1998). Conversely, many reviews have been written on water stress and polyol metabolism in yeasts (e.g., Blomberg & Adler, 1992; Brown, 1990; Onishi, 1989) but ethanol was not identified as a source of water stress. To some extent, the use of osmo‐terminology may have obscured this mode‐of‐action. Osmo‐terminology implies that water stress only occurs in circumstances where there is a movement of water across a membrane. This suggests that water stress does not operate [via a chaotropic mode‐of‐action] at an enzyme and lipid–lipid bonding level. Furthermore, osmo‐terminology implies that reduced water availability is necessarily caused by high concentrations of dissolved solids. It [therefore seemed] unlikely that a water‐miscible liquid, and a solvent in its own right, would act as a potent source of water stress’. The study of J.E.H. was rejected by a number of journals because it was perceived as controversial at the time it was first submitted in 1995, so took a further 3 years to get published. Nevertheless, it led to other works by J.E.H. during the decades that followed (most of which are based on practical experimentation), so to some degree provided the basis for his career that followed (see Supplementary Text ‘Work that followed from a theory‐based study of ethanol stress’).^4^

In the concluding remarks of their LECA study, O'Malley and colleagues also point to scientific confusion caused by linguistics and the misallocation of logical categories: ‘although it can be convenient to talk about LECA as a single cell, this may mislead researchers who interpret shorthand expressions at face value. Genealogical conceptions of LECA that trace back to a single cell should not be conflated with tracing genomic ancestry, for which the source will be a population. Taking a more complex view of LECA, such as a population with a pan‐genomic structure, will complicate already demanding considerations in the phylogeny of eukaryotes, but may also inform more sophisticated reconstructive efforts. At the very least, broader concepts of LECA – including the novel pan‐genomic view we outline above – will influence how those reconstructions are interpreted biologically, ecologically, and evolutionarily’ (O'Malley et al., 2019).

Whereas it is not a microbiology study, the early paper about artificial intelligence ‘Computing machinery and intelligence’ (Turing, 1950) is pertinent to the current manuscript (see Human creativity and use of ‘creative’ computational technologies below) and is a classic example of making scientific progress via the analysis and/or deconstruction of language. Turing was able to devise what has come to be known as the Turing Test by thinking deeply about how words like ‘machine’ and ‘think’ can be misused colloquially. This has spawned a large and productive research agenda in diverse areas including information and computing sciences, psychology, and cognitive science (for more details, see Supplementary Text ‘Can machines think?’)

Another example of disharmony between linguistics, logic, and science is the misuse of the term ‘chaotropicity’ that resulted in a realignment of its presumed mechanistic meaning and caused confusion that has spanned a period of half a century. Chaotropes—such as ethanol, urea, MgCl_2_, phenol, and hydrophobes—entropically disorder biomacromolecular structures and their interactions (Bhaganna et al., 2010; Cray, Russell, et al., 2013; Cray, Stevenson, et al., 2015; Hallsworth et al., 2003; Hamaguchi & Geiduschek, 1962). The ‘chao’ (entropic) effect of these substances was first described in a study where some ions were found to disorder the structure of DNA (Hamaguchi & Geiduschek, 1962). However, the term was misused and misunderstood (initially by physical chemists), during which time it was mistakenly assumed that the concept described substances that in some way disorder the hydrogen‐bonded network of water molecules (even in a pure solution of chaotrope and water), rather than relating to the entropic state of biomacromolecules. In recent years, physical chemistry data show that these substances do not disorder water, so the concept of chaotropicity is frequently said to be redundant. This misassignment of the mechanism, and the resulting confusion, are discussed by Ball and Hallsworth (2015). A further, astrobiological example of language‐related confusion has implications for planetary protection and relates to the nature of a planet's surface (Hallsworth, 2021; see Supplementary Text ‘The surface of Mars’).

## WHEN THEORY IS THE MOST‐APPROPRIATE ROUTE TO SCIENTIFIC NOVELTY

The most‐scientifically robust and most‐direct route to address a scientific question or hypothesis is not always via practical experimentation (Figures 1 and 2). Therefore, we believe in taking a case‐by‐case (situational–functional^5^) approach, by considering all available options prior to deciding on the ideal research approach. For some scientific questions, practical experiments are essential, but for others, experiments can be deleterious or inviable (e.g., Casadevall, 2005; Cray, Bell, et al., 2013; Gao & Wu, 2022a; Hug et al., 2016; Levinthal, 1969; McKay, 2004; Pedrós‐Alió, 2021; Price, 2009; Timmis & Hallsworth, 2022). By way of example, as stated in the context of water as a preservative of microbes: ‘It would be difficult to mathematically model, or experimentally test, the hypothesis that water is a preservative of cellular life; for example, what could be used as a meaningful control?’ (Hallsworth, 2022).

In some cases, thought experiments lead to practical studies (e.g., Partida‐Martínez & Heil, 2011)—and vice versa—but in some other cases, hybrid (practical experiment + theory‐based) approaches are needed, such as the study described by Mills et al. (2022) in artificial intelligence in the search for novelty beyond the experiment below and various studies by the authors of the current article (e.g., Benison et al., 1998, 2021; Brancini et al., 2022; Cray, Bell, et al., 2013; Cray, Houghton, et al., 2015; Hallsworth & Nomura, 1999; Hamill et al., 2020; Johnston et al., 2022; La Cono et al., 2020; Lloyd et al., 2020; Malki et al., 2008; Micheluz et al., 2022; Pinkerton et al., 2021; Schmidt et al., 2021; Stevenson et al., 2015). Further examples are a recent study by Klawonn et al. (2021) ‘Characterizing the ‘fungal shunt’: Parasitic fungi on diatoms affect carbon flow and bacterial communities in aquatic microbial food webs’; a report by Lünsdorf et al. (2000) about interactions between bacterial cells, clay leaflets, and hydrophobic substrates ‘Clay hutches': a novel interaction between bacteria and clay minerals'; and a 2017 study by Jay T. Lennon (Indiana University, IN, USA) and colleagues who took a hybrid approach to solve the microbiological conundrum described below.

Lennon et al. (2017) published a thought experiment, augmented by a practical experiment, relating to methane‐oxidising bacteria within caves: ‘Microbial contributions to subterranean methane sinks’. We contacted Lennon, who explained that this study was inspired by an earlier paper that had claimed that sub‐atmospheric methane concentrations were due to abiotic oxidation caused by radiolysis (Fernandez‐Cortes et al., 2015) whereby nuclides produced deep in the Earth were entering the cave and making contact with methane molecules. While different mechanisms were plausible for explaining the natural phenomenon, this one could likely be ruled out by carefully taking into consideration assumptions and biophysical constraints.

So, Lennon et al. (2017) performed a thought experiment to arrive at a better first‐order approximation for the feasibility of radiolysis to reduce cave methane concentrations. The approach provided a theoretically grounded argument that radiolysis was insufficient to explain the field‐based observations in the caves. The authors followed up with practical experiments which demonstrated that rates of microbial methane oxidation in caves are in fact high and could reduce concentrations of gaseous methane (Lennon et al., 2017). In a later paper, Lennon and colleagues collaborated with two authors of the Fernandez‐Cortes et al. (2015) paper to experimentally test whether radiation can oxidise methane at rates sufficient to support the original suggestion by Fernandez‐Cortes et al. (Schimmelmann et al., 2018). The Lennon et al. (2017) study was useful for understanding the contributions of microorganisms to the overlooked sink of greenhouse gases that may be important for local and regional climate‐change modelling. The field of systems biology further exemplifies the value of hybrid theory‐based + practical approaches (Liu et al., 2022; Westerhoff, 2011), and the study of halophile ecophysiology study by one of us, M.M.Y., and his colleagues (La Cono et al., 2020); see below.

### Use of genomic datasets

The freedom to think freely and test hypotheses using genome‐sequence data can give rise to qualitatively new and important scientific findings relating to microbial ecology, evolution, and cell physiology. An example is ‘A new view of the tree of life’ by Hug et al. (2016), one of the first papers to appear in what was at that time a new journal, *Nature Microbiology*. This work by Hug and colleagues organised microbial diversity based on genome sequences including, for the first time, many lineages for which we do not have cultured representatives. Their study was theory‐based because sufficient genome data were already publicly available and, whereas we cannot replicate evolution over that has occurred over a timescale of 3 to 4 billion years, we can model it using sophisticated phylogenetic tree inference. This phylogenetic tree is a resource that not only organises data, but unifies nomenclature, and generates new hypotheses; see Aouad et al. (2018) about long‐branch attraction artefacts, Moody et al. (2022) about core‐gene phylogenies based on ribosomal versus non‐ribosomal genes, and Parks et al. (2022) about definitive genome‐based taxonomy. Having all of these lineages in one place and shown visually as a tree emphasised the import and potential of these data, and the need for systems to handle new genomes with greater rigour. The advent of long‐read sequencing has increased the numbers of complete, closed bacterial genomes for uncultivated lineages, accentuating the need for new systems of data organisation and aiding the development of a more‐complete tree of life (Albertsen, 2023). Figure 1 of Hug et al. (2016) revealed the weight of evolutionary divergence in the *Bacteria* in a way that is both irrefutable and spectacular. It has sparked conversations about microbial evolution and taxonomy that continue to have significant reverberations through the scientific community in relation to new nomenclature systems and new proposals for naming these uncultivated groups (Aouad et al., 2019; Feng et al., 2021; Murray et al., 2020; Sorokin et al., 2017, 2019).

We asked Laura A. Hug (University of Waterloo, Canada) about this ‘tree‐of‐life’ study. From a technical standpoint, she considers the work to be a ‘resource’ paper or, from a narrative standpoint, a ‘showcase’ paper because it reveals/displays the breadth of life's diversity and highlights how much of it has only now become accessible to us thanks to new sequencing methods. It seemed that new phyla were being named every month or two, but there was no comprehensive view of how these different lineages related to each other. Hug and colleagues needed a tree that encapsulated the current knowledge of microbial diversity to put their own new lineages into a wider context.

The phylogenetic tree (Figure 1 of Hug et al., 2016) is now the Wikipedia image for the ‘Tree of life (Biology)’ entry, and has been incorporated into undergraduate degree courses and textbooks (Henkin & Peters, 2020; Wessner et al., 2020). The article has also considerable reach beyond this, with an Altmetric score for the online attention of 2069 (December, 2022) that is ranked 1st out of 58 tracked *Nature Microbiology* papers of a similar age. It has thus far been cited 1670 times (Google Scholar; December, 2022), and has also captured the public interest. The Hug et al. study featured in numerous news articles has been used in anthologies of data visualisation (including *The Little Book of Data* 2022), and the tree‐of‐life image (Figure 1 of Hug et al., 2016) is also part of a museum exhibit ‘The Beauty of Early Life. Traces of Early Life’ (at the ZKM|Center for Art and Media Karlsruhe, in cooperation with the State Museum of Natural History Karlsruhe, Germany). The work of Hug et al. (2016) has also inspired/impacted many subsequent studies, including those relating to the bacterial radiation known as Candidate Phyla Radiation identified by research in Jillian F. Banfield's laboratory (University of California, Berkley, CA, USA; Brown et al., 2015; Wrighton et al., 2012). These relate to the reclassification of the Candidate Phyla Radiation as the Patescibacteria (Parks et al., 2018) and the use of a hybrid (theory‐based + antibody‐based) approach to isolate specific Patescibacteria from complex communities within the human microbiome as pure cultures (Cross et al., 2019). In another hybrid study conducted with a DPANN nanohaloarchaeon and its haloarchaeon host/partner, the application of a theory‐based approach (the genome‐inferred putative hydrolytic capacity supported by cultivation data) revealed a hitherto unidentified symbiosis (La Cono et al., 2020) as described in Supplementary Text ‘Candidate Phyla Radiation’.

Novel scientific findings also emerged via the analysis of genomic datasets in the study: ‘Microbial genomic trait evolution is dominated by frequent and rare pulsed evolution’ (Gao & Wu, 2022a). The authors of this study analysed more than 10,616 bacterial genomes and 263 archaeal genomes to determine whether the microbial trait evolution is similar to that of eukaryotes in terms of tempo and mode, whether pulsed evolution occurs across the tree of life and, if it has done, then to what extent it contributed to microbial trait evolution (Gao & Wu, 2022a). One of the most‐exciting findings from this study is the detection of two distinct types of pulses: small frequent jumps and large rare jumps that had been predicted previously by Eldredge and Gould's punctuated equilibrium theory (Eldredge & Gould, 1972) and Simpson's quantum evolution theory (Simpson, 1944).

Gao and Wu (2022a) hypothesised that small frequent jumps are associated with speciation while large rare jumps are associated with the origination of higher taxa: genus, family, order, etc. Martin Wu (University of Virginia, VA, USA) explained to us that the reason why they had expected their study might detect two types of jumps (while previous studies only detected one type) is that the bacterial phylogeny in the Gao and Wu (2022a) study spanned a wide range of macroevolutionary timescales: the shortest branch on the tree represents 0.1 million years of evolution while all branches together represent about 0.9 trillion years of evolution. An implication of this study is that the origins of major bacterial and archaeal lineages might happen in quick bursts instead of through the slow divergence of species over prolonged periods of time.

Wu explained that the authors stumbled onto this topic when trying to study ecological patterns of microbial communities using 16S rRNA sequencing reads. There are two vexing problems with using the 16S rRNA gene for microbial diversity studies. First, the gene‐copy number of 16S rRNA in the genome varies from species to species (anywhere from 1 to 16 copies) and this large variation can bias the relative species abundance estimated using 16S rRNA read counts. Second, the 16S rRNA gene‐copy number for the vast majority of bacterial species is unknown. To deal with these problems, researchers (Gao and Wu included) have developed methods to predict the copy number of 16S rRNA gene in *Bacteria* using an approach known as hidden‐state prediction, which is rooted in ancestral state reconstruction. To do that, one needs to have a decent trait‐evolution model to determine the 16S rRNA copy‐number variation. Gao and Wu (2022a) started with the commonly used Brownian motion model, under which the distribution of phylogenetically independent contrast should be normal. However, they observed a strong leptokurtic pattern (a distribution with values concentrated around the mean), suggesting that extremely large trait changes (large ‘jumps’) happened more frequently than expected under the Brownian motion model. This reminded the authors of the patterns predicted by Eldredge and Gould's punctuated equilibrium theory. A literature search by Gao and Wu revealed that Uyeda et al. (2011) and Landis et al. (2012) had developed mathematical models that they had used to demonstrate the presence of pulsed evolution in the body‐size evolution of vertebrates. Gao and Wu realised that little has been done in studying the tempo‐ and mode‐of‐macroevolution in microbes, which motivated their Gao and Wu (2022a) study.

This research led Gao and Wu to develop a new method for ancestral state reconstruction and hidden‐state prediction based on the pulsed‐evolution model (Gao & Wu, 2022b). When applied to empirical trait data, their method outperformed the commonly used ancestral reconstruction methods and significantly improved the confidence level of the predictions (Gao & Wu, 2022b). The authors also developed a new tool named RasperGade16S for better predicting the 16S rRNA gene‐copy number (Gao & Wu, 2022c), which is why the authors commenced this line of study in the first place. It may be that the Gao and Wu (2022a) paper will change people's perception of the tempo and mode‐of‐evolution across the tree of life because pulsed evolution appears to be a recurring motif for each domain of life rather than some kind of idiosyncratic exception.

Other studies have used analyses of genome‐sequence data to decipher novel aspects of microbial metabolism, including ‘Glycerol metabolism of haloarchaea’ (Williams et al., 2017). Glycerol is an important stress protectant (compatible solute) that is also used for osmotic adjustment in many eukaryotic microbes (Brown, 1990), little studied in bacteria (Bhaganna et al., 2010, 2016), and virtually unheard of in *Archaea*. However, *Archaea* are known to use glycerol as a nutrient source. Whereas a substantial proportion of *Archaea* is thought to be uncultivatable, there is a considerable amount of publicly available genome‐sequence data. Williams and colleagues decided to use this information and, if available, they also used previously published data on growth phenotypes to determine the presence of glycerol catabolic pathways in haloarchaea and deduce aspects of the roles cell physiology and ecology.

Some halophilic prokaryotes accumulate ions for osmotic adjustment, but others use organic compatible solutes or a combination of ions and organic compatible solutes. Of the organic compatible solutes (glycerol, proline, betaine, erythritol, ectoine, arabitol, mannitol, trehalose, etc.), only glycerol is known to be a sufficiently soluble and low‐molecular‐mass solute to reduce intracellular water activity to the levels required for cellular function in most naturally occurring brines, including those dominated by NaCl (Alves et al., 2015; Lee et al., 2018). For extremely salt‐tolerant haloarchaea, glycerol is the most‐likely contender for an organic‐compatible solute. However, Williams and colleagues considered this unlikely due to the permeability of archaeal membranes to glycerol. Instead, they focused on glycerol catabolism (though the membranes of fungi and algae that utilise glycerol for osmotic adjustment are also permeable to this polyol; see Alves et al., 2015 and references therein).

Williams et al. (2017) studied 32 closed haloarchaeal genomes to identify genes involved in glycerol catabolism including those involved in the conversion of glycerol to dihydroxyacetone phosphate (DHAP), and the uptake of glycerol or glycerol‐3‐phosphate. They then carried out phylogenetic analyses to assess the evolutionary relationships of specific genes including the role of horizontal gene transfer in conferring specific traits (Williams et al., 2017). The authors then integrated findings from these genomic analyses with published physiological and ecological data to determine the role of glycerol in haloarchaeal growth. They found that 27 out of the 32 haloarchaea appeared to be able to catabolise glycerol according to the presence of key enzymes within their genomes and that the glycerol‐3‐phosphate pathway is more prevalent in these haloarchaea than the dihydroxyacetone pathway. In addition, they elucidated various aspects of glycerol physiology/ecophysiology and proposed that glycerol‐3‐phosphate might be linked to motility in some species. The authors, for example, observed that the inability to catabolise glycerol (for *Halanaeroarchaeum sulfurireducens*, *Halovivax ruber*, *Natronobacterium gregoryi*, *Natronobacterium pharaonic*, and *Halobacterium* sp. DL1) correlates with the habitats in which they are found (Williams et al., 2017). These taxa generally occur in anoxic environments and are only able to grow using elemental sulphur as the electron acceptor and acetate and pyruvate as carbon substrates. *Natronobacterium gregoryi* and *Nmn. pharaonic* inhabit alkaline soda lakes where the alga *Dunaliella* (a primary source of glycerol for *Archaea* living in pH‐neutral brines) is absent, so they do not need to catabolise glycerol. Whereas there is no direct evidence that any haloarchaea utilise glycerol as a stress protectant (whether taken in from the environment or synthesised de novo), the authors of the current article believe that this remains an open question.

### Insights relating to thermodynamic parameters

Another article that took a scientifically direct approach to solve a research question relates to the thermodynamic parameter *water activity*: ‘Water activity in Venus's uninhabitable clouds and other planetary atmospheres’ (Hallsworth, Koop, et al., 2021). The inspiration for this study was a *Nature Astronomy* paper about Venus's atmosphere (Greaves et al., 2021) that put forward putative evidence of Earth‐type microorganisms in Venus's clouds. Greaves et al. (2021) reported that phosphine concentrations in Venus's atmosphere were so high that this gas may have been produced biogenically and authors of Greaves et al. published further papers around the same time to provide more details on the microbiology of Venus's clouds (Bains et al., 2021; Seager et al., 2021). The Greaves et al. (2021) study was published early online in September 2020 and at this time featured widely in the world's news media because, if true, this would be the first life to have been found beyond Earth.

Venus's clouds are thought to consist primarily of sulphuric acid (Young, 1973), a substance known to greatly reduce water activity that has been long‐used for this property in experimental protocols (Wilson, 1921). Water activity is the ratio between the water‐vapour pressures of the solution and pure water under the same temperature and pressure conditions (ranging from 1 down to 0), and the lowest water activity at which microbes have been observed to divide/grow is 0.585 (Stevenson et al., 2017). It was suspected by J.E.H. that microbial function in Venus's clouds is implausible, not least because their water activity was likely far below this value so he assembled a team of collaborators to prepare a Letter to the Editor of *Nature Astronomy* that disputed the notion that active microbial ecology could take place in the Venus clouds (Hallsworth, Dallas, et al., 2021).

Within 1 or 2 days of starting to write, it became clear that the datasets generated, and the number and complexity of the displays produced, would not fit into a short‐form Letter format. Moreover, the reader would need a Methods section to explain the research approach that was developed to ascertain the water‐activity range of Venus's clouds. Therefore, the ‘letter’ morphed into a full‐length research article (Hallsworth, Koop, et al., 2021). Novel calculations were needed to compute the relative humidity of Venus's atmosphere from an altitude of 40 to 70 km; that is, the domain where the temperature range is consistent with the temperature range for an active life on Earth: about +130 to −40°C. These calculations were made based on direct measurements from Venus Express (a European Space Agency orbiter) and Venera missions (Soviet Union probes). The relative humidity of the atmosphere determines the water activity of the cloud droplets; these are known to have a mean diameter of 1 μm, so they equilibrate with the atmosphere within seconds (Hallsworth, Koop, et al., 2021). Conversion of water‐activity values to sulphuric acid concentrations required the use of the Extended Aerosol Inorganics Model (E‐AIM) of Clegg et al. (1998), which is discussed below.

The Hallsworth, Koop, et al. (2021) study found that the water activity of Venus's clouds was virtually zero (0.00003 to 0.0037), which is two orders‐of‐magnitude below the requirements of active life on Earth, a finding that was independent of the actual composition of the cloud droplets. This point is important given that the composition of the clouds is uncertain (Rimmer et al., 2021; Shao et al., 2020; Zhang et al., 2012). Furthermore, if the clouds are primarily sulphuric acid, their acidity is three orders‐of‐magnitude above that tolerated by the most acid‐tolerant microbe known on Earth. Indeed, based on the known water‐activity range, the sulphuric acid concentrations would be from 77.8% to 99.2% w/w; values at which organic substances are turned to elemental carbon (Hallsworth, Koop, et al., 2021). Again, this would confirm that the Venus clouds are inconsistent with Earth‐type life, a finding consistent with that of Lovelock and Giffin (1969).

The Hallsworth, Koop, et al. (2021) study appeared in a 2021 issue of *Nature Astronomy* alongside that of Greaves et al. (2021). Whereas the former did not validate the claim of microbial life on Venus, it did pioneer a new methodology to determine the habitability of planetary atmospheres based on their water activity–temperature combinations and in the context of known limits for Earth life. It also provided insight into which parts of Earth's atmosphere can permit microbial function, confirmed that the Mars atmosphere is too cold to be habitable, and unexpectedly revealed that Jupiter has a water activity–temperature combination that would be permissive for the biotic activity of some forms of microbial life (Hallsworth, Koop, et al., 2021). Maybe more importantly, it provided a means by which we can determine the habitability of atmospheres of planets beyond our Solar System (Figure 2), for example, based on data from the James Webb Space Telescope that sent back the first measurements of exoplanet atmospheres during the preparation of the current article, in July 2022.

The Hallsworth, Koop, et al. (2021) study, carried out during the COVID‐19 pandemic, reached audiences beyond academia in as much as it featured in 1076 news articles (according to media‐monitoring company Vuelio, UK). It is also featured on a Wikipedia page of the significant events in science in 2021 (https://en.wikipedia.org/?curid=69206295) and received an Altmetric score for the online attention of 2752 (December, 2022) that is ranked 2nd out of 72 tracked *Nature Astronomy* papers of a similar age. It was the availability of E‐AIM that was developed more than 20 years earlier by Simon L. Clegg and colleagues that facilitated the Hallsworth, Koop, et al. work: ‘Thermodynamic model of the system H^+^–NH_4_^+^–SO_4_^2−^–NO_3_^−^–H_2_O at tropospheric temperatures’ (Clegg et al., 1998).

This Clegg et al. study was carried out to develop a model to compute key parameters of H^+^ − NH_4_^+^ − SO_4_^2−^−NO_3_^−^−H_2_O systems at tropospheric temperatures. Their 1998 model, an extension of several previous models that Clegg and his co‐workers had developed (Carslaw et al., 1994, 1995), was devised to ‘represent aqueous phase activities, equilibrium partial pressures (of H_2_O, HNO_3_, and NH_3_), and saturation with respect to solid phases (H_2_SO_4_ and HNO_3_ hydrates, (NH_4_)_2_SO_4(cr)_, (NH_4_)_3_H(SO_4_)_2(cr)_, NH_4_HSO_4(cr)_, (NH_4_)_2_SO_4_·2NH_4_NO_3(cr)_, (NH_4_)_2_SO_4_·3NH_4_NO_3(cr)_, and NH_4_HSO_4_·NH_4_NO_3(cr)_) in the system H^+^−NH_4_^+^−SO_4_^2‐^−NO_3_^‐^−H_2_O’ from 328 to <200 K, i.e. from 55 to less than −73°C, (Clegg et al., 1998). Clegg and colleagues started with water/sulphuric acid, then added nitric acid and HCl and HBr, and finally ammonia. An earlier breakthrough with this work was made when Carslaw et al. (1994, 1995) extended the model by nitric acid; they were then able to predict a new type of polar stratospheric clouds which turned out to be important and relevant as shown by subsequent field observations and laboratory experiments. Whereas Clegg et al. (1998) was not a study of microbiology, it is pertinent to determinations of habitability for planetary atmospheres given that active microbial metabolism, growth, and ecology occur in Earth's atmosphere (Archer et al., 2021; Hallsworth, Koop, et al., 2021).

### Theory‐based development of techniques for practical experimentation or fieldwork

In some cases, theoretical studies have proved to be a productive route to develop new methodologies, including approaches and techniques needed for fieldwork or practical experimentation. Two examples of methodological development, via a meta‐analysis of existing datasets in the study ‘Meta‐analysis of quantification methods shows that the *Archaea* and *Bacteria* have similar abundances in the subseafloor’ by Lloyd et al. (2013) and a new mode of studying subsurface microbes in the article ‘Sampling across large‐scale geological gradients to study geosphere‐biosphere interactions’ (Giovannelli et al., 2022), are highlighted in Box 5.

Box 5 **Theory‐based approaches to method development in the Karen G. Lloyd research group.**

A long‐standing problem in environmental microbiology is the accurate quantification of specific microbial groups in environmental samples. However, one of us (K.G.L.) compared outcomes across many published samples and found that a pattern emerges suggesting new techniques that would improve the accuracy of such methods: ‘Meta‐analysis of quantification methods shows that archaea and bacteria have similar abundances in the subseafloor’ (Lloyd et al., 2013). This study arose due to delays in the availability of a laboratory facility upon starting a new job, which prevented the start of practical experiments. Therefore, K.G.L. instead chose to analyse data from published papers: to determine average copy numbers for the 16S rRNA genes are used as taxonomic markers of *Archaea* and *Bacteria*. However, she was unable to answer the question because the data that were presumed to be absolutely quantitative were either not quantitative or only semi‐quantitative. This finding could not be ascertained from any individual study and only emerged after comparing data obtained from many published studies. It has since allowed others in the research area to use these methods as relative quantitative measures or as imaging techniques (albeit that they are not quantitative in an absolute sense).

K.G.L. was also involved in the theory‐based development of techniques for fieldwork. Traditionally, deep‐subsurface microbial ecology has relied on discrete samples from just one or two sites, but approaching such studies from a broader spatial scale, similar to that used for geological studies, can give insights that would be missed by smaller, more‐focused approaches. In their study ‘Sampling across large‐scale geological gradients to study geosphere‐biosphere interactions’ (Giovannelli et al., 2022), K.G.L. and colleagues were inspired by geological studies where data are collected over a large spatial scale to infer functions occurring over timescales that would be too long to sample during a human lifespan. Their theory is that a subsurface landscape can be developed by collecting excurrent waters from natural springs across a broad geological context, based on the relationship between the microbes that are flushed out in the seeps and the tectonically‐driven geochemical environments (Giovannelli et al., 2022). This represents a new methodological approach to the study of the deep‐subsurface biosphere that is likely to provide new insights that cannot be provided by traditional microbiological analyses of an individual hot‐spring pool, or even by comparing several of these (without the deeper geological context).

Another example relates to the development of pulsed‐field gel electrophoresis by Schwartz and Cantor (1984). Conventional electrophoresis uses a single electrical field to cause biomolecules to migrate through a matrix according to its mass‐to‐charge ratio; the migration distance of the biomolecule is indicative of its mass or size (Klotz & Zimm, 1972). This conventional technique can effectively separate DNA fragments up to ~20 kb, but larger fragments will co‐migrate and appear as a large band at the top of the gel when imaged. Several years after the Klotz and Zimm study, Schwartz and Cantor (1984) utilised a thought experiment to overcome this problem by inventing pulsed‐field gel electrophoresis (PFGE) to then perform their study ‘Separation of yeast chromosome‐sized DNAs by pulsed field gradient gel electrophoresis’. Pulsed‐field gel electrophoresis resolves DNA mixtures by alternating the electrical field between spatially distinct pairs of electrodes. This technique results in the separation of DNA fragments of up to ~10 Mb based on their reorientation and movement at different speeds through the pores of an agarose gel. Here, we give an overview of pulsed‐field gel electrophoresis and the factors to be considered during sample preparation.

The history is that David C. Schwartz was a graduate student in Boston who came up with the idea to separate chromosomes by thinking how motorcycles and small cars can out‐pace trucks in the Paris–Dakar Rally due to their small size. This is because trucks have to slow down at each turn to correct the inertia and avoid overturning. He envisioned that DNA molecules of different sizes could be separated in an electrophoresis field that pulses in different (perpendicular) directions every few seconds because the smaller DNA molecules will recover motion in the new direction faster than the larger DNA molecules. On this basis, he reasoned, it should be possible to separate chromosomes. His tutor in Boston did not pay attention to this idea, so Schwartz moved to Columbia University where Professor Charles R. Cantor allowed him to test this concept on the bench. This took him several years but, finally, he showed that it was possible to—for the first time—separate chromosomes of *S. cerevisiae*. This invention was patented, the Schwartz and Cantor (1984) paper was published, and then the era of genomics began. One of us (R.A.) was there at this time (whilst doing his postdoctoral research) as a witness and still has one of the original pulse‐field machines designed by Schwartz. The most‐difficult part of the project was to obtain naked chromosomes without breaking them into smaller pieces, but Schwartz was able to achieve this by immobilising cells on agarose for the removal of cell components (a process that has since been termed ‘cellular striptease’). Hence, this thought experiment was converted into a new and important laboratory technique (cited more than 2000 times according to the publisher's website, December 2022). The study by Schwartz and Cantor (1984) demonstrates how lateral thinking can lead to a step change; in this case, via a new technique.

Some of the other articles highlighted in the current study are also theory‐based studies that have produced new methodologies, including Clegg et al. (1998) that developed a model for application in diverse types of research; McKay (2004) that proposed a new methodology to find microbial life in other planets; the studies by Price (2009) and Price and Sowers (2004) that determined limits of microbial longevity and cellular function at subzero temperatures; Partida‐Martínez and Heil (2011) that recommended scientifically robust experimental designs; Cray, Bell, et al. (2013) and Banerjee et al. (2018) that can be seen as providing conceptual and methodological frameworks on which practical experiments in microbial ecology can be based; Hallsworth, Koop, et al. (2021) to determine habitability of planetary atmospheres; Pedrós‐Alió (2006) that provided a conceptual map for types of microbial diversity studies; Das et al. (2021) who designed a new deep‐learning algorithm; and O'Malley et al. (2019) who proposed methodologies for phylogenetic reconstructions of early eukaryote evolution. In addition, a study by Cray, Houghton, et al. (2015)—‘A simple inhibition coefficient for quantifying potency of biocontrol agents against plant‐pathogenic fungi’—produced a new methodology to analyse interactions between competing microbes based on the colony geometry of microbial co‐cultures over time (see Supplementary Text ‘Quantification of competitive interactions’).

## HUMAN CREATIVITY AND USE OF ‘CREATIVE’ COMPUTATIONAL TECHNOLOGIES

Given that thought runs throughout the scientific process, novel findings can emerge from intuition, imagination, and speculation. Creative insight typically arises from, or utilises, some kind of observation (or measurements/data acquisition) and methodology/technique whether in the sciences, the humanities, or the arts (Box 4; Ehrenzweig, 1970; Goldstein, 2022; Koestler, 1964). Important drivers of scientific breakthroughs can be the discovery of oddities or peculiar biological events, but scientific breakthroughs can also occur via the discovery of unexpected multivariate correlations.

The latter typically come from the systematic analysis of large datasets, sometimes too large to be assimilated readily by the human mind. More data are produced today than at any other point in the history of science (Gauthier et al., 2019), and this is particularly clear in fields such as ‘omics, healthcare, and bioinformatics where our ability to store the vast volumes of the sequence data generated, and our ability to find biological meaning within these data, lag far behind our capacity to merely produce the sequencing data (Cremin et al., 2022; Prakash & Taylor, 2012). The inordinate scale of the available datasets can render the task of searching for unusual biological phenomena/events prohibitively difficult without the aid of technology (Greener et al., 2022). Increased accessibility and storage of scientific data, coupled with increased computing power, have made computational techniques such as artificial intelligence (Russell & Norvig, 2020) more affordable for research purposes.

### Artificial intelligence in the search for novelty beyond the experiment

For some research topics, computational technologies can be used to extend the reach of human creativity (Boden, 1998; Colton & Wiggins, 2012). Artificial intelligence is a subdivision of computer science that attempts to simulate human capabilities, mainly to perform tasks related to decision‐making and problem‐solving (see Supplementary Text ‘Can machines think?’). The artificial intelligence approaches most commonly used to analyse scientific data are machine learning (Greener et al., 2022; Murphy & Bach, 2012) and deep learning (Goodfellow et al., 2016); see Box 6. The purpose of machine‐learning algorithms is to make computers *learn* to recognise patterns from data in order to accurately predict outcomes without the need for direct programming (Box 6).

Box 6 **Training of artificial intelligence algorithms.**

Machine learning is a subset of artificial intelligence that uses algorithms and statistical models to analyse, and draw inferences, from patterns within the data provided. The younger discipline 'deep learning' (a subset of machine learning) uses layers of input‐ and output data, called ‘artificial neural networks’; the latter can progressively extract and classify and analyse features within the data. Both methodologies differ in the type of input data they accept and in the methods they use to learn from the data; see the Box 6 figure below (created using BioRender.com).

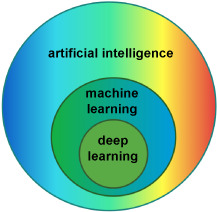

Machine‐learning and deep‐learning algorithms are constrained in their scope by the data being fed into the algorithm and these algorithms cannot be compared with the flexibility, dexterity, and adaptive capacity of the human mind. Nevertheless, there are some deep‐learning algorithms capable of learning to overcome existing biases in the training datasets provided so are considered to be excellent learners. In general, artificial intelligence algorithms *learn* from sample data sets that are fed to the algorithm to ‘train’ them to recognise specific patterns. A validation stage is then initiated (though this is not always necessary), using a subset of the sample dataset to evaluate the algorithm's performance and optimise/tune the algorithm and ensure that it is learning the instructions required to make accurate predictions or find specific patterns using the data. After this process, which can be repeated until the algorithm reaches a satisfactory level of accuracy, it is fed a test dataset (never before provided to the algorithm) to evaluate algorithm performance post‐training. The data given to the algorithm during the training process have considerable influence on final performance and accuracy (see Limitations and potential interdisciplinary use of artificial intelligence).

One question is: can we teach artificial intelligence algorithms to be creative? Some novel deep‐learning multilayer‐network algorithms have already demonstrated that there could be a place for them in the creative scientific process (Anantrasirichai & Bull, 2022, Lehman et al., 2016). Combined with our understanding of cognitive psychology, these techniques have opened up the possibility of extending the reach of the creative process in humans with some degree of artificial (computational) creativity (Boden, 1998; Colton & Wiggins, 2012). Indeed, there are now many artificial systems that have been designed to produce artworks such as music and painting (Baraniuk, 2017; Hitsuwari et al., 2022; Hutson, 2017; Ramesh et al., 2022), though it is still debatable whether this is akin to human creativity.

The utilisation of artificial intelligence for hypothesis generation (step 2 in Figure 1) typically makes use of two types of computational creativity: combinatorial creativity (the product of the unexpected combination of prior knowledge), and exploratory creativity (the product of the exploration of the conceptual space) (Boden, 1998). In this vein, artificial intelligence frameworks in their current form could be used for hypothesis formulation in an iterative manner, with the first iteration represented in steps 2 to 4 of Figure 1 of the scientific process. At these three stages, artificial intelligence algorithms would be fed vast datasets in an exploratory manner and/or combine and integrate these data. The latter would then be used by the researchers to produce a new, refined hypothesis.

An application of this scenario is the study ‘Accelerated antimicrobial discovery via deep generative models and molecular dynamics simulations’ (Das et al., 2021) in which the authors designed an artificial intelligence computational framework that follows a two‐step approach using deep‐learning classifiers augmented with high‐throughput physics‐driven molecular simulations to accelerate the discovery of new antimicrobial peptides. This strategy reduced the time required to design a *de‐novo* therapeutic molecule, from years to just ~48 days. The role of the first deep‐learning algorithm applied by the authors was to screen the full range of peptides provided to capture key information about their function and constituent molecules to be able to design other molecules (beyond the peptide sequences provided) that have similar characteristics. The ‘creative’ step of this computational pipeline was performed by a system developed by the authors, called controlled latent attribute space sampling (CLaSS), which collected the data generated by the first algorithm and produced a set of new molecules each of which has bespoke characteristics. To produce these antimicrobial peptides, the authors first generated a pool of 90,000 peptide sequences using CLaSS and based on properties of the > 1.7 million short‐peptide sequences in the UniProt database. Finally, this pool of artificial intelligence‐generated peptide sequences was screened using deep‐learning classifiers for additional key attributes, such as toxicity and broad‐spectrum activity. This pool of peptide sequences was then further reduced to a set of 20 that passed the ‘simulated’ screening step and became the new research objects of the study. Wet‐laboratory validation of the 20 artificial intelligence‐designed candidates then led to the discovery of two low‐toxicity peptides with strong antimicrobial activity against a diverse group of pathogens (Das et al., 2021).

The use of deep‐generative models such as that presented by Das et al. could become a key component of strategies to counter the threat of antimicrobial resistance, especially the emergence of multidrug‐resistant pathogens. Their article has received considerable online attention, with 27,000 accesses since its March 2021 (early online) publication in *Nature Biomedical Engineering* and has been cited 112 times already (Google Scholar; December, 2022). The manuscript is ranked by Altmetric as having the 4th highest score out of 38 tracked articles of a similar age in the journal—a score of 161—and has already been featured in a number of specialist reviews (e.g., Greener et al., 2022; Melo et al., 2021).

At the hypothesis‐generation stage, artificial intelligence frameworks can also help in a more‐theoretical way (Extance, 2018). For example, the role of natural language processing techniques is to enable machines to process and understand human language (Scaccia & Scott, 2021). Today, natural language‐processing applications that are widely used include Apple's Personal Assistant Siri (CA, USA) and Amazon's Alexa (WA, USA), as well as the predictive text features in search engines and mobile phones. However, natural language‐processing techniques can also be applied to extract, summarise, and analyse massive text datasets. Thus, the increasing availability of fully open‐access articles in machine‐readable formats now provides for the possibility of using natural language‐processing tools to generate novel methodological frameworks that could be applied to extract targeted data from scientific articles, which would help reduce the workload and speed up the literature‐review process. The target data extracted from the literature in the screening process could be used to build more specific databases with their own trends, correlations, and analyses, thus supporting more‐effective discovery approaches (Olivetti et al., 2020). Moreover, applying natural language processing to scientific literature can also be useful to predict future research trends by analysing co‐occurrences of selected keywords over time (see step 8 in Figure 1).

The application of artificial intelligence techniques in later stages of the scientific process, such as data analysis (step 5 in Figure 1), has also accelerated the pace and quality of downstream analyses. Techniques such as dimensionality reduction, which works by transforming the data to reduce the number of input variables or features in a dataset, are now key components of microbiome studies. This is because these techniques allow researchers to interpret high‐dimensional data from complex and dynamic systems, such as microbial communities, without losing key relationships between different samples in the analysis (Armstrong et al., 2022; Martino et al., 2021). Other techniques, such as machine‐learning feature extractors and classifiers, utilise machine‐learning models to extract common features from disparate datasets and make predictions based on the variation of these features between samples. Such methodological approaches have been employed to predict states (healthy/disease) using microbiome compositions (LaPierre et al., 2019), and for biomarker discovery and quantitative assessment of microbiome‐phenotype associations (Pasolli et al., 2016).^6^

Deep‐learning techniques applied to biological data are in their infancy, yet they do look promising. State‐of‐the‐art tools such as those employed by Das et al. (2021), involving generative artificial intelligence systems and hybrid cloud technologies could significantly accelerate the throughput of complex scientific pipelines aimed at the discovery of new types of drugs, as well as materials, and fertilisers. In addition to speeding up the process of screening and selecting the most‐promising drug candidates to treat infections such as COVID‐19 (Stokes et al., 2020), machine‐learning techniques could be used to assist physicians, for example, by analysing computed tomography (CT) scans for lung lesions and formulating a diagnosis (Zhang et al., 2020), and even for predicting medical conditions by analysing symptoms—such as coughing—using a smartphone application (Imran et al., 2020). The implementation of machine‐learning algorithms in the COVID‐19 pandemic has also been an example of how machine learning could be applied to help overcome future challenges that might arise such as the emergence of new human pathogens that cause future pandemics.

The ability of machine‐learning algorithms to deal with the increasing amount of digital data available has in turn enabled more‐comprehensive solutions to research problems. The integration of different layers of biological information from ‘omics techniques, such as metabolomics, proteomics, transcriptomics, epigenomics, genomics and metagenomics—termed ‘multi‐omics integration’—provides a more‐holistic understanding of the complex flow of information that simultaneously occurs, at different levels in biological systems (e.g., the single cell and the microbial community; Pinu et al., 2019). Due to the difficulty imposed by the combination of high‐dimensional heterogeneous data from different datasets, machine‐learning analysis of multi‐omics data is still at an early stage (Reel et al., 2021). However, this novel approach has already been employed successfully in several recent studies. For example, it has been used to shed light on the different dynamic immune responses of hospitalised patients with stable or progressive COVID‐19 infections (Unterman et al., 2022) and to improve the understanding of the functional alterations observed in the microbiota of patients with single inflammatory bowel disease that drive ulcerative colitis (Mills et al., 2022).

In this recent study, ‘Multi‐omics analyses of the ulcerative colitis gut microbiome link *Bacteroides vulgatus* proteases with disease severity’, Mills and colleagues used data obtained from several ‘omics approaches (such as faecal meta‐proteomics, metabolomics, 16S rRNA gene‐amplicon sequencing, shotgun metagenomic sequencing, meta‐peptidomics, and serum proteomics), integrated these using artificial intelligence, and then carried out in‐vitro and in‐vivo validations, which revealed that certain taxa within the microbiota, such as *Bacteroides vulgatus*, aggravate ulcerative colitis due to their elevated protease activity (Mills et al., 2022). The work was, therefore, a hybrid study where artificial intelligence approaches were used alongside wet‐biology experiments. The Mills et al. study is an example of how integrative ‘omics studies can be used to develop novel scientific hypotheses via a bottom‐up approach in which the exploratory analysis of data leads to a meaningful result. Using machine‐learning techniques, Mills and colleagues correlated the clinical metadata (such as disease symptoms) obtained from hundreds of patients with the data obtained from the metagenomic and meta‐proteomic experiments; their analysis was then progressively refined according to the observations produced by the integrative analysis of the other multi‐omics datasets such as serum proteomics and metabolomic. The authors then refined and validated their hypothesis via wet‐laboratory experimental work. Thus, hybrid (artificial intelligence + multi‐omics) studies not only provide a novel approach and possibly a more‐realistic investigation of biological systems but also demonstrate the potential of artificial intelligence algorithms for creating new directions of biological investigation. As a consequence, deep‐learning algorithms are replacing more‐traditional modelling components in holistic studies of biological phenomena, such as in systems biology research, where it is crucial to understand multi‐scale biological processes occurring simultaneously in the living organism.

### Limitations and potential interdisciplinary uses of computational technologies

Considerable developments have been made in the artificial intelligence field in recent decades (see Supplementary Text ‘Early developments in artificial intelligence’; LeCun et al., 2015) but artificial intelligence techniques are limited by the same factors that have made them so successful today. These include the availability of computing power and the storage of copious volumes of data and their subsequent availability for public use. Moreover, artificial intelligence models are limited by the ability of the programmer to define or implement complex concepts into computer code and by the capacity of the user to train and implement these algorithms. As long as they are used/applied within the scope defined by their parameters/training, artificial intelligence algorithms (or any other algorithms) cannot in essence be unsuccessful in as much as they simply perform the tasks that they are programmed for.

Machine learning‐based artificial intelligence algorithms are data‐driven and therefore have some limitations based on the availability of quality training data to generate adequate predictive models. This is due to the use of faulty or poorly‐curated datasets that impair the performance of these algorithms. Thus, the process of extracting biological information from the published literature or publicly‐available unstructured data and their integration into a training database is a time‐consuming ‘human’ step that requires not only computational skills but also expertise in the biological area in question (Vamathevan et al., 2019). In addition, datasets obtained in different studies (from practical experiments that use different techniques and/or different experimental designs) cannot be compared or analysed together if the data are not sufficiently normalised and homogenised. It should also be noted that most of the predictions made by artificial intelligence frameworks on biological data are unverified such that wet‐laboratory experiments are needed to confirm them.

The large language model/chatbot ChatGPT was released very recently, in November 2022, after the submission of the current article. Nevertheless, ChatGPT has already been used to write scientific papers and has, in some cases, even been listed as an author (Else, 2023; Stokel‐Walker, 2023). As Ball (2023) observes, this type of large language model can in some ways have value: ‘People who struggle with literacy skills are already using ChatGPT to improve their letters of job application or their email correspondence. Some scientists are even using it to burnish their papers before submission. Arguably these artificial intelligence technologies can democratize language [and] for those with limited English‐language skills who need to write professionally, the technology could be a great leveller’. This said, publishers are now moving to ban the use of large language models in the context of paper authorship on the basis that they are unethical, cannot take scholastic or legal responsibility for authorship, and that their use can even be regarded as plaigarism (Anon., 2023; Thorp, 2023). As Ball (2023) also states, such technologies have no notion of empathy or communication, let alone thought: ‘We have little experience in dealing with an resource that so powerfully mimics thought while possessing none…[futhermore], the algorithm of a large language model does not have a communicative goal—it has no notion at all of what communication is, or of having an audience…And it is hard to see how a language model could ever truly innovate, for…it is designed…to ape, mimic, and, as a statistician would put it, regress to the mean, which tends toward the mind‐numbingly drab’. In the current article, we highlight the intrinsic value of human engagement in the scientific process and do not believe that this engagement should be or can be displaced by artificial intelligence technologies.

Another important issue is the lack of transparency with which some of the artificial intelligence systems operate, also known as the black‐box model, where the processes and operations performed by the system are not revealed to the user and thus make it difficult to understand and explain the predictions or decisions that the artificial intelligence system makes. Therefore, there are still certain stages in the scientific process such as the identification of the scientific findings and the formulation of conclusions that cannot be done by artificial intelligence (in particular step 1 and steps 6 to 10 in Figure 1). There may also be a problem in reproducing the results of the analyses carried out with these algorithms if they are not properly archived in a publicly accessible repository.

Artificial intelligence is already impacting many other scientific disciplines and research fields—such as mathematics, materials science, physics, geoscience—as well as economics and finance (Ghoddusi et al., 2019; Kusters et al., 2020; Xu et al., 2021), and it could also help to address other, more‐complex scenarios and challenges such as global warming and climate change (Kadow et al., 2020; Nishant et al., 2020). These kinds of challenges ideally require interdisciplinary (or at least multidisciplinary) approaches in which artificial intelligence could serve as a common instrument for researchers from different scientific areas that would use their knowledge about natural systems to propose novel algorithms and models and to interpret the dynamics of complex systems. In this regard, research with an interdisciplinary flavour such as complex networks‐based techniques and statistical physics, that heavily relies on the laws of probability and statistics of multiple interacting components, have been applied in recent years to substantially advance our understanding of the Earth System. An example is the development of integrated tools that use deep‐learning forecast models to predict generic early‐warning signals leading to tipping points of complex dynamical systems in which external conditions that change slowly cause sudden shifts to new and, in some cases, dramatically different states (Bury et al., 2021). These algorithms provide early warning signals that are applicable to a wide range of systems from different disciplines such as ecology, thermoacoustics, climatology, epidemiology, and systems biology.

In the context of Earth‐system science, planetary, geochemical, and biological processes interact with each other across all spatial and temporal scales, and deep‐learning algorithms can help to predict dynamic properties using long‐range spatial connections across multiple timescales. The data used to carry out these predictions can come from different sources including, for example, geostationary satellites, *in‐situ* observations from autonomous sensors in the subsurface, and studies of ecology, organismal physiology, or evolutionary biology. These datasets can be integrated using a multivariate modelling approach but also more recently, using hybrid modelling techniques that combine deep‐learning algorithms with traditional physical modelling (Reichstein et al., 2019). This kind of interdisciplinary approach can generate meaningful interpretations of the outcomes, including mitigations to prevent the collapse of dynamic systems that are approaching tipping points in relation to climate change (see Box 3, and Urgent global challenges below).

Whereas artificial intelligence algorithms will always operate within constraints, within these boundaries they can do things that the human mind cannot do. For instance, they can solve some problems that involve processing enormous volumes of data in seconds in a consistent and robust manner. Findings from the use of artificial intelligence approaches can also provoke the formation of new hypotheses or otherwise spark human creativity and the integration of these approaches into key steps of the scientific process could give birth to a new form of synergistic creativity between humans and computers, which could improve and accelerate the rate of scientific discovery in relation to some research topics. Furthermore, the challenges and hurdles that we encounter using these technologies provide incidental insights into the nature of the scientific process, not least in relation to the role of language for example (see above). Artificial intelligence is, therefore, potentially synergistic with human creativity in multifarious ways.

Artificial intelligence is already widely used in research, for instance, algorithms are involved in making principal component analysis (PCA) plots and software used for genome sequencing, analysing proteomics data, annotating a genome, and performing phylogenetic reconstructions. In relation to human creativity versus computational ‘creativity’, we believe that this issue is one of semantics: to some extent creativity is creativity, and it arguably does not matter from which source scientific novelty originates. To summarise, we do not consider artificial intelligence or computational ‘creativity’ to be a substitute for human creativity or to be qualitatively equivalent to the human mind (Ehrenzweig, 1970; Lake et al., 2017). We also do not believe that computational tools should reduce the *joie de vivre* of full human involvement in the scientific process. It is nevertheless beyond doubt that the use of computational techniques has opened up new avenues for scientific research and provided different approaches to finding new scientific outcomes, for example by screening high‐throughput datasets.

## IMPLICATIONS AND PERSPECTIVES

Science beyond the experiment takes many diverse forms. These theory‐based studies not only allow scientific research to transcend limitations and barriers imposed by differences between disciplines; they can also act to counter excessive reductionism (Boxes 3 and 4), integrate biological phenomena/data across different levels of complexity or timescales (e.g., Casadevall, 2005; Cray, Bell, et al., 2013; Gao & Wu, 2022a; Hallsworth, Koop, et al., 2021; McKay, 2004; O'Malley et al., 2019; Price, 2009; Williams et al., 2017), and mitigate or circumvent other constraints that are imposed by methodologies used in practical experimentation (e.g., Ball, 2008; Clark, 1994; Das et al., 2021; Gao & Wu, 2022a; Hug et al., 2016; McKay, 2004; Mestre & Höfer, 2021; O'Malley et al., 2019; Price, 2009; Turing, 1950; West & Brown, 2005).

Very few practical experiments can integrate disparate datasets across levels of complexity, time, disciplines, etc. Furthermore, we believe that some maxims and paradigms that may be fallacious (or at least do not accurately describe the biological reality) can best be challenged, and the logical geography of the subject rearranged, using theory‐based research approaches (e.g., Arber, 1950; Casadevall, 2005; Cray, Bell, et al., 2013; Darwin, 1859; Gao & Wu, 2022a; Hallsworth, Koop, et al., 2021; Hug et al., 2016; McKay, 2004; Mestre & Höfer, 2021; O'Malley et al., 2019; Partida‐Martínez & Heil, 2011; Pedrós‐Alió, 2021; Sagan, 1967). In addition, the basic assumptions that are usually required to underpin practical experimental studies often come from theory‐based work (of course, the converse is also true: that data from practical experiments are required to further test and validate—or in some cases falsify—the findings or hypotheses of theory‐based studies).

Studies based on thought experiments, analyses based on the rearrangement of logical concepts, and other types of theory‐based analyses are often more disruptive or innovative than those based on practical experiments. In this way, therefore, some theory‐based studies catalyse progress within or across scientific fields more rapidly than those studies based on practical work. This can be especially true when the former is carried out by small scientific teams (Wu et al., 2019). In general, after a disruption of the *status quo* (such as that caused by a paradigm shift) large consortia of scientists, or numerous smaller (independent) groups, are needed to anchor and develop those theory‐based findings or ideas within the scientific canon; this often takes place via practical experimental/observational studies (Wu et al., 2019). As observed by Chu and Evans (2021), the accumulation of a large quantity of experimental research articles tends to—somewhat ironically—slow down scientific progress rather than speeding it up. This is because the majority of articles reporting practical experiments support the established canonical core of a discipline while limiting the chances of novel ideas catching the attention of the scientific community and shifting and improving the paradigm(s) within the field (Chu & Evans, 2021).

Innovative research is likely to be more‐widely read, used, and cited than ‘canonical’ research that conforms to established frameworks and thinking in relation to a given research topic/field (Foster et al., 2015). However, this potential reward of innovative (sometimes paradigm‐changing) studies does not necessarily offset the higher level of risk that arises (Foster et al., 2015). This can be especially troubling for innovative/disruptive studies that are theory‐based because, when their findings represent a step beyond the current cannon, they might be more difficult to publish given the (often unconscious) prejudice that can be directed towards some thought‐based and other types of theory‐based research that are discussed below (see also Box 7).

Box 7 **Neophobia need not suppress novelty.^7^**

Conservatism within the peer‐review process can play a valuable role in science by preventing scientific errors from arising. In some cases, however, conservatism can be associated with a lack of objectivity that can impede legitimate progress (Bedessem, 2021). It is to some extent human nature to feel a sense of security in what feels familiar or normal. However, this innate tendency can also create tensions (amongst colleagues and/or collaborators, or reviewers and editors) between the prevailing paradigms and new results that might change these paradigms or, in the case of interdisciplinary studies, between the disciplines (Andersen, 2013; Kuhn, 1959; Taylor & Barron, 1963). Of course, an irrational resistance to scientific progress can sometimes be accompanied by unhealthy feelings of competition that result in corrupt politics of reviewers or editors. For whatever reason, pioneers in research whose work is particularly novel are often viewed with suspicion.

Edgar Anderson writes in his book *Plants, Man and Life* (Anderson, 1952)^a^ about the American botanist Oakes Ames, a scholar with a visionary grasp of his field yet whose work was consistently misunderstood or ignored: ‘If a scientist is one jump ahead of his fellows in his thinking he is usually their acknowledged leader; if he is two jumps ahead he is thought to be eccentric and rather screwball but sometimes receives belated recognition in his old age. If he is three jumps ahead he is ignored, though posterity may eventually get around to appreciating his evidence as it did with Gregor Mendel’ (for the full quotation, see Supplementary Text ‘Oakes Ames [1874–1950]’). An analogous comment was made at a conference held in the memory of the physicist Erwin R.J.A. Schrödinger (*Schrödinger at 75: The Future of Biology* meeting—September 2018; Trinity College Dublin, Dublin, Ireland). At this meeting, Tomás J. Ryan introduced cognitive scientist and philosopher Daniel C. Dennett III who once worked with Gilbert Ryle, explaining that Dennett: ‘Unlike traditional philosophers…is a student of neuroscience, linguistics, artificial intelligence, and computer science, and psychology’ and is also cast in a mould that differs from most others. Dennett has also at times been viewed as a maverick (for the full quotation of Ryan, see Supplementary Text ‘Daniel C. Dennett III’).

Research work that is perhaps unconventional and non‐mainstream due to its novelty can be overlooked even when scientifically robust and irrefutable (for theory‐based and practical experimental studies alike). Resistance to non‐mainstream science can also arise from the mental effort or anguish of having to rearrange our thoughts to process and accept something new. This is most evident where scientific findings challenge paradigms and warrant structural changes in the reader's entire mental framework in relation to the topic. In such cases, journal editors and reviewers might even reject scientifically robust manuscripts that yield novel and important findings without realising that the actual problem with the manuscript was their own inability to incorporate the new science into their thought landscape. It may be in part for these reasons that papers that represent a step change, such as McKay (2004), have a considerable lag phase before becoming accepted and utilised. Other examples in the current manuscript include papers that went through a long series of rejections apparently due to their novelty (e.g., Sagan, 1967). Almost by definition, work that causes a paradigm shift is non‐mainstream and without receptivity to such studies, we would not have acknowledged Gaia (Lovelock & Margulis, 1974), natural selection (Darwin, 1839), endosymbiosis (Sagan, 1967), and some of the recent scientific developments highlighted in the current article. Furthermore, in each case we would not have the body of new science that subsequently flowed from these works.

It might be argued that for scientists working decades or centuries ago, the relative lack of advanced experimental techniques (compared with those available today) meant that thought experiments were more important. On the contrary, we believe the fact that earlier scientists were able to make extremely valid hypotheses, findings, and conclusions against a scientific backdrop of more‐elementary methods than we now have underscores the scientific potency of thought experiments and other kinds of theory‐based approaches even in relation to present‐day science. Modern laboratory techniques do not prevent us (but can sometimes obstruct us) from also making progress via thought‐/theory‐based approaches.

To some scientists, issues detailed in the current article may appear sufficiently obvious that they need not be said. But, to a considerable proportion of researchers, biological research remains an exclusively practical/experimental affair. One example of this is provided by the research‐assessment exercise (Research Evaluation Framework or REF) carried out for UK universities to determine the research quality of university departments. A key aspect of the REF assessment is the quality assessment/scoring of the four ‘best’ publications from each individual academic within a REF‐assessment (7‐year) period. In the first REF exercise (carried out in 2014), these outputs had to be research articles that typically report practical experiments. In the 2021 REF, for the first time, research reviews that represent major scientific advances were allowed. We believe that this enlightened policy is healthy for the scientific community and good for the advancement of science. We nevertheless note that some universities chose to disallow their staff from submitting reviews, including some of the most‐research active (Russell Group) universities. This self‐imposed policy indicates an apparent belief that novel science achieved through theory‐based studies will not be assessed favourably.

We believe that the policies of organisations that fund research as well as those of scientific journals ought to facilitate scientific progress by being open to theory‐based work (see Supplementary Text ‘Attitudes of journals and funding bodies’). It is noteworthy in this regard that some of the most‐eminent microbiologists have gaps in their publication records given the time (and risks) taken to make transformative steps that drive scientific progress (Larkin, 1999). In addition, metrics need to reward scientific advances equally regardless of the research approach employed. Finally, more value should be given to novel articles that yield scientifically major findings and might have paradigm‐shifting potential instead of low‐risk canonical works. This need is especially pressing given that research that is innovative/disruptive appears to be slowing down over time (Park et al., 2023). As illustrated by some of the studies highlighted in the current article, slowing down and having some extra time to think can yield positive consequences for scientific innovation and scientific quality. It may be that in this way, we can learn something from the COVID‐19 situation and might in the future consider generating extra time and space for theory‐based studies to complement practical experimental studies (and vice versa).

Time—and a desire—for reading more widely can also be key for carrying out highly innovative studies. As observed by Park et al. (2023): ‘…the decline [in disruptive research] represents a substantive shift in science and technology, one that reinforces concerns about slowing innovative activity. We attribute this trend in part to scientists'…reliance on a narrower set of existing knowledge. Even though philosophers of science may be correct that the growth of knowledge is an endogenous process—wherein accummulated understanding promotes future discovery and invention—engagement with a broad range of extant knowledge is necessary for that process to play out, a requirement that appears more difficult with time. Relying on narrower slices of knowledge benefits individual careers (Leahey, 2007), but not scientific progress more generally’.

### Receptivity to research beyond the mainstream

Neophobia can sometimes obstruct scientific progress, and this occurs most especially with science—and personality types—that are not mainstream (Box 7; Lubek & Apfelbaum, 1987). Nevertheless, scientific novelty can often arise when our concept of reality is challenged and sometimes even turned inside out (e.g., Ball, 2008; Hallsworth, 2022; O'Malley et al., 2019; Partida‐Martínez & Heil, 2011). In some cases, resistance to change arises where hitherto separate research communities or scientific disciplines use different rules and standards, operate within qualitatively distinct dogmas, and/or uphold different criteria for what is scientific thereby creating artificial barriers to the integration of knowledge (Andersen, 2013; MacLeod & Nagatsu, 2018). One example of this is provided by the different ways in which botanists and microbiologists classified blue‐green algae or cyanobacteria over recent decades.

Whereas the personality and thought processes of each person are unique, some personality types are more common than others. According to the 16 personality types of the Myers‐Briggs system that is based on Jung et al. (1971), the most‐common personality type is characterised by the combination of traits ‘introversion, sensing, feeling and judging’ (~14% of the population) and the rarest personality type is characterised by ‘introversion, intuition, feeling, and judgment’ (~1% to 3% of the population). People also differ in their personality/psychology according to their affinity for adaptation versus innovation, as described by the Kirton Adaption‐Innovation scale (Kirton, 1976). Whereas the value of these categorisation systems is sometimes debated (and other personality‐description systems exist such as that of Garcia et al., 2017), we believe that they can provide some meaningful insights into the mental functioning of people that is pertinent to the ways that scientists approach research problems. Moreover, there is a tendency for some scientists to be contemptuous towards other researchers/research that are/is very different from and perceived as inconsistent with their own personality style (regardless of how you assess personality or modes‐of‐thinking and perception). This is because, for them, it feels both unfamiliar and uncomfortable, and this can create unconscious prejudice.

In reality, researchers with qualitatively different personality types tend to work together productively; a well‐known example is that of Watson and Crick (1953). In the same way that journals should ideally facilitate diverse types of scientific approaches and outlooks (unless they specialise by necessity in only one type), we believe that it is healthy for scientific research to encourage diversity in the ways that people do science to counter the innate tendency of some to reject scientists/science that seem(s) unfamiliar to them (Parker, 2018). We do not doubt that such encouragement commonly takes place, but we are equally aware that prejudice against science and personality types that are non‐mainstream is also commonplace.

### A comment on education and epistemology

Students in biology‐related subjects ideally should also receive classes in epistemology (the branch of philosophy studying how knowledge is produced) to avoid the risk that microbiological research (for instance) is perceived as little more than a collection of laboratory techniques rather than creating or improving theories that explain the natural world (Sandoval, 2003). While some authors consider thought experiments as mere arguments (e.g., Norton, 2004a; Norton, 2004b), others recognise that this research approach as an important type of induction and a catalyst that is insightful, allows us to make judgements, and can produce scientific findings and conclusions not reachable by other means (Camilleri, 2014; Clatterbuck, 2013). Similarly, the capacity to explore the consequences of a theory in a particular situation (whether the latter is ‘real’ or imaginary) has been recognised as an important part of the epistemic goals of science (De Regt, 2009; De Regt & Dieks, 2005).

When lecturing to undergraduate or Master's students, J.E.H. asks theoretical questions during the lectures in relation to linguistic terms that represent basic biological concepts. In separate lectures, for example, he asks what ‘life’ is, what is the definition of the ‘cell’, what microbial ‘stress’ is, and whether the cell and the environment are two separate entities. Whereas the student cohort (numbering from 20 to 70 individuals) is typically interactive during these discussions, students are invariably unable to provide definitive answers. The concept of life has remained the subject of debate amongst experts over recent decades and centuries, so is not readily resolved. However, J.E.H. found that even Master's students (who already completed a first degree in a biological subject) were unable to identify basic characteristics of life in relation to entropy, for example (Schrödinger, 1944); or to distinguish between living structures versus life processes (see above). When asked what a cell is (a more‐straightforward question), typical responses include that it is the smallest biological unit of life but, depending on your point‐of‐view, this might be an enzyme, DNA, an organ, or an organism. In almost 20 years of posing this question, not one student has ever said that a cell is effectively a container; whereby an aqueous plasma is held within a phospholipid membrane. It is a revelation to biological sciences students when they become aware of their lack of familiarity with these basic concepts. Conversely, posing these questions to students has inspired publications by J.E.H. in relation to the continuum of the cytosol‐lipid bilayer‐extracellular solution (McCammick et al., 2010), the concept of stress (Hallsworth, 2018), and the nature of life (in preparation).

Two recent articles have also focused on ways in which microbiology can also be used to inspire the curiosity of children in the classroom: ‘The urgent need for microbial literacy in society’ (Timmis et al., 2019) and ‘Visualizing the invisible: class excursions to ignite children's enthusiasm for microbes’ (McGenity et al., 2020). These articles were followed up with ideas and initiatives to engage children to act as multiplicators in the information‐dissemination pathway (Timmis et al., 2020), and to inform them about the notions of stewardship and stakeholders (Anand et al., 2023; Timmis, 2023), in order that they come to appreciate the manifold ways that microbes can contribute to solutions to crises currently faces as well as those on the horizon.

Posing simple questions and suggesting thought experiments can even have value for preschool children. For example, a geology professor (K.C.B.) was asked by a local teacher to visit her preschool classroom of 3‐ to 4‐year‐olds because they had shown an interest in magnets. During this visit, one child asked where magnets come from. Dissatisfied with the answer that magnets are in some rocks, one child exclaimed ‘I think magnets come from the sky!’. K.C.B. then asked the children ‘How could we test that to see if it is true?’. They then elaborated a plan to spread a white sheet on the playground overnight and then see whether any magnets had appeared by the next morning.

Thus, the thought experiment then progressed to a practical experiment; a sheet was despatched to the playground surface and left there. By the following morning, no magnets had appeared, so the children decided to repeat the experiment for two more nights. After this, the class decided that magnets do not fall from the sky. K.C.B. brought a meteorite to class, showed how it was magnetic, and talked about how it was seen falling to Earth from the sky. Then the class talked about time and how maybe their experiment with the sheet in the playground could be improved by using both more sheets and longer time periods. It is clear that both theory‐based scientific thought and practical experiments are both fun and deeply intelligent for these 3‐ and 4‐year‐olds.

### Urgent global challenges

Microorganisms are implicated in many of the urgent challenges currently facing the world and are implicated in their solutions (Cavicchioli et al., 2019; Timmis et al., 2017, 2019). These include: global climate change (not least, the regulation of Earth's atmosphere composition), food security (the need for fertile soils, plant health, food preservation, and alternative protein sources), pollution biology (bioremediation of plastics, pesticide residues, hydrocarbons, and other xenobiotics), the need for sustainable energy (production of biofuels), organic waste‐processing (agricultural, forestry, domestic, and industrial wastes), ecosystem health and sustainability (oceans, freshwater, land, subsurface, etc.), and human health in relation to disease and biosecurity.

Interestingly, most of these challenges are related to a theory‐based concept which has not yet been demonstrated via practical experimentation; the Gaia hypothesis (Lovelock, 1972; Lovelock & Margulis, 1974) that is now recognised as a theory (Scheneider et al., 2004). ‘Gaia’ challenged the view of Earth as (more or less) a collection of rocks. It described a deep interaction between planetary domains—the biosphere, atmosphere, geosphere, and hydrosphere (including the cryosphere)—which maintains the environmental conditions for life. Lovelock and Margulis thereby described Earth behaving as a single regulatory system in which cooperation, integration, dynamism, and complexity were key factors that maintained a balance of the whole. This predicted, for example, that deforestation would be disastrous and has acted as a catalyst for the development of the Green Movement that champions environmental sustainability. The Lovelock (1972) and Lovelock and Margulis (1974) studies were at one level works of natural philosophy, but they were carried out based on empirical evidence by two scientists with a broad and deep knowledge of the natural world, including many years of observations of microbiology.

These important and now‐famous studies effectively prophesied the ecosystem‐ and biosphere failures that we now see taking place due to anthropogenic damage to the Earth's systems. They also appear to have encouraged atmospheric scientists, geologists, and biologists to think globally. While some biological mechanisms have been identified that contribute to stabilising the Earth, the biosphere has been catastrophically damaged during mass‐extinction episodes caused by periods of glaciation, volcanism, or other events (Hodgskiss et al., 2019; Kauffman & Walliser, 1990). Some of these events are now believed to have been caused by life itself (McGhee Jr., 2018). For example, cyanobacteria that appeared quite early on during the evolution of life on Earth carried out oxygenic photosynthesis that almost certainly caused climate change. This is because there was a large‐scale capture of CO_2_ (in the form of microbial biomass) and the oxygen produced oxidised methane, thus the concentrations of these two gases—the main greenhouse gases—were drastically reduced. As a consequence, Earth's surface temperature plummeted and likely was the cause of the first glacial (‘snowball‐Earth’) episode that took place ~2300 million‐years‐ago and caused a mass extinction (McGhee Jr., 2018). In addition, the oxygenation of Earth likely made some anaerobic life extinct, restricted anaerobes to anoxic environments, and selected for the evolution of facultatively aerobic organisms.

In relation to the current anthropogenic changes that are impacting planet Earth, the classic article ‘A safe operating space for humanity’ by Rockström et al. (2009) identified tipping points beyond which planetary health and sustainability are irreversibly damaged. The quantification of these changes and the prediction of these tipping points are essential in relation to the ecosystem services that nature provides to food security and other resources such as clean air and water. The detection of these thresholds is difficult using empirical data (Hillebrand et al., 2020), hence we should operate under a precautionary approach/according to the precautionary principle (De Smedt & Vos, 2022). Microorganisms are implicated in some of these, not least the large‐scale release of methane (a greenhouse gas significantly stronger than CO_2_) that is underway due to the climate change‐induced melting of permafrost: see ‘Climate change and the permafrost carbon feedback’ (Schuur et al., 2015) and ‘Microbiome assembly in thawing permafrost and its feedbacks to climate’ (Ernakovich et al., 2022). Recent articles by Kemp et al. (2022) and McKay et al. (2022) highlight the catastrophic consequences that climate change can have for humanity.

Two noteworthy papers from Timmis and colleagues were conceived to alert the general public, educators, policy‐makers, and other scientists to the key roles of microbes in global challenges: the study by Timmis et al. (2019) mentioned above, and ‘Scientists’ warning to humanity: microorganisms and climate change’ (Cavicchioli et al., 2019). These articles detailed current crises that humankind is facing and the frequent inability of decision‐makers to confront them and make sensible, evidence‐based policies/decisions. In many of these crises, microbes play a key role, while the lack of microbiology awareness or knowledge amongst decision‐makers hinders our ability to address them. Other timely articles by Timmis and colleagues relate to climate change. These relate to soil health—‘The soil crisis: the need to treat as a global health problem and the pivotal role of microbes in prophylaxis and therapy’ (Timmis & Ramos, 2021)—or forewarn against, potential outcomes of unmitigated climate change: ‘The darkest microbiome—a post‐human biosphere’ (Timmis & Hallsworth, 2022; see Supplementary Text: ‘Global soil health, and a post‐human biosphere’). The public needs to be aware of how microbes affect climate change and how microbes are impacted by climate change so that people realise how important is to protect and manage the Earth's microbiome to achieve a sustainable future (Cavicchioli et al., 2019).

Without exception, the urgent challenges listed above are complex and require analyses over time, not least for purposes of modelling and prediction. Therefore, theory‐based studies are required to elucidate these issues, and elaborate potential solutions including articles discussed here in relation to climate change (Cavicchioli et al., 2019; Lovelock, 1972; Lovelock & Margulis, 1974; Partida‐Martínez & Heil, 2011; Rockström et al., 2009; Timmis & Hallsworth, 2022; Timmis & Ramos, 2021) and those by others including Schuur et al. (2015), Ernakovich et al. (2022), Kemp et al. (2022), and McKay et al. (2022). Collectively, such works demonstrate that quantum changes in progress and understanding come from both technical and theoretical advances, which essentially are two of the three drivers of progress (need, being the other).

## CONCLUDING REMARKS

It is perhaps serendipitous that the recent pandemic made us think about the role of scientific thinking during research studies. In September 2020 (during the COVID‐19 lockdown), evolutionary biologist Richard Dawkins (University of Oxford, UK) published a tweet about some of the important theory‐based science that took place during a lockdown related to the Black Death (the bubonic plague) of the 1660 s: ‘In 1665 Cambridge University closed because of plague. Isaac Newton retreated to rural Lincolnshire. During his 2 years in lockdown he worked out calculus, the true meaning of colour, gravitation, planetary orbits & the three Laws of Motion. Will 2020 be someone's Annus Mirabilis?’. The notion that downtime can promote scientific productivity and novelty is therefore not specific only to the COVID‐19 pandemic.

To some extent, the papers highlighted in the current study reflect the authors' research interests (see Supplementary Text ‘Research foci of the authors’) but thought experiments and other theory‐based papers can make important contributions throughout the life sciences. This is demonstrated in the current article that highlights papers representing diverse facets of biology: origins of life, the cell, evolution, biochemistry, genomics, thermodynamics in biology, biophysics of water, ecology, Earth's biosphere, climate change, space–time, methodologies and techniques, astrobiology, etc. When constructing Figures 1 and 2 of the current article, we had not anticipated that scientific thought (indicated in green) would dominate these displays—that show the scientific process and the primary types of scientific novelty—and practical experimentation (indicated in red) featured less than we had anticipated. Furthermore, we were struck that the scientific process can occur without practical experiments but not without theory‐based and thought‐based elements (Figure 1).

Although not every theory‐based study is fruitful, one could argue that thought experiments and other theory‐based studies provide additional degrees of freedom in scientific research. Of course, practical experiments involve the use of techniques and methodological approaches that can generate types of novel data not available by other means. Otherwise, there are few if any qualitative differences between comparable studies that are based on practical experiments and those based on theory. In both cases, the scientific process (Figure 1) is essentially the same and both approaches might use controls and both may require statistical analyses. Furthermore, the concepts of theory‐based versus practical experimental microbiology are in reality part of a continuous and seamless spectrum of routes that can be taken to obtain novel scientific findings, and both practical experiments and theory‐based research act to balance, shape, and drive the scientific body of knowledge that we have. We believe that all the different types of scientific approaches are essential to microbiology. In relation to scientific novelty beyond the experiment, we believe that modern science—for all its achievements—can learn from the natural philosophy of the past.

## AUTHOR CONTRIBUTIONS

**John E. Hallsworth:** Conceptualisation (lead); investigation (lead); project administration (lead); supervision (lead); validation (lead); visualisation (lead); writing – original draft (lead); writing – review and editing (lead). **Zulema Udaondo:** Conceptualisation (supporting); investigation (supporting); visualisation (supporting); writing – original draft (supporting); writing – review and editing (supporting). **Carlos Pedrόs‐Aliό:** Conceptualisation (supporting); visualisation (supporting); writing – original draft (supporting); writing – review and editing (supporting). **Juan Höfer:** Investigation (supporting); validation (supporting); writing – original draft (supporting); writing – review and editing (supporting). **Kathleen C. Benison:** Investigation (supporting); validation (supporting); writing – original draft (supporting); writing – review and editing (supporting). **Karen G. Lloyd:** Investigation (supporting); validation (supporting); writing – original draft (supporting); writing – review and editing (supporting). **Radamés J. B. Cordero:** Investigation (supporting); writing – original draft (supporting); writing – review and editing (supporting). **Claudia B. L. de Campos:** Conceptualisation (supporting); investigation (supporting); validation (supporting); writing – review and editing (supporting). **Michail M. Yakimov:** Investigation (supporting); validation (supporting); writing – original draft (supporting). **Ricardo Amils:** Conceptualisation (supporting); investigation (supporting); validation (supporting); writing – original draft (supporting); writing – review and editing (supporting).

## FUNDING INFORMATION

No funding information provided.

## CONFLICT OF INTEREST Statement

The authors declare that they have no conflict of interest.

## Supporting information


Appendix S1

## Endnotes

^1^
In some cases, undergraduate dry projects can even yield publications. Examples from the research groups of the authors include the projects of Deidre A. LaClair (Benison and LaClair, 2003), Guillermo Mateos (Mateos et al., 2022), Megan K. May (Lloyd et al., 2013), Erin M. McCammick (McCammick et al., 2010), Colin D. McClure (Stevenson et al., 2015), Callum J. O'Kane (Stevenson et al., 2017), Callum C.J.D. Lee (Lee et al., 2018), Abiann D.R. Lewis (Hamill et al., 2020), and Jonathan A. Cray (Cray et al., 2013a that is discussed in *Natural philosophy can drive scientific progress* below, and Cray et al., 2013b; one of these from the final‐year research project and one from a summer‐vacation project). The study by Mateos et al. (2022) was done during the COVID‐19 pandemic lockdown.

^2^
This term comes from the Latin *experiri* meaning, quite simply, ‘try’

^3^
In the current article, these famous and classic studies share some qualitative similarities in their theoretical approaches with those used in the contemporary studies that are also highlighted. Beyond this similarity, we do not suggest that these contemporary studies will necessarily have impacts comparable to those of the classic works cited.

^4^
There were relatively few research groups working on the (in‐vivo) biology of chaotropicity during the 20‐year period following the initial (1995) submission of Hallsworth (1998). However, research activity in this field is now rapidly increasing, and the works based on that study (Supplementary Text ‘Work that followed from a theory‐based study of ethanol stress’) have now been cited by ~3000 other papers (Google Scholar; August, 2022), used as the basis of further experimental studies (e.g., Belilla et al., 2019; Cubillos et al., 2019; Heinz et al., 2022; Martínez et al., 2021; Rubin et al., 2017; Zajc et al., 2014), and feature in more than 50 textbooks. A more‐recent explanation of ethanol‐induced stress by the J.E.H. group was published by Cray et al. (2015b) and the distinctions between toxicity and stress are examined in more detail in Noel et al. (2023).

^5^
The term *situational–functional* describes functionality within any prevailing or given context, albeit this term is rarely used (McCammick et al., 2010; Tsukuba Language Group, 1995).

^6^
Artificial intelligence methodologies have also been successfully employed to predict the physical properties of proteins from their gene sequences. This has culminated in the development of the AlphaFold pipeline (Jumper et al., 2021) which is capable of predicting, with atomic accuracy, the three‐dimensional structure of a protein using its amino‐acid sequence as the input.

^7^
This work is itself a theory‐based and seminal study of plant evolution and ecology that put forward the theory that crop plants evolved from plant weeds, amongst other findings, and led to the late development of fields such as palaeobotany.
