# Supplementary material for: Scientific novelty beyond the experiment
Source: Microb Biotechnol. 2023 Feb 14;16(6):1131–73. doi: 10.1111/1751-7915.14222 (PMC10221578; doi:10.1111/1751-7915.14222)
Supplement: Supplementary file 1 — Appendix S1 [file MBT2-16-1131-s001.docx]

**Supplementary Information for:**

**Scientific novelty beyond the experiment**

John E. Hallsworth^1,^*, Zulema Udaondo^2^, Carlos Pedrós-Alió^3^, Juan Höfer^4^, Kathleen C. Benison^5^, Karen G. Lloyd^6^, Radamés J. B. Cordero^7^, Claudia B. L. de Campos^8^, Michail M. Yakimov^9^, and Ricardo Amils^10,11^

*^1^Institute for Global Food Security, School of Biological Sciences, Queen’s University Belfast, 19 Chlorine Gardens, Belfast, BT9 5DL, UK.*

*^2^Department of Biomedical Informatics, University of Arkansas for Medical Sciences, 72205 Little Rock, AR, USA.*

*^3^Department of Systems Biology, Centro Nacional de Biotecnología (CSIC), c/ Darwin 3, 28049 Madrid, Spain.*

*^4^Escuela de Ciencias del Mar, Pontificia Universidad Católica de Valparaíso, 2374631, Valparaíso, Chile.*

*^5^Department of Geology and Geography, West Virginia University, Morgantown, West Virginia, 26506, USA.*

*^6^Microbiology Department, University of Tennessee, Mossman Building Rm. 307, Knoxville, TN, 37996, USA.*

*^7^Department of Molecular Microbiology and Immunology, Johns Hopkins Bloomberg School of Public Health, 615 North Wolfe Street, Baltimore, Maryland, 21205, USA.*

*^8^Institute of Science and Technology, Universidade Federal de Sao Paulo (UNIFESP), 330 Talim St., São José dos Campos 12231-280, SP, Brazil.*

*^9^Institute of Polar Sciences, ISP-CNR, 98122, Messina, Italy.*

*^10^Department of Molecular Biology, Centro de Biología Molecular Severo Ochoa (CSIC-UAM), Nicolás Cabrera nº 1, Universidad Autónoma de Madrid, Cantoblanco, 28049 Madrid, Spain.*

*^11^Department of Planetology and Habitability, Centro de Astrobiología (INTA-CSIC), 28850 Torrejón de Ardoz, Spain.*

**Contents:**

Supplementary Text: Institutional views of research articles versus review articles

Supplementary Text: Research foci of the authors

Supplementary Text: Diversity and ecology of marine microbes

Supplementary Text: Acid brines and habitability of Mars

Supplementary Text: Oakes Ames (1874-1950)

Supplementary Text: Inter- and trans-disciplinary scientists

Supplementary Text: “The Concept of Mind”

Supplementary Text: Microbial weeds versus plant- and animal weeds

Supplementary Text: Towards an understanding of keystone microbes

Supplementary Text: Cellular stress and toxicity are conceptually and mechanistically distinct

Supplementary Text: Work that followed from a theory-based study of ethanol stress

Supplementary Text: “Can machines think?”

Supplementary Text: The surface of Mars

Supplementary Text: A note on planetary protection

Supplementary Text: Candidate Phyla Radiation

Supplementary Text: Quantification of competitive interactions

Supplementary Text: “The Child’s Vision of the World”

Supplementary Text: “Training Spontaneity Through the Intellect”

Supplementary Text: Early developments in artificial intelligence

Supplementary Text: Attitudes of journals and funding bodies

Supplementary Text: Daniel C. Dennett III

Supplementary Text: Global soil health, and a post-human biosphere

Supplementary References

***For correspondence.** E-mail: johnhallsworth@yahoo.com

**Supplementary Text****: Institutional views of research articles versus review articles**

A Google search for ‘research article vs. review article’ (18 August, 2022) produced many hits including those mentioned below from journals, publishers, and universities. Some journals and funding bodies have liberal policies favouring innovative science (regardless of paper format) or promoting inter- and transdisciplinarity (for examples, see the *Implications and perspectives* section of the main text). In relation to this Google search, however, each institution below states a view that research articles (termed “primary literature”) report original research whereas review articles (termed “secondary literature”) are descriptive and/or unoriginal. However, many published research articles—most of which report practical experiments—are in reality not scientifically novel (as discussed by Chu and Evans, 2021); this may be equally true of most theory-based studies. It is the view of the authors that review articles such as those highlighted in the current article are original (see Figure 2).

From *Frontiers in Microbiology* ‘For Authors, Article Types’ webpage:

“**Original Research**

Original Research articles report on primary and unpublished studies. Original Research may also encompass confirming studies and disconfirming results which allow hypothesis elimination, reformulation and/or report on the non-reproducibility of previously published results…

**Systematic Review**

Systematic Review articles present a synthesis of previous research, and use clearly defined methods to identify, categorize, analyze and report aggregated evidence on a specific topic. Included in this article type are meta-syntheses, meta-analyses, mapping reviews, scoping reviews, systematic reviews, and systematic reviews with a meta-analysis…Systematic Reviews must not include unpublished material (unpublished/original data, submitted manuscripts, or personal communications) and may be rejected in review or reclassified, at a significant delay, if found to include such content.”

(*Frontiers in Microbiology* ‘For Authors, Article Types’ webpage, accessed 18 August 2022: https://www.frontiersin.org/journals/microbiology/for-authors/article-types).

From *Journal of Applied Microbiology* ‘Author Guidelines’ webpage:

**“ARTICLE TYPES**

**Original Articles**

Original Articles comprise most of the Journal and should have as their aim the development of concepts as well as the recording of facts. The manuscript should be prepared for a wide readership and as far as possible should present novel results of a substantial programme of research.

**Review Articles**

Review Articles will present a substantial survey with an adequate historical perspective of the literature on some facet of applied microbiology. We would prefer to see a distillation of early and present work within the field to show progress and explain the present interest and relevance. The manuscript should not be simply a review of past work or be concentrated largely on unpublished results.”

(*Journal of Applied Microbiology* ‘Author Guidelines’ webpage, accessed 18 August 2022:

https://sfamjournals.onlinelibrary.wiley.com/hub/journal/13652672/homepage/forauthors.htm).

From publisher Taylor & Francis ‘Author Services’ webpage:

# “What are the different types of research article?

## A guide for early career researchers

### **Research article**

Original research articles are the most common type of journal article. They’re detailed studies reporting new work and are classified as primary literature. You may find them referred to as original articles, research articles, research, or even just articles, depending on the journal.

Typically, especially in STEM subjects, these articles will include Abstract, Introduction, Methods, Results, Discussion, and Conclusion sections. However, you should always check the instructions for authors of your chosen journal to see whether it specifies how your article should be structured. If you’re planning to write an original research article, take a look at our guidance on writing a journal article.

### **Review article**

Review articles provide critical and constructive analysis of existing published literature in a field. They’re usually structured to provide a summary of existing literature, analysis, and comparison. Often, they identify specific gaps or problems and provide recommendations for future research.

Unlike original research articles, review articles are considered as secondary literature. This means that they generally don’t present new data from the author’s experimental work, but instead provide analysis or interpretation of a body of primary research on a specific topic. Secondary literature is an important part of the academic ecosystem because it can help explain new or different positions and ideas about primary research, identify gaps in research around a topic, or spot important trends that one individual research article may not.

There are three main types of review article:

1. **Literature review** – presents the current knowledge including substantive findings as well as theoretical and methodological contributions to a particular topic.
2. **Systematic review** – identifies, appraises and synthesizes all the empirical evidence that meets pre-specified eligibility criteria to answer a specific research question. Researchers conducting systematic reviews use explicit, systematic methods that are selected with a view aimed at minimizing bias, to produce more reliable findings to inform decision making.
3. **Meta-analysis** – a quantitative, formal, epidemiological study design used to systematically assess the results of previous research to derive conclusions about that body of research. Typically, but not necessarily, a meta-analysis study is based on randomized, controlled clinical trials.”

(Taylor & Francis ‘Author Services’ webpage, accessed 18 August 2022:

https://authorservices.taylorandfrancis.com/publishing-your-research/writing-your-paper/different-types-of-research-articles/#:~:text=Review%20article,-Review%20articles%20provide&text=Unlike%20original%20research%20articles%2C%20review,research%20on%20a%20specific%20topic.)

From publisher Emerald Publishing (Bingley, England, UK) ‘Authoring, Editing & Reviewing’ webpage:

## “**What is a literature review?**

Essentially, it is a description of work that has already been published in a particular field or on a specific topic. There are two main types of literature review:

**Research literature review** – This doesn’t contain new research but looks at experiments already published and reports on their findings. It gives an overview of what has been said, who the key writers are, the prevailing theories and hypotheses, the questions being asked, and the methods and methodologies that have proved useful.

**Systematic or evidence-based literature review** – Especially popular in medicine, these reviews are designed to find the best form of intervention, or explore summaries and critiques leading to better future practice.”

(Emerald Publishing ‘Authoring, Editing & Reviewing’ webpage, accessed 18 August 2022: https://www.emeraldgrouppublishing.com/how-to/authoring-editing-reviewing/write-a-literature-review).

From Louisiana State University (LA, USA) ‘Libraries’ webpage (given on this webpage in the form of a quotation):

**“Research Articles versus Review Articles**

A **research article** is a primary source...that is, it **reports the methods and results of an original study performed by the authors**. The kind of study may vary (it could have been an experiment, survey, interview, etc.), but in all cases, raw data have been collected and analyzed by the authors, and conclusions drawn from the results of that analysis.

A **review article** is a secondary source...it is written about other articles, and does not report original research of its own. Review articles are very important, as they draw upon the articles that they review to suggest new research directions, to strengthen support for existing theories and/or identify patterns among existing research studies. **For student researchers, review articles provide a great overview of the existing literature on a topic.** If you find a literature review that fits your topic, take a look at its references/works cited list for leads on other relevant articles and books!"

From https://apus.libanswers.com/faq/2324, "What's the difference between a research and a review article?"

(Louisiana State University ‘Libraries’ webpage, accessed 18 August 2022: https://guides.lib.lsu.edu/c.php?g=376856&p=2550574).

From Concordia University (Qc, Canada) ‘Library’ webpage:

**“Review vs. Research Articles**

**Research Papers.** A research article describes a study that was performed by the article’s author(s). It explains the methodology of the study, such as how data was collected and analyzed, and clarifies what the results mean. Each step of the study is reported in detail so that other researchers can repeat the experiment…

**Review Papers.** Review articles do not describe original research conducted by the author(s). Instead, they give an overview of a specific subject by examining previously published studies on the topic. The author searches for and selects studies on the subject and then tries to make sense of their findings. In particular, review articles look at whether the outcomes of the chosen studies are similar, and if they are not, attempt to explain the conflicting results. By interpreting the findings of previous studies, review articles are able to present the current knowledge and understanding of a specific topic.

Since review articles summarize the research on a particular topic, students should read them for background information before consulting detailed, technical research articles…”

(Concordia University ‘Library’ webpage, accessed 18 August 2022: https://www.concordia.ca/library/guides/exercise-science/review-vs-research.html).

From Washington University in St. Louis (MO, USA) ‘Library Services’ webpage:

**“Literature Review: A Definition**

**What is a literature review, then?**

A literature review discusses and analyses published information in a particular subject area. Sometimes the information covers a certain time period.

A literature review is more than a summary of the sources, it has an organizational pattern that combines both summary and synthesis. A summary is a recap of the important information of the source, but a synthesis is a re-organization, or a reshuffling, of that information. It might give a new interpretation of old material or combine new with old interpretations. Or it might trace the intellectual progression of the field, including major debates. And depending on the situation, the literature review may evaluate the sources and advise the reader on the most pertinent or relevant.

### **But how is a literature review different from an academic research paper?**

While the main focus of an academic research paper is to support your own argument, the focus of a literature review is to summarize and synthesize the arguments and ideas of others. The academic research paper also covers a range of sources, but it is usually a select number of sources, because the emphasis is on the argument. Likewise, a literature review can also have an "argument," but it is not as important as covering a number of sources. In short, an academic research paper and a literature review contain some of the same elements. In fact, many academic research papers will contain a literature review section. What aspect of the study (either the argument or the sources) that is emphasized determines what type of document it is.”

(Washington University in St. Louis ‘Library Services’ webpage, accessed 18 August 2022: https://libguides.wustl.edu/c.php?g=47119&p=302677#:~:text=A%20literature%20review%20discusses%20and%20analyses%20published%20information,organizational%20pattern%20that%20combines%20both%20summary%20and%20synthesis.

##### From Welcome to the Le Moyne College (NY, USA) ‘Noreen Reale Falcone Library’ webpage:

“**Course Guides, Primary Research vs. Review**

##### **What is a Primary Research Article?**

In a primary research article, author(s) present a new set of findings from original research after conducting an original experiment…Primary research articles are also referred to as original research or research articles.

#### **What is a Review Article?**

Review articles do not report new experiments. Rather, they attempt to provide a thorough review of a specific subject by assessing either all or the best available scholarly literature on that topic. By considering the findings of many primary research articles, review articles can provide a comprehensive background and analysis of all the available evidence on a subject. A single review article will summarize, analyze, and discuss the results of numerous primary research articles all at once, and often will provide comparisons with respect to each other.

For a review article, the authors do not design an experiment and carry it out in a lab. Rather, they search for, find, and read numerous primary research articles on a particular topic. Then, they organize them into a cohesive narrative that provides an overall summary and analysis of a topic.

Review articles can help explain the basics of a particular area of science, provide an overview of all of the research that has been conducted on a particular topic, and/or provide insight into current topics of scholarly disagreement. They can also help identify where there are gaps in research and scientific knowledge.”

(Le Moyne College ‘Noreen Reale Falcone Library’ webpage, accessed 18 August 2022: https://resources.library.lemoyne.edu/courses/bio218/PrimaryResearchVsReview).

**Supplementary Text: Research foci of the authors**

The authors’ research interests/expertise, which likely influenced the choice of the studies highlighted in the current article, are summarised below.

Ricardo Amils focuses on acidophiles, geomicrobiology of the deep continental subsurface and other extreme environments, and astrobiology. He also has interests in evolutionary theory and biosphere function.

Kathleen C. Benison works on the geology, geochemistry, and geobiology of extreme acid saline lake systems on Earth and Mars. Her fieldwork and laboratory-based experiments are augmented by theory-based studies.

Claudia B. L. de Campos works on the controls for fungal growth rate, differentiation and metabolism under stress. Her bioinformatic works and laboratory-based experiments are augmented by theory-based studies.

Radamés J. B. Cordero studies the role of fungal melanin in radiation energy absorption and dissipation to help predict changes in fungal ecology and develop melanin-based biotechnologies in Earth and space applications. His fieldwork and laboratory-based experiments are augmented by theory-based studies.

John E. Hallsworth focuses on the ways in which biology responds to biophysical and other constraints, including studies pertaining to the biophysical limits of microbial life on Earth and aspects of astrobiology. His fieldwork and laboratory-based experiments are augmented by theory-based studies.

Juan Höfer works on the interaction between marine microorganisms and physico-chemical conditions, and how variations in the latter (including those caused by global climate change) will impact ecosystem functioning through changes in the microbial community. His fieldwork and laboratory-based experiments are augmented by theory-based studies.

Karen G. Lloyd studies the physiology and compositions of subsurface microbial communities in deep marine sediments, natural seeps, and permafrost using geochemistry, biological activity measurements, and multi-omics tools. Her fieldwork and laboratory-based experiments are augmented by theory-based studies.

Carlos Pedrós-Alió is a microbial ecologist using ‘omics to reveal strategies of microorganisms that inhabit aquatic environments including thermal springs, solar salterns, and the oceans. His fieldwork and laboratory-based experiments are augmented by theory-based studies.

Zulema Udaondo is a computational biologist focused on bacterial genomics and pan-genomics of environmental and nosocomial microorganisms. She uses laboratory-based experiments and computational technologies and other theory-based approaches.

Michail M. Yakimov works on genomics, physiology, and ecology of halophiles and other extremophilic microbes. His fieldwork and laboratory-based experiments are augmented by hybrid (theory-based + practical) studies.

**Supplementary Text: Diversity and ecology of marine microbes**

At the 1992 International Society for Microbial Ecology Symposium (Barcelona, Spain), theoretical ecologist Robert M. May (then at the University of Oxford, UK) made a simple statement about the Earth’s biodiversity. He declared that: “Whereas we have a catalogue of all the bodies our instruments can detect in space, we ignore with how many living beings we share the Earth”. C.P.-A., present at the meeting, was both impressed and shocked by this statement. He realised that our collective ignorance about how many microbial species there are was considerable. Only a few thousand species had been described after painstakingly isolating and characterising them in pure culture. At about the same, time molecular methods were only just being applied to the environment for the first time, and it turned out that the most-abundant bacteria in nature were not those available in pure culture collections (Giovannoni *et al*., 1990). During the ensuing decade, C.P.-A. realised that studies of microbial diversity were a miscellaneous collection of different approaches, techniques, and ideas that had little in common with each other. For example, estimates of bacterial diversity in the oceans ranged from just a few thousand (Hagström *et al*., 2002) to a million (Curtis *et al*., 2002), and up to one 1000 million for the whole planet (Dykhuizen, 1998). Some studies described the bacteria appearing in culture media whereas other studies described the clones in a DNA-clone library, so it seemed impossible to compare different studies in a meaningful way. Furthermore, additional practical experimental studies would not solve this issue. Instead, a common theoretical frame was needed.

C.P.-A. realised that the well-known tool that is called the ‘rank-abundance curve’, and is used in general ecology, provided such a framework. He identified this tool as the most-direct and effective way to place within a common framework molecular and pure culture studies as well as revealing the properties that being abundant or rare confer on prokaryotes. Based on this approach, he published the following study: ‘Marine microbial diversity: can it be determined?’ (Pedrós-Alió, 2006). In these rank-abundance curves, species taxa (whether identified from cultures or from molecular methods) are arranged along the x-axis ranked in order of their abundance, and the numbers of individuals of each species are placed along the y-axis. The curves usual decrease exponentially in all ecosystems indicating that whereas only a few species dominate the community, there is a large number of very rare species.

Pedrós-Alió (2006) proposed that the small number of abundant species had to be functionally significant in the carbon- and energy flow of ecosystems, while the very large number of rare taxa provided a reservoir of biodiversity. There was a three-to-four orders-of-magnitude difference in the abundances of these two groups, and it seems implausible that a taxon making up less than 0.01% of the community might play a key role in the functioning of the ecosystem. In fact, a *Leeuwenhoeckiella blandensis* pure culture isolated from the Northwestern Mediterranean was found by amplicon sequencing to make only 0.003% of all the amplicons (Pedrós-Alió, 2013). This framework also provided an understanding of the ecological strategies of rare taxa; by being rare they were relatively at low risk of large-scale losses due to factors such as viral attack and predation. Therefore, they could remain for extensive time periods, and ultimately proliferate sufficiently to become abundant if conditions became favourable. This explained some of the extraordinary dynamics and flexibility of microbial communities (e.g., Cray *et al*., 2013a; Mestre and Höfer, 2021). The methodological result was that people had a map of where their studies of diversity fit. That the framework provided by Pedrós-Alió (2006) has been utilised widely by scientists, as reflected in the over ~800 citations that it received to date (Google Scholar; August, 2022).

**Supplementary Text: Acid brines and habitability of Mars**

One important thought experiment was done by planetary geochemist Benton C. Clark (The Space Science Institute, CO, USA): ‘Acid waters as agents of change on a cold early Mars’ (Clark, 1994). He has worked on each of the NASA Mars rover missions, and on several of the NASA Mars lander missions. In the 1970s, the NASA Mars Viking I and II landers provided data about the Martian atmosphere and regolith, providing fundamental physical and chemical information about the planet’s surface (Clark, 1979). Some years later, Clark (1994) utilised these data to carry out his thought experiment. He asked what compositions, consistent with those measurements, would exist as (a) stable liquid(s) on Mars. He calculated that 39% v/v H_2_SO_4_ would be the most-likely stable liquid (Clark, 1994). Based on this, Clark’s hypothesis for acid waters on Mars contrasted with the decades-old assumption that river channels, or ‘canals’ on Mars, were formed by the flow of pure liquid water (virtually 100% H_2_O).

This widely-held view about dilute-, close-to-100%, water on Mars, combined with Clark’s 39% v/v H_2_SO_4_ thought-experiment result, inspired a set of laboratory experiments to test how 39% v/v H_2_SO_4_ would act as a physical sedimentology agent via the entrainment, transport, and deposition of sand grains by one of us (K.C.B.) (Benison *et al*., 2008). Different liquids, including 39% v/v H_2_SO_4_, 100% H_2_SO_4_, and 100% H_2_O, as well as saline solutions of each of these liquids, were allowed to flow over a slope of loose sand grains. This revealed that H_2_SO_4_, with its higher density and viscosity, behaves differently from H_2_O. The morphology of channels and fans made in the laboratory with 39% v/v H_2_SO_4_ were most similar to those observed on Mars (Benison *et al*., 2008). Clark’s thought experiment helped to lead the way for today’s acceptance that acid brines once existed on Mars’s surface (i.e., Burns, 1987, 1994; Benison and LaClair, 2003; Squyres *et al*., 2004; Benison, 2006; Benison and Bowen, 2006; Ehlmann *et al*., 2016). Geological, geochemical, and microbiological analyses of terrestrial acid saline lakes as Mars-analogues were also based on the Clark (1994) thought experiment (i.e., Benison *et al*., 2007; Mormile *et al*., 2009; Zaikova *et al*., 2018). These studies, in turn, provide an understanding of potential habitability on Mars and give a direction to the most-likely mineral and rock samples that might have preserved putative Martian lifeforms (Figure 2; Benison, 2019).

**Supplementary Text: Oakes Ames (1874-1950)**

From the chapter ‘Uneconomic Botany’ of *Plants, Man and Life* (Anderson, 1952):

“The germ of this book…was a course in Economic Botany…by Professor Oakes Ames [Harvard University, USA]. Since he scarcely mentioned wheat, said nothing whatever about lumber, and gave only a passing nod to such staples as rice and maize, I, in those days, joined the other unappreciative recipients of these pearls in referring to the course derisively as Uneconomic Botany. I have since learned better...

[Professor Ames] was one of the few people in this country to take a really intelligent interest in cultivated plants and in his later years published a small book *Economic Annuals and Human Cultures* [Ames, 1939], which is just now beginning to find an appreciative audience. If a scientist is one jump ahead of his fellows in his thinking he is usually their acknowledged leader; if he is two jumps ahead he is thought to be eccentric and rather screwball but sometimes receives belated recognition in his old age. If he is three jumps ahead he is ignored, though posterity may eventually get around to appreciating his evidence as it did with Gregor Mendel…

Ames had the mind of a scholar and the soul of an artist. Anything he ever did was done to perfection by his own aristocratic standards and not according to the conventions of his colleagues or his students...

Though [he] scrupulously avoided even hinting at the fact, he was technically much better equipped than his self-sufficient graduate students to judge how far his course in Economic Botany dealt with matters which were of truly economic importance…He realized…that modern technology finds new uses for ancient plant products as frequently as it replaces old ones. He told us about balsa wood, the first time most of us had ever heard the name. Five years later it was important in world trade. He went into arrow poisons in great detail; since then a great commercial drug concern has invested a small fortune in bringing an arrow poison, curare, to the practical service of modern medicine, and has done it as a hardheaded practical business venture. Ames told us about fish poisons. We thought it amusing but of no practical significance for us; within a decade such fish poisons as derris root were in mass production in various parts of the tropics to provide one of the most important ingredients in modern insecticides…“

*Plants, Man and Life* (Anderson, 1952) contents:

Man and His Transported Landscapes

The History of Weeds — A Detective Story

The Greater Paradox

The Clue from the Root Tips

The Clue from Diversity, or Science and the Bureaucrats

How to Measure an Avocado

Budget vs. Scholarship

Uneconomic Botany

Dump Heaps and the Origin of Agriculture

A Roster of our Most Important Crop Plants and Their Probable Origins

Sunflowers — The One Native American Crop

Adventures in Chaos

*Suggested Reading*

*Index*

*Economic Annuals and Human Cultures* (Ames, 1939) contents:

Preface

The Significance of the Angiosperm Seed

Antiquity of Economic Annuals

Economic Plants Through the Pleistocene

The more important Economic Annuals

Plants as Measures of cultural Time

Index

**Supplementary Text: Inter- and trans-disciplinary scientists**

Examples of sciences who are inter- or trans-disciplinary—and can be viewed as polymaths or Renaissance intellectuals—include (from previous centuries) Hildegard von Bingden*,* George Washington Carver*,* Charles R. Darwin, Galileo di V.B. de' Galilei, James Africanus Beale Horton, F.W.H. Alexander von Humboldt, Plato (son of Ariston), and Leonardo P. da Vinci; (from the recent past) James E. Lovelock, Lynn Margulis, P. Buford Price, and Humberto Maturana Romesín; and (from the present day), in the authors’ judgement, Asmeret A. Berhe (University of California, CA, USA), Daniel C. Dennett III (Tufts University, MA, USA), Mae C. Jemison (NASA astronaut, USA), Sang Y. Lee (Korea Advanced Institute of Science and Technology, KAIST, South Korea), Christopher P. McKay, John A. Raven (University of Dundee, Scotland, UK), and Rekha S. Singhal (Institute of Chemical Technology–Mumbai, ICT–Mumbai, India).

**Supplementary Text: “The Concept of Mind”**

From the Introduction of *The Concept of Mind* (Ryle, 1949):

**“**This book offers what may with reservations be described as a theory of the mind. But it does not give new information about minds. We possess already a wealth of information about minds, information which is neither derived from, nor upset by, the arguments of philosophers. The philosophical arguments which constitute this book are intended not to increase what we know about minds, but to rectify the logical geography of the knowledge which we already possess.”

*The Concept of Mind* (Ryle, 1949) contents:

Introduction
I. DESCARTES’ MYTH

The Official Doctrine

The Absurdity of the Official Doctrine

The Origin of the Category Mistake

Historical Note

II. KNOWING HOW AND KNOWING THAT

Foreword
 Intelligence and Intellect

Knowing How and Knowing That

The Motives of the Intellectualist Legend

’In My Head’

The Positive Account of Knowing How

Intelligent Capacities versus Habits

The Exercise of Intelligence

Understanding and Misunderstanding

Solipsism
III. THE WILL

Foreword
 The Myth of Volitions

The Distinction Between Voluntary and Involuntary

Freedom of the Will

The Bogy of Mechanism

IV. EMOTION

Foreword
 Feelings versus Inclinations

Inclinations versus Agitations

Moods
 Agitations and Feelings

Enjoying and Wanting

The Criteria of Motives

The Reasons and Causes of Actions

Conclusion
V. DISPOSITIONS AND OCCURENCES

Foreword
 The Logic of Dispositional Statements

Mental Capacities and Tendencies

Mental Occurrences

Achievements
VI. SELF-KNOWLEDGE

Foreword
 Consciousness
 Introspection
 Self-Knowledge Without Privileged Access

Disclosure by Unstudied Talk

The Self

The Systematic Elusiveness of ’I’

VII. SENSATION AND OBSERVATION

Foreword
 Sensations
 The Sense Datum Theory

Sensation and Observation

Phenomenalism
 Afterthoughts
VIII. IMAGINATION

Foreword
 Picturing and Seeing

The Theory of Special Status Pictures

Imagining
 Pretending
 Pretending, Fancying, and Imagining

Memory
IX. THE INTELLECT

Foreword
 The Demarcation of the Intellect

The Construction, Possession, and Utilisation of Theories

The Application and Misapplication of Epistemological Terms

Saying and Teaching

The Primacy of the Intellect

Epistemology
X. PSYCHOLOGY

The Programme of Psychology

Behaviourism
INDEX

**Supplementary Text: Microbial weeds versus plant- and animal weeds**

To the botanist, plant weeds have a precise definition, a common evolutionary trajectory, and many shared traits (Long, 1932; Anderson, 1952; Baker, 1965; Hill, 1977; Table 1 of Cray *et al*., 2013a). Similarly, microbial weeds have a precise scientific/biological definition, a particular ecology and evolutionary biology, and many shared traits (Hallsworth, 1998; Cray *et al*., 2013a; 2015a; Oren and Hallsworth, 2014). In animal science weed species have also been well-characterised, including humans (Meindl *et al*., 2018).

For microorganisms, weeds have been defined as: “microbial species able to dominate communities that develop in open (microbial) habitats” (Cray *et al*., 2013a) whereas plant weeds are those that primarily occur in open habitats (Baker, 1965). J.E.H. was aware that plant weeds are found primarily in open habitats or disturbed ground—usually bare fertile soil—and that they typically have particular phenotypic and genetic traits. Several years later, J.E.H. worked on the yeast *S. cerevisiae* and noticed remarkably similar behaviour to that of plant weeds. He briefly mentioned this in a discussion ‘Ethanol and microbial ecology’ of his review paper Hallsworth (1998) that is discussed in the main text, and revisited the topic some years later whilst working with Jonathan A. Cray (an undergraduate student who carried out his final-year research project by working on the microbial weeds topic), this time in the context of phylogenetically diverse microbes and various environmental conditions/ habitat types (Cray *et al*., 2013a). Open habitats, understood for about 50 to 100 years in the animal and plant sciences, had never been identified in the context of microbes yet, arguably, they are more important in microbiology than botany or zoology. For a full definition of open habitats of microbes, see the section ‘Open habitats’ in Cray *et al*. (2013a).

**Supplementary Text: Towards an understanding of keystone microbes**

Keystone species are those which have a disproportionately large impact on ecosystem functioning despite their relatively low abundance, and were first described for an animal species (Paine, 1969). The innovative study by Paine revealed that the biodiversity of shoreline ecosystems can decline more than 50% when a single species (seastar, *Pisaster ochraceus*) was removed, and so first described the keystone concept. Keystone ecology has also been characterised in plants (e.g., Koski *et al*., 2015), but only relatively recently for microbial species (Hajishengallis *et al*., 2011; Ze *et al*., 2012; Vick-Majors *et al*., 2014; Banerjee *et al*., 2016). However, there was no clear definition of a keystone species in relation to microbial ecology, and no standard methodology to demonstrate whether a microbe would fall into this category.

Therefore, Banerjee and colleagues proposed a definition to avoid such uncertainty and recommended a system of standardised network scores (Berry and Widder, 2014) that can be used to statistically identify keystone taxa. Banerjee *et al*. (2018) proposed a range of experimental approaches that can be employed to identify and validate keystone taxa. They discovered evidence of keystone microbial taxa across different types of microbial habitats (an observation also made of microbial weeds in the study of Cray *et al*.). For example, several members of the bacterial orders Rhizobiales and Burkholderiales orders were consistently found as keystone taxa in many soil-microbiology studies. Therefore, whereas soils and soil microbiomes are diverse and highly complex, there are specific microbial taxa that commonly play a disproportionately important ecological role.

We asked Samiran Banerjee (North Dakota State University, ND, USA) what inspired the 2018 study. He had begun doing network analysis of soil microbiomes in 2013 and noticed that in each network there are certain groups of operational taxonomic units (OTUs) that are much more connected than others, with many of these groups consistent across networks (each network equivalent to a subset of a microbial community). Banerjee then came across and read the original 1969 article on keystone animal species which helped to clarify his thinking in relation to keystone microbes (Paine, 1969). In relation to microbiology, metagenomic datasets can inadvertently give a flat structural view of microbial ecosystems, where vast numbers of taxa appear to have the same level of importance within the system. However, with the findings of Paine (1969) in mind, Banerjee’s network analyses of soil microbiomes indicated that a small number of taxa exert stronger influence within the microbial ecosystem than others. This mirrored findings of Hajishengallis and colleagues in relation to the human microbiome (Hajishengallis *et al*., 2011). As Banerjee explained to us, however, statistical networks are based on correlations but a correlation does not necessarily imply causation. Statistically-identified keystone taxa must be confirmed/validated using wet-biology experimental approaches.

Scientists from different fields are now using the Berry and Widder (2014) method recommended by Banerjee *et al*. (2018) to identify keystone taxa for anthropogenic manipulation of a given microbiome to modify its functional characteristics. There are several studies that subsequently found keystone microbial taxa consistent with those identified by Banerjee *et al*. (2018). For example, a recent article in *Nature Genetics* reported 39 identifiable keystone taxa that exert key modulatory roles on microbial composition of the human gut microbiota even if they are not the most abundant (Qin *et al*., 2022). This is a major development because it can accelerate the development of medical interventions via manipulations of the microbiomes of human patients (Kuntz and Gilbert, 2017). It may be just matter of time before industry invests in the development of techniques that can identify and manipulate keystone taxa within various microbiomes (soil, plant, animal, human, etc.). With the latest developments in synthetic biology, for example, researchers should ideally have a set of experimental methods soon that would work for identifying keystone taxa in different microbiomes. Similarly, as the body of data of consistently identified and empirically validated keystone taxa grows, there might in future be policies to use such taxa as indicators of ecosystem health, and to protect them. Ultimately, if the scientific community can isolate microbes that can be applied to improve microbiome functioning, enhance tolerance against stressors, or cure a disease, and enhance environmental sustainability then these will be the best outcome of the study by Banerjee *et al.* (2018). The field of keystone research has found a renewed momentum and we believe that there will be an increasing number of studies identifying and validating keystone taxa in different microbiomes and ecosystems.

There is now increasing momentum in keystone research, and Banerjee *et al*. (2018) has already been cited 840 times according to Google Scholar (August, 2022). Numerous keystone bacteria have now been identified, but also some *Archaea* (Kumpitsch *et al*., 2021) and fungi including mycorrhizal fungi (Banerjee *et al*., 2019) and the ascomycete *Metarhizium*, as keystone species (Brancini *et al*., 2022). Banerjee told us that “Many people shared the article on social media after its publication, but there were also researchers that immediately criticised the keystone taxa concept. Criticism in science is, in general, a positive thing and no new concept gets established instantaneously” as for Sagan (1967) for example; see the main text.

The study by Banerjee *et al*. (2018) has reached audiences beyond those who work on keystone microbes; it is ranked by Altmetric as having the 4^th^ highest score out of 45 tracked articles of a similar age in *Nature Reviews Microbiology*. The keystone microbes and microbial weeds studies utilised both concepts and terminology that originated from zoology and botany (respectively). In some areas of research, however, terminology and other aspects of language can hinder rather than drive scientific progress. These problems can nevertheless provide the opportunity and stimulus for scientific change.

**Supplementary Text: Cellular stress and toxicity are conceptually and mechanistically distinct**

Toxins and toxicants are substances with a target-specific mode-of-action and a minimum concentration to prevent microbial cell division as low as the pM range; they include antibiotics, heavy metals, and fungicides such as ergosterol biosynthesis inhibitors (Hallsworth *et al*., 2003a; Figures 1 and 2 of Cray *et al*., 2015a). Their specificity and potency are reflected in the terminology; for example, ‘toxicity’ is etymologically derived from the Latin *toxicum* meaning poison. By contrast, cellular stressors have a biophysical mode-of-action that commonly impacts diverse structures and processes within the cell (Figure 2 of Cray *et al*., 2015a; Hallsworth, 2018), generally requiring higher concentrations to cause cellular inhibition, depending on the exact stress mechanism (Hallsworth *et al*., 2003a; Alves *et al*., 2015; Cray *et al*., 2015a). Stressors—substances that cause cellular stress—include those substances that reduce water activity, modify the cell’s osmotic balance, exhibit chaotropic and/or hydrophobic properties, cause oxidative damage, and markedly increase ionic strength (Hallsworth *et al*., 2003a; Bhaganna *et al*., 2010; Alves *et al*., 2015; Fox-Powell *et al*., 2016; Imlay, 2019). The concept of stress is etymologically derived from the Old French word *estresse* meaning narrowness/oppression (based on the Latin *strictus* meaning drawn tight); see Hallsworth (2018) for a more-detailed discussion of this concept in relation to microorganisms. This definition is in essence equally valid for stress parameters such as temperature, pH and ultra-violet radiation. Whereas the meanings of words can sometimes evolve over time, the mechanisms and responses of microbes to toxicity and stress are in general consistent with the etymological root of these terms (Cray *et al*., 2015a; Hallsworth, 2018; Noel *et al*., 2023). Furthermore, the definitions given here are consistent with the meaning and biology of toxicity (Trinci and Ryley, 1984) and stress (Brown, 1990) as recognised by leaders of these fields throughout the history of modern science.

Despite the qualitative and mechanistic differences between toxic substances and cellular stressors, some microbiology literature uses somewhat nebulous terminology such as “toxic stress” when referring to stress parameters (e.g., van Wijngaarden *et al*., 1995); other studies use the terms *toxic* and *stress* as interchangeable (e.g., Liu *et al*., 2020); and some papers designate stressors as toxins (e.g., Mota *et al*., 2021) or *vice versa* (e.g., Rath *et al*., 2016). Indeed, it is relatively few studies that make a mechanism-based distinction between toxins/toxicants and stressors (Hallsworth *et al*., 2003a; Cray *et al*., 2015a; Valette *et al*., 2021). This said, it is not implausible that a substance might inhibit growth of cells via two distinct mechanisms, one stress-mediated and one toxicity-mediated. However, this is not common because these modes-of-inhibition generally operate at different concentrations (see above). For example, the toxicity of sodium arsenate occurs at concentrations that are orders-of-magnitude below those that would cause chaotropicity-mediated cellular stress (Hallsworth *et al*., 2003a). There are, however, a few substances that have a target-specific mode-of-action (like a toxin) yet damage the cell via a stress mechanism (oxidative stress); terms toxin–stressors (Noel *et al*., 2023). One notable example is photodynamic substances that accumulate preferentially in specific microbial taxa, and do so at specific sites in the cell (for example, the cell membrane). Upon activation by light or ultra-violet radiation, they release reactive oxygen species that often results in stress-induced cell death (Braga *et al*., 2022).

**Supplementary Text: Work that followed from a theory-based study of ethanol stress**

The review article by Hallsworth (1998) led on to a series of other studies that have thus-far included a proteomic study that revealed a global chaotropic stress response in the microbial cell (Hallsworth *et al*., 2003a); the finding that chaotropicity limits life in some locations on Earth (Hallsworth *et al*., 2007; Yakimov *et al*., 2015; Hallsworth, 2019); the discovery that chaotropicity determines temperature windows for microbial function (Chin *et al*., 2010); a scale, units and method to quantify chaotropicity (Hallsworth *et al*., 2003a; Cray *et al*., 2013b); a re-assessment of the concept and chemistry of chaotropicity (Ball and Hallsworth, 2015); the habitability of Mars (Chin *et al*., 2010; Rummel *et al*., 2014; Fox-Powell *et al*., 2016; Hallsworth, 2020); the finding that compatible solutes can reduce chaotrope stress (Hallsworth *et al*., 2003b; Bhaganna *et al*., 2010; 2016; Bell *et al*., 2013; Alves *et al*., 2015; Cray *et al*., 2015a; 2016); the identification of ‘chaophiles’ as a new class of extremophilic microbes (Hallsworth *et al*., 2007; Williams and Hallsworth, 2009); impacts of chaotropes on growth kinetics (e.g., Hallsworth *et al*., 1998; 2003a; 2007; Williams and Hallsworth, 2009; Bhaganna *et al*., 2010; Chin *et al*., 2010; Alves *et al*., 2015; Santos *et al*., 2015; Stevenson *et al*., 2017a; Hamill *et al*., 2020); the identification of chaotropicity-mediated modes-of-action for many chemical classes of inhibitory substances, including hydrophobes (Bhaganna *et al*., 2010; McCammick *et al*., 2010; Cray *et al*., 2013a; Alves *et al*., 2015; Suryawanshi *et al*., 2017); a new water-activity limit for microbial biosphere (Williams and Hallsworth, 2009; Stevenson *et al*., 2015a; 2017a; 2017b); a re-examination and redefinition of the concept and mechanics of cellular stress (Cray *et al*., 2015a; Hallsworth, 2018); an evaluation of the effects of chaotropicity on competitive interactions (Cray *et al*., 2013a; 2016); the identification of microbial weeds (Cray *et al*., 2013a; Oren and Hallsworth, 2014); microbiology in relation to the efflorescence and deliquescence of brines (Stevenson *et al*., 2015b; Lee *et al*., 2018; Hallsworth, 2020); and an analysis of how to mitigate against chaotropicity to optimise production of ethanol and butanol as biofuels (Cray *et al*., 2015a).

**Supplementary Text: “Can machines think?”**

In 1950, Turing published his article in the philosophy journal *Mind*, as a thought experiment in which he proposes to consider the now-famous question "Can machines think?". He began the paper by examining the words *machine* and *think* because he realised that using these words casually (colloquially) would be potentially misleading and so compromise the ability to address this scientific question. Rather than continue with the perplexing conundrum of defining these two words/concepts, Turing proposed reframing the question by replacing it with a question that has fewer ambiguities.

In the search for a better alternative question, Turing described in his manuscript a variation of a traditional Victorian parlour game known as the imitation game, in which the participants are two players (a human and a machine) and a human who is an interrogator. The game consists of the two players (A and B), which are placed in a room different from that of the interrogator, answering questions from which the latter. The interrogator then has to guess which of the other players is a machine and which is a human being. In addition to answering the interrogator's questions, the role of one of the players (the human) is to help the interrogator make the correct identification, whereas the other player (the machine) can lie to make the interrogator come to the wrong conclusion. In his thought experiment, Turing (1950) suggested that if the machine was capable of playing the imitation game indistinguishably from that of a human, it would be showing a level of intelligence comparable to that of a human. Hereby, Turing proposed to replace the initial question "Can machines think?" by creating a scenario that subjected the computer to the imitation game (since then known as the Turing Test), and rephrasing the question as: "Let us fix our attention on a particular digital computer C. Is it true that by modifying this computer so that it has adequate storage, suitably increasing its speed of action, and providing it with an appropriate program, C can be made to successfully play the role of A in the imitation game, with the role of B being taken over by a man?".

After proposing the imitation game, Turing (1950) also addressed the state of computer technology at the time of writing the manuscript, and the possible ramifications of seriously debating the feasibility of the existence (in a near future) of machines programmed to imitate the intellectual capabilities of an adult human mind, also including in his discussion both arguments and objections derived from theological, psychological, and philosophical points of view. Turing (1950) predicted that by the year 2000 we would be able to program computers that when playing the imitation game would have a 30% chance of making an average interrogator fail after 5 minutes of conversation. In 1990, the American inventor and philanthropist, Hugh Loebner and The Cambridge Center for Behavioral Studies (MA, USA) set up an extended version of the Turing Test, in the form of a contest, to evaluate Turing’s prediction.

Despite great advances in artificial intelligence, however, no computer has been capable of passing the Turing Test by providing responses that the human judges considered indistinguishable from those of a human. Although today the Turing Test has also received multiple criticisms from philosophers (e.g., Block, 1981; Gardner, 1983) and is not the current focus of artificial-intelligence research, his inspiring thought experiment helped to kick-start research in the artificial-intelligence field that was nascent at the time.

In recent years, large technology companies, such as Google and OpenAI, have produced a powerful new class of computer programs known as ‘large language models’ with conversational capabilities that resemble those needed to pass the Turing Test. These include OpenAI’s chatbot ChatGTP (see main text). It also should not be overlooked that Turing was far ahead of his time in his early consideration of the possible existence of intelligent computers in the near future, as well as proposing a form of evaluation for them. Turing’s article ‘Computer machinery and intelligence’ now receives hundreds of citations per year (586 in 2021: https://badge.dimensions.ai/details/id/pub.1027055246), mainly from the areas of information and computing sciences, and psychology and cognitive sciences. Indeed, his study that began with analysing the use of words now (72 years after publication) now receives citations that continue to rise dramatically year on year.

**Supplementary Text: The surface of Mars**

What we mean by a planet’s ‘surface’ has implications in the context of astrobiology, especially in relation to planetary protection policy. The Committee on Space Research (COSPAR) Policy on Planetary Protection aims to prevent cross-contamination of Earth and other planets, and has in recent years focused its attention on Mars (Kminek *et al*., 2010; Rummel *et al*., 2014). It has often been assumed that the planetary surface of Mars may potentially contain viable Martian lifeforms (see Supplementary Text ‘A note on planetary protection’). However, some recent studies have assumed that the Mars surface is biocidal, implying that any type of microbial contaminant accidentally brought from Earth would die, thus preventing the inter-planetary transfer of microbial contaminants (e.g., Schuerger *et al*., 2013, 2020; Spry *et al*., 2021).

At a planetary protection meeting protection meeting at the Lunar and Planetary Institute (Houston, TX, USA) in May 2019 (3rd COSPAR Meeting on Refining Planetary Protection Requirements for Human Missions and Addressing Planetary Protection Knowledge Gaps in Microbial and Human Health Monitoring), there was an open discussion about the fate of any microbes brought as from Earth accidental contaminants. There seemed to be a consensus that all microbe would die, so author of the current article J.E.H. made a comment that the exceptionally dry conditions combined with the low subzero temperatures would help to maintain viability. One attendee at the meeting responded that the surface of Mars is biocidal, and all microbes will be killed. Given that this view expressed by the respondent is making its way into published papers that can potentially influence planetary protection policy, J.E.H. carried out an evidence-based yet thought-based study of the *in-situ* Martian conditions, to establish what would happen to a microbe from Earth if released onto the Mars surface (Hallsworth, 2021).

This study, ‘Mars’ surface is not universally biocidal’, was carried out during free time provided by the COVID-19 lockdown. It irrefutably demonstrated that this planet’s surface is not universally biocidal and did so in part by deconstructing the linguistic concept of the planetary surface that “has qualitatively distinct definitions for different scientific disciplines, and can also have different meanings from a human viewpoint versus that of a microbial cell” (Hallsworth, 2021). According to one review of the Hallsworth (2021) manuscript, the term biocidal was misunderstood and incorrectly used in previous papers that contained “slips of terminology”. However, it was clear to J.E.H. from the discussion at the Houston meeting and from those papers claiming that Mars is biocidal that there is a genuine belief that any type of microbe from Earth accidentally released onto the Mars surface will be killed (for references and further details, see Hallsworth, 2021).

This said, J.E.H.’s inspiration was the fact that Mars’ surface mimics conditions that are used by culture collections for long-term storage of microbes (desiccation and/or temperatures down to -70°C) albeit that on Mars, ultra-violet radiation may kill some cells. However, most spores or other microbial cells that land on the planet’s surface would fall within pores of the regolith or become quickly buried/shielded by dust. Essentially, therefore, they do not remain at the absolute surface so are not exposed to ultra-violet. The low temperatures (typically −40 to −70°C) and desiccating conditions will act to maintain viability for indefinite periods, and this is in part because salts that are stressful (commonly and mistakenly referred to as ‘toxic’) do not readily penetrate or damage a dry cell (Hallsworth, 2021). The oligotrophic and/or acidic conditions that occur within the Martian regolith are not biocidal to many types of Earth microbes, most especially those cells that are desiccated and at subzero temperatures (Hallsworth, 2021; 2022).

**Supplementary Text: A note on planetary protection**

The astrobiology scientific community has also been concerned with keeping potential life that originated elsewhere from contaminating Earth (i.e., preventing ‘back contamination’). Although life might have been transported naturally to Earth by meteorites, these back planetary protection concerns focus on samples, such as those from Mars and asteroids, that have been sampled by humans and will be returned to Earth. A National Research Council (National Academy of Sciences, U.S.A.) was charged with an initial thought experiment concerning back contamination as part of Mars Sample Return (National Research Council, 1997). Some years later, a second National Research Council was asked to update the 1997 report, using recent data from Mars and newer hypotheses about Mars’ potentials for habitability and preservation of biosignatures (Farmer *et al*., 2009). How should NASA prepare in case life exists in any extraterrestrial samples brought to Earth by humans? To address this question, both councils had to consider: past and present habitability of Mars; the potential for life on Mars and its likelihood of preservation in Martian rocks and sediments; approaches to biohazard testing; and possible contamination by Martian life on Earth. Due to the nature of the scientific question and limited data from Mars, this work was hypothetical. Results of this grand thought experiment included the recommendations that return samples from Mars be transported in sealed canisters (Kminek *et al*., 2014) and that a specialised sample-receiving facility must be built (Farmer *et al*., 2009). These recommendations have helped to guide logistical considerations for Mars sample return.

**Supplementary Text: Candidate Phyla Radiation**

Hug *et al*. (2016) elaborated on the existence of a large and phylogenetically diverse radiation of the bacterial tree, the Candidate Phyla Radiation (CPR) (Wrighton *et al*., 2012; Brown *et al*., 2015), consisting of uncultivated members with diminutive cells with poorly understood ecological functions and metabolism. More-recent standardised bacterial genome phylogeny suggested the reclassification of the CPR as a single superphylum, the Patescibacteria (Parks *et al*., 2018). Theory-based studies (using *in-silico* analyses of metagenomics data) have driven progress in understanding the biology of this group. For example, Albertsen *et al*. (2013) and Castelle *et al*. (2017; 2018) revealed that Patescibacteria have reduced genomes (<1 Mbp on average) with a severe deficit in the metabolic capacity for *de-novo* biosynthesis. This suggests adaptations to obligate symbiotic or parasitic lifestyles, making them fully reliant on hosts with more complete biosynthetic pathways (Castelle and Banfield, 2018; Castelle *et al*., 2018). Some studies used a hybrid approach (theory-based bioinformatics + practical experiment) to enable microbial isolation and cultivation (Cross *et al*., 2019) or reveal metabolic interactions and novel kinds of symbioses between phylogenetically diverse microbes (La Cono *et al*., 2020).

The Cross *et al*. (2019) study by Mircea Podar (University of Tennessee, Knoxville, TN, USA) and co-workers focused on Saccharibacteria (a candidate phylum within the bacterial Patescibacteria lineage), mainly mouth-dwelling bacteria of which nearly a dozen species live in humans. They make up less than 1% of the mouth's microbiome and are difficult to isolate and grow. Podar's team used a hybrid approach (theory-based approaches + antibody-based experiment) to capture specific bacteria from complex communities as pure cultures using genome-informed antibody engineering via a process coined *reverse genomics-enabled microbiology* (in ‘Targeted isolation and cultivation of uncultivated bacteria by reverse genomics’ by Cross *et al*., 2019). This process includes the following steps: (i) *in-silico* identification of membrane protein-encoding genes in curated metagenome-assembled (MAGs) and single-cell amplified genomes (SAGs), (ii) selection of predicted exposed epitopes, (iii) antibody production, purification, and labeling, and (iv) staining and isolation of target cells from microbiome samples followed by genomic sequencing or cultivation. This approach facilitated the isolation/cultivation of three species-level lineages of Saccharibacteria, all predatory epibionts of diverse Actinobacteria. Moreover, it seems that reverse genomics-enabled cultivation of microorganisms can be applied to any species from as-yet-uncultured branches of the microbial tree of life, obtained from any environment, since using this combination of theory-based approaches (*in-silico* prediction of the three-dimensional structure of membrane-bound proteins) and an antibody-based strategy^[[1]](#footnote-1)^. The study has contributed to the reorganisation of the logical geography of candidate bacterial phylum taxonomy (Figure 2).

An additional outcome of Hug *et al*. (2016) article was stimulating research interest to study the archaeal DPANN superphylum (an acronym, proposed in 2013, and formed by the initials of the first five archaeal candidate phyla discovered, [Diapherotrites](https://en.wikipedia.org/wiki/Diapherotrites), [Parvarchaeota](https://en.wikipedia.org/wiki/Parvarchaeota), [Aenigmarchaeota](https://en.wikipedia.org/wiki/Aenigmarchaeota), [Nanoarchaeota](https://en.wikipedia.org/wiki/Nanoarchaeota) and [Nanohaloarchaeota](https://en.wikipedia.org/wiki/Nanohaloarchaeota)), another large radiation of enigmatic microbes that (like CPR) are widely distributed across Earth’s environments. Metagenome-inferred initial assumptions have indicated that they are consistently distinct from essentially all other *Archaea* owing to their small cells, reduced genomes, limited metabolic capacities, and often episymbiotic associations with other prokaryotic organisms^[[2]](#footnote-2)^ (Dombrowski *et al*., 2019). A study led by M.M.Y. ‘Symbiosis between nanohaloarchaeon and haloarchaeon is based on utilization of different polysaccharides’ combined theory-based approaches with practical experimentation to reveal new insights into microbial metabolism and ecology for a DPANN–host association (La Cono *et al.*, 2020).

*In-silico* analyses of *Ca*. Nanohalobium constans genome data (nanohaloarchaeal ecosymbiont) by La Cono and colleagues led them to realise that this tiny microbe has a full metabolic repertoire allowing it to degrade alpha-glucans (such as starch or glycogen). This metabolic capacity is missing in the genome of the host haloarchaea, *Halomicrobium* sp. LC1Hm (La Cono *et al*., 2020). The La Cono *et al*. study has reorganised our thinking about the ecology of these DPANN *Archaea* (Figure 2). Its findings indicated that the nanohaloarchaeon–haloarchaeon association is a mutualistic symbiosis where each microbe relies on its partner’s ability to degrade different polysaccharides. Given that hypersaline ecosystems often undergo seasonal (and other) changes in the spectrum of available carbon substrates, the receptivity of *Halomicrobium* to Ca. Nanohalobium can be interpreted as a strategy to maximise long-term host fitness. Moreover, this symbiosis might be a kind of survival strategy where there is negligible impact on the host’s fitness during the abundance of chitin but can increase the host’s fitness under other conditions, such as the abundance of alpha-glucans (starch and glycogen). Whereas studies such as those of Cross *et al*. (2019) and La Cono *et al*. (2020) involved practical work, their design and success was based in equal measured on theoretical analyses.

**Supplementary Text: Quantification of competitive interactions**

An unplanned (accidental) study by the J.E.H. research group produced a new methodology to analyse interactions between competing microbes based on the colony geometry of microbial co-cultures over time: ‘A simple inhibition coefficient for quantifying potency of biocontrol agents against plant-pathogenic fungi’ (Cray *et al*., 2015b). The study was accidental in as much as it was neither planned nor foreseen. PhD student Jonathan A. Cray (Queen’s University Belfast) was carrying out culture-based laboratory experiments to elucidate the effects of environmentally pertinent stress parameters on interactions between the plant-pathogenic ascomycete *Fusarium coeruleum* and microbes isolated from the field (potential biocontrol agents)*,* that was published later as a separate study (Cray *et al*., 2016). Early one morning, J.E.H. called into the laboratory to great J.A.C. and during their conversation, J.A.C. mentioned that the results of his assay (where a plant pathogen and a potential biocontrol agent had been inoculated onto the same nutrient medium in Petri plates) were somewhat “messy”. This was because (for example) in some cases colonies had grown over each other, in other cases one microbe had not grown at all (possibly due to inhibition by diffusion of antimicrobials through the agar and/or diffusion of volatile organic compounds through the air space), and in other cases a microbe has quickly swamped the agar surface because it was motile. J.A.C. lamented the apparent lack of clarity within the experimental dataset. At the same time, however, these ‘messy’ data presented an opportunity for an (essentially theory-based) analyses of geometrical and temporal interactions.

This opportunity arose because studies of microbial interactions (often called in the biocontrol literature ‘antagonism assays’) have often used zone-of-inhibition as a measure of competitive ability. The data of J.A.C. clearly showed that inhibition is frequently not related to any zone-of-inhibition because, for example, a microbe could be completely inhibited even when there is no clear zone, and conversely there may be a clear zone but no growth-rate inhibition. Indeed, in general the various types of interaction can be complex and dynamic (even more so when a microbe is motile). Furthermore, previous literature that had characterised the various types of microbial interaction on a solid surface had identified only a subset of those exhibited in the agar cultures within J.A.C.’s several hundred Petri plates (for references, see Cray *et al*., 2015b). Therefore, J.E.H. suggested making a geometry-based analysis of colony development in the competing co-cultures, identifying the key measures of competitive outcomes and then deriving an equation to integrate and quantify these in the form of an inhibition coefficient. The resulting article was submitted to the journal *Biological Control* and, upon review, accepted without any revisions, and, since publication, both J.E.H.’s research group (Cray *et al*., 2016) and other research groups have begun to use the inhibition coefficient in their practical research studies (e.g., Hirpara *et al*., 2017; 2019; Preto *et al*., 2017; Amna *et al*., 2020; Mehmood *et al*., 2021).

**Supplementary Text: “The Child’s Vision of the World”**

Quotation from the chapter ‘The Child’s Vision of the World’ of *The Hidden Order of Art* (Ehrenzweig, 1970):

“The study of…the scanning process in science [and of art’s unconscious substructure] offers the needed opportunity for observing the creative techniques of the ego and the way in which it makes use of the dispersed structure of unconscious perception. The chaos of the unconscious is as deceptive as the chaos of outer reality. In either case we need the less differentiated techniques of unconscious vision to become aware of their hidden order [in other words, conscious thought tends to artificially fragment objective reality]. The scientist has to face [this] fragmentation of physical reality with courage. [He/she] has to scan a multitude of possible links that could make sense out of apparent chaos…[he/she] needs the more dispersed (undifferentiated) structure of low-level vision in order to project the missing order into reality. At the same time – and this is the immense psychological gain – [he/she] will make a constructive use of his unconscious faculties and achieve the integration of his own ego. In creativity, outer and inner reality will always be organized together by the same indivisible process^[[3]](#footnote-3)^…unconscious scanning makes use of undifferentiated modes of vision that to normal awareness would seem chaotic. Hence comes the impression that the primary process merely produces chaotic fantasy material that has to be ordered and shaped by the ego’s secondary process. On the contrary, the primary process is a precision instrument for creative scanning that is far superior to discursive reason and logic.”

*The Hidden Order of Art* (Ehrenzweig, 1970) contents:

Preface
BOOK ONE: CONTROLLING THE WORLD

Part I: Order in Chaos

The Child's Vision of the World

The Two Kinds of Attention

Unconscious Scanning

Part II: Creative Conflict

The Fertile Motif and the Happy Accident

The Fragmentation of 'Modern Art'

The Inner Fabric

Part III: Teaching Creativity

The Three Phases of Creativity

Enveloping Pictorial Space

Abstraction
 Training Spontaneity through the Intellect

BOOK TWO: STIRRING THE IMAGINATION

Part IV: The Theme of the Dying God

The Minimum Content of Art

The Self-Creating God

The Scattered and Buried God

The Devoured and Burned God

Part V: Theoretical Conclusions

Towards a Revision of Current Theory

Ego Dissociation

Appendix: Glossary

References
Index

**Supplementary Text: “Training Spontaneity Through the Intellect”**

Quotation from the chapter ‘Training Spontaneity Through the Intellect’ of *The Hidden Order of Art* (Ehrenzweig, 1970):

“The old cult of free self-expression still lingers on [in art schools]; but it has thoroughly exhausted itself as a stimulus for the student’s imagination. Once upon a time the slogan of free self-expression came as a liberation…[chaffing] against externally imposed conventions and restrictions. The individual pitted himself against society…By one of the many ironical turn-abouts in modern art…today, free self-expression has become a social duty forcibly imposed on the student by teachers, parents and the public alike…[Those involved in the avant-garde art movement of the early 1900s] refused to be caught in any final statement or style knowing well enough that the formalization of their maxims would destroy their meaning. And so it has indeed come to pass. Individual self-expression has turned into another social convention. If we were to formulate a new maxim that today could replace the platitude of free self-expression, it would be the opposite demand. Instead of straining too hard to discover his inner self, the student [as occurs in science] should objectively study the outside world. Because objective factors are alien to the inner self they are better able to act as extraneous ‘accidents’ and so cut across preconceived and defensive clichés. In this way they will be able to tap hidden parts of the personality which have become alienated from the conscious personality. Cool ‘alienation’, then, has to fulfil the function which hot self-expression once filled (Ehrenzweig, 1960)…Somehow – and this is the paradox – our involvement with outer events [including scientific observations/data] is far better able to express our real preoccupations [enabling creative and novel insight] than a direct attempt at looking inside ourselves or into the minds of other people.”.

**Supplementary Text: Early developments in artificial intelligence**

Since the classic paper of Turing (1950) that was described in the main text, hypothesis formulation and automated discovery have been a focus within artificial-intelligence research since the 1960s (Kitano, 2021), in which the artificial intelligence computer software expert system DENDRAL (Lederberg, 1990; Lindsay *et al*., 1993) and later, META-DENDRAL (Buchanan and Feigenbaum, 1978) were engineered to help organic chemists to identify the molecular structure of unknown organic molecules using mass spectrometry data. DENDRAL was conceived by the molecular biologist Joshua Lederberg (Nobel Prize in Physiology or Medicine in 1958) to perform a repetitive and time-consuming task performed by chemists at the time, which consisted of examining a large number of molecular structures to find those that matched the mass spectrometry data. DENDRAL systems performed this task more quickly and with an accuracy equivalent to that of an expert spectrometer. Another system that followed, was the artificial-intelligence medical-expert systems MYCIN developed at Stanford in the 1970s, used to identify bacteria causing severe infections and to recommend antibiotics and their dosage (Shortliffe, 1974). To assess the accuracy of the MYCIN system, a panel of independent experts was used to evaluate the choice of antimicrobials of the MYCIN system and of nine other prescribers. MYCIN received the highest acceptability rate from the panel (65%), compared to the range of acceptability rates received by the other nine specialists (42.5% to 62.5%) (Yu *et al*., 1979). However, MYCIN was never used in routine medical practice, due to some ethical and legal issues raised regarding the use of computers in medicine and also to the computational limitations of availability and infrastructure at the time. Although these are early artificial-intelligence systems that worked with limited data, they helped pave the way for the development of more-sophisticated neural network-based artificial-intelligence systems that are used nowadays to assist researchers in, for example, the discovery of new antibiotics using large gut metagenomics datasets and natural language processing models to find candidate peptides with antimicrobial properties (Ma *et al*., 2022).

**Supplementary Text: Attitudes of journals and funding bodies**

A small number of scientific journals in the area of biology, such as *Journal of Theoretical Biology*, publish only theoretical studies whereas a greater number of journals focus on reports of practical experiments. However, many journals do accommodate scientific developments made via diverse approaches—whether these are theory-based, practical experiments, or hybrid in nature—by offering numerous types of article formats. For example, *Environmental Microbiology* and *Environmental Microbiology* *Reports* feature the following formats: Burning Questions, Correspondence articles, Crystal Ball articles, Editorials, Genomic Update features, ‘How we did it!’ papers, Minireviews, Opinions, Research Articles (either Full Papers or Brief Reports, that may or may not be based on practical experiments), Special Issue articles, etc. Furthermore, *Environmental Microbiology* promotes flexibility and favours innovative approaches within their reviews, as stated in the Author Guidelines: “[Minireviews] bring to the attention of our readership exciting new developments and/or concepts in a timely fashion. They are selective in scope, focused and concise, rather than being comprehensive or historical, and may be somewhat speculative, if this is likely to provoke interesting discussions and stimulate new lines of creative experimentation...” (accessed 18 August 2022). In some academic fields, there is even a differentiation between quantitative and qualitative literature reviews (Green and Hall, 1984; Seers, 2015).

Some multidisciplinary journals also offer a considerable range of article formats. For example, the *Proceedings of the National Academy of Sciences of the United States of America* (*PNAS*) publishes Colloquium Papers, Commentaries, Front Matter, Inaugural Articles, Letters to the Editor, Opinions, Perspectives, and research articles (Research Reports or Brief Reports). Similarly, the journal *Nature* takes articles of various formats: Analysis, Articles, Books and Arts, Careers, Commentary and Worldview, Correspondence, Futures, Hypothesis, Matters Arising, News & Views, News and Commentary, Outlooks, Perspectives, reviews (Reviews and Perspectives), and Technology Features. Some journals, including *Environmental Microbiology*, *Geology*, *ISME J*, *Microbial Biotechnology*, *Nature*, *Nature Climate Change*, and *PNAS* publish some papers that are interdisciplinary or transdisciplinary (e.g., the current article; Steffen, 2009; Weart, 2013; Serrao-Neumann *et al*., 2021; Spencer, 2022).

Nevertheless, we are in favour of a more-flexible approach to publication formats (e.g., opinion-type articles containing a Methods section) that may promote scientific progress by removing constraints and preconceptions on what research approach(es) can be used. Considering the increasing complexity and specialisation of most scientific fields, the ideal of truly inter- or transdisciplinary approaches is potentially valuable and productive. In reality, however, there can be major difficulties with their application. One difficulty is obtaining grants for this type of research due to the difficulty in its evaluation, and another is that few journals focus on publishing inter-/transdisciplinary research, and those that do so tend to specialise in particular topics. Such journals include *Astrobiology*, *iScience*, *Journal of The Royal Society Interface*, and *Science of the Total Environment*. One example of grant funding in the spirit of interdisciplinary research is that provided by the NASA Astrobiology Program (Washington D.C., USA), some of the funding provided by the European Research Executive Agency (e.g., for space-related projects), and that provided by The Wellcome Trust—a UK-based charitable foundation that is focused on funding research to improve human health—for arts projects to promote engagement with artists (and their audiences) with health research topics.

We are in favour of these journals and funding bodies that promote innovative research in this way and believe that, as a research community, we ought to avoid amassing low-risk canonical works that flood the field and compete for the attention of readers, while advancing science very little (if at all) (Chu and Evans, 2021). Having a good balance between theory-based articles proposing new ideas/theories and a larger body of experimental work to support/disprove them, would be a better strategy for the advancement of science than just piling up articles that represent more of the same. We are in favour of creating more opportunities to carry out and publish diverse types of theory-based research studies. For example, paid sabbatical leave and research exchanges for academic faculty members would help to generate the proper time and environment to produce this kind of work. In some cases, employers (or Governments) need to reduce the pressure on researchers to publish as many articles as possible, so that pursuing a high-risk and potentially paradigm-shifting idea would not be crippling from the career standpoint in the event that it ended up being fruitless.

**Supplementary Text: Daniel C. Dennett** **III**

Quotation from Tomás J. Ryan’s introduction (https://www.tcd.ie/biosciences/whatislife/) of Daniel C. Dennett III at the *Schrödinger at 75: The Future of Biology* meeting (September 2018; Trinity College Dublin, Dublin, Ireland); Dennett was a keynote speaker giving a lecture ‘What is Life?’:

“Understanding the nature of information was central to Schrödinger's lectures 75 years ago. Today, we understand the answer to the question of how information is coded in the genome, but we only dance around the question of how information is coded in the brain. What is wrong with this question? It assumes that we are all talking about the same thing. We all know what we mean by the brain but what do we really mean by ‘information’? If we found information in the brain, or in the mind, what would it look like; what would it be made of; how would we read it; how would it evolve? Our understanding of information and its role in life is still both rudimentary and abstract. It is a frontier not just for science but for engineering and for philosophy, and in this broad scope our keynote speaker this evening is author philosopher and cognitive scientist Professor Daniel Dennett.

Dennett is the Austin B. Fletcher Professor of Philosophy at Tufts University in Boston. He originally took his BA at Harvard before doing his DPhil at Oxford University under the mentorship of the late Gilbert Ryle. He has produced seminal philosophical works: on the philosophy of mind, free will, epistemology, and philosophy of religion. His work uniquely incorporates evolutionary biology, cognitive science, and neuroscience; and he does this to create a paradigm of how to understand nature and the origin of consciousness in a non-mystical way.

Dennett has done this not just through academic writing but also through 15 seminal books that are read by scientists and the general public, including *Consciousness Explained* [Dennett, 1991], *Breaking the Spell* [Dennett, 2006], *Darwin's Dangerous Idea* [Dennett, 1995], and *The Intentional Stance* [Dennett, 1987], amongst others. His most recent work, *From Bacteria to Bach and Back: The Evolution of Minds* [Dennett, 2017], he offers a particularly adventurous perspective on the evolution of information, at least information in the broad sense - and how we can think about the future of our own evolution; and while reviewing this book, Mike Gazzaniga, who’s one of our speakers tomorrow, summarised his opinion of it by saying “This is the kind of book you read relish and read again”. I've also read this particular book twice, though I can't honestly say so about Schrödinger's *What is Life?* [Schrödinger, 1944].

There is no ‘Nobel Prize for Philosophy’, but if there was, Professor Dennett would surely have already received it; though he doesn't need to. According to the late [artificial intelligence] researcher Marvin Minsky, “Dennett is our best current philosopher, he is the next Bertrand Russell.” Unlike traditional philosophers, Dan is a student of neuroscience, linguistics, artificial intelligence, and computer science (and psychology). He is redefining the role of a philosopher. Dennett’s approach to philosophy is different to most of his colleagues; he does not engage in theatrical debate; he is not aggressively argumentative or didactic; he doesn't go down narrow logical corridors of analysis; and he doesn't rely on overly artificial but logical discourse. He does not base his views on shaky premises; he starts always with science. His interests are diverse, and he becomes a near-expert in any subfield of science that takes his interest. He learns the facts; he understands the crucial insights; he surveys the boundaries; and familiarises himself with the frontiers of the field and the challenges that lie there. Most crucially, he brings all of this knowledge together and then delivers new syntheses through his landmark works. He marshals the ideas, the questions, the theories into singular stories and worldviews. And he does this through plain English.

Dennett's capacity for synthesis for insightful and interdisciplinary intercultural research puts him in a rare class of thinkers, scientists, and artists such as Sydney Brenner, Lynn Margulis, Ada Lovelace, Jocelyn Bell, James Joyce, Mary Wollstonecraft, David Bowie, and I think, Erwin Schrödinger. The result of this is to introduce the reader to a new way of viewing the world, to see things as they evolved, and to gain a perspective of where we might be going in the future. And beyond the values ought to science, of the scientific utility, it provides us with valuable cultural tools as well.

Dennett is often lambasted by ideologically driven religious groups across the world for his work in promoting atheism, but his work in my opinion offers the most evidence-based but optimistic view of the world and of our own culture. Because it shows most clearly how far we have come from higher primates to hunter-gatherers through nothing but our own resourcefulness despite all our suffering, despite all of the challenges we have brought ourselves here alone without any help, and even if sometimes we allow our more-primitive instincts, that I've always been there, to take over and—because we take our eye off the ball—we are all still here. And we can continue to develop our own evolution and our own culture, which is still in an extremely early stage, if we just keep talking, and—as Dennett reminds us to always do—keep thinking.

Ladies and gentlemen, it is my privilege to introduce the Schrödinger at 75: ‘What is Life’ keynote lecture: please welcome to the stage, Professor Daniel Dennett.”

**Supplementary Text: Global soil health, and a post-human biosphere**

Timely articles that have focused on the importance and urgency of soils as living systems in the context of environmental sustainability and planetary health include: ‘Soil and human security in the 21^st^ century’ (Amundson *et al.* 2021) and ‘The soil crisis: the need to treat as a global health problem and the pivotal role of microbes in prophylaxis and therapy’ (Timmis and Ramos, 2021). They span all levels of soil function, from the biosphere to biogeochemistry, and include the existence of ecosystems and humanity. The Timmis and Ramos article also spans disciplines in as much as it was inspired, in part at least, by medicine. We asked Timmis, whose work spans both environmental and medical microbiology, more about this: “the inspiration was crises, and the nice work on microbiomes which are increasingly shown to regulate health in humans, plants, etc.: it is obvious that it regulates the health of the soil but, most importantly, needs help (like our immune system needs prophylactic and therapeutic help to prevent/overcome some infections and other diseases) to prevent, repair and recover from major abuses, so is directly analogous to clinical interventions in human health. One issue is that problems of soil health, while often viewed as local things, occur all over the globe: we need a global health system for soils; monitoring-epidemiology, prophylaxis, therapy, with new tools and approaches relevant to the problems.” Understanding soil health through the prism of human health has recently been utilised in other microbiome studies (e.g., Banerjee and van der Heijden, 2022).

The article ‘The darkest microbiome—a post-human biosphere’ (Timmis and Hallsworth, 2022) was written at the time that the United Nations Climate Change Conference COP26 was taking place, in Glasgow (Scotland, UK) and, whereas it focuses on Earth, is in essence a thought experiment in astrobiology. It takes the form of a dialogue between two microbes in a biofilm on Earth’s subsurface in the distant future, when the planet’s biosphere persists only underground. These microbes are a *Pseudomonas* (Luca) just 300 years old, and ‘Granny’; a cell more than 780 000 years old. Amongst other issues, the dialogue explores how Earth’s biosphere used to be when the surface was habitable and the evolutionary trajectories of Earth’s lifeforms and biosphere. In doing so, it traverses issues from various microbial metabolisms to the emergence of higher lifeforms, and how and why humans transformed and ultimately degraded the Earth (Timmis and Hallsworth, 2022). The seed for the study, according to K.T., was a recent and enjoyable collaboration with one of us (R.A.) on the deep-subsurface microbiome of the Iberian Pyrite Belt (Puente-Sánchez *et al*., 2018). The Timmis and Hallsworth article was also borne of a concern for the environment, so was dedicated to environmental campaigner Greta T.E.E. Thunberg and naturalist and environmental campaigner Sir David F. Attenborough.

The conversation between two microbes (Timmis and Hallsworth, 2022) provides a historical overview of life on Earth (not least the havoc created by humans), reveals the tenacity of microbial life, and takes the reader into the darkest (subsurface) biosphere of a future over-heated Earth. Its anthropomorphic portrayal of the two microbes accentuates the absence of any human life that is by that time extinct. It may be that when the next major environmental crisis comes, if not before that time, the Timmis and Hallsworth (2022) article—and other concept-based articles on the future biosphere—will acquire some influence among policy-makers (Figure 2). Since the publication of Timmis and Hallsworth (2022), an alarming yet realism-based study of the climate-change end game for humanity ‘Climate Endgame: Exploring catastrophic climate change scenarios’ (Kemp *et al*., 2022) has been published in *PNAS* (August, 2022).

**Supplementary References**

Albertsen, M., Hugenholtz, P., Skarshewski, A., Nielsen, K.L., Tyson, G.W., and Nielsen, P.H. (2013) Genome sequences of rare, uncultured bacteria obtained by differential coverage binning of multiple metagenomes. *Nat Biotechnol* **31**: 533–538.

Alves, F., Stevenson, A., Baxter, E., Gillion, J.L.M., Hejazi, F., Hayes, S., *et al.* (2015) Concomitant osmotic and chaotropicity-induced stresses in *Aspergillus wentii*: compatible solutes determine the biotic window. *Curr Gen* **61**: 457–477.

Amna, Xia, Y., Farooq, M.A., Javed, M.T., Kamran, M.A., Mukhtar, T., *et al*. (2020) Multi-stress tolerant PGPR *Bacillus xiamenensis* PM14 activating sugarcane (*Saccharum officinarum* L.) red rot disease resistance. *Plant Physiol Biochem* **151**: 640–649.

Ames, O. (1939) *Economic Annuals and Human Cultures*. Cambridge: Botanical Museum of Harvard University.

Amundson, R., Berhe, A.A., Hopmans, J.W., Olson, C., Sztein, A.E., and Sparks, D.L. (2015) Soil and human security in the 21st century. *Science* **348**: 1261071.

Anderson, E. (1952) *Plants, Man and Life*. Boston, MA, USA: Little, Brown and Company.

Baker, H.G. (1965) Characteristics and modes of origin of weeds. In *Genetics of Colonizing Species*. Baker, H.G., and Stebbins, G.L. (eds). New York, USA: Academic Press, pp. 147–168.

Ball, P., and Hallsworth, J.E. (2015) Water structure and chaotropicity: their uses, abuses and biological implications. *Phys Chem Chem Phys* **17**: 8297–8305.

Banerjee, S., and van der Heijden, M.G.A. (2022) Soil microbiomes and one health. *Nat Rev Microbiol* https://doi.org/10.1038/s41579-022-00779-w

Banerjee, S., Kirkby, C.A., Schmutter, D., Bissett, A., Kirkegaard, J.A. and Richardson, A.E. (2016) Network analysis reveals functional redundancy and keystone taxa amongst bacterial and fungal communities during organic matter decomposition in an arable soil. *Soil Biol Biochem* **97**: 188–198.

Banerjee, S., Schlaeppi, K., and van der Heijden, M.G. (2018) Keystone taxa as drivers of microbiome structure and functioning. *Nat Rev Microbiol* **16**: 567–576.

Banerjee, S., Walder, F., Büchi, L., Held, A.Y., Gattinger, A., Keller, T., *et al.* (2019) Agricultural intensification reduces microbial network complexity and the abundance of keystone taxa in roots. *ISME J* **13**: 1722–1736.

Bell, A.N.W., Magill, E., Hallsworth, J.E., and Timson, D.J. (2013) Effects of alcohols and compatible solutes on the activity of β-galactosidase. *Appl Biochem Biotechnol* **169**: 786–796.

Benison, K.C. (2006) A Martian Analog in Kansas: Comparing Martian strata with Permian acid saline lake deposits: *Geology* **34**, 385–388.

Benison, K.C. (2019) How to search for life in Martian chemical sediments and their fluid and solid inclusions using petrographic and spectroscopic methods. *Frontiers Environ Sci* **7**: 108.

Benison, K.C. and Bowen, B.B. (2006) Acid saline lake systems give clues about past environments and the search for life on Mars. *Icarus* **183**: 225–229.

Benison, K.C., and LaClair, D.A. (2003) Modern and ancient extremely acid saline deposits: terrestrial analogs for Martian environments? *Astrobiology* **3**: 609–618.

Benison, K.C., Bowen, B.B., Oboh-Ikuenobe, F.E., Jagniecki, E.A., LaClair, D.A., Story, S.L., *et al*. (2007) Sedimentology of acid saline lakes in southern Western Australia: newly described processes and products of an extreme environment. *J Sediment Res* **77**: 366–388.

Benison, K.C., LaClair, D.A., and Walker, J.W. (2008) Acid waters as physical sedimentology agents: Implications for Mars. *Earth & Planet Sci Lett* **270**: 330–337.

Berry, D., and Widder, S. (2014) Deciphering microbial interactions and detecting keystone species with co-occurrence networks. *Front Microbiol* **5**: 219.

Bhaganna, P., Volkers, R.J.M., Bell, A.N.W., Kluge, K., Timson, D.J., McGrath, J.W., *et al*. (2010) Hydrophobic substances induce water stress in microbial cells. *Microbial Biotechnol* **3**: 701–716.

Bhaganna, P., Bielecka, A., Molinari, G., and Hallsworth, J.E. (2016) Protective role of glycerol against benzene stress: insights from the *Pseudomonas putida* proteome. *Curr Genet* **62**: 419–429.

Block, N. (1981) Psychologism and behaviorism. *Philos Rev* **90**: 5–43.

Braga, G.Ú.L. Silva-Junior, G.A., Brancini, G.T.P., Hallsworth, J.E., and Wainwright (2022) Photoantimicrobials in agriculture. *J Photochem Photobiol B: Biol* **235**: 112548.

Brown, A.D. (1990) *Microbial Water Stress Physiology: Principles and Perspectives.* Chichester, UK: Wiley.

Brown, C.T., Hug, L.A., Thomas, B.C., Sharon, I., Castelle, C.J., Singh, A., *et al.* (2015) Unusual biology across a group comprising more than 15% of domain Bacteria. *Nature* **523**: 208–211.

Buchanan, B.G., and Feigenbaum, E.A. (1978) DENDRAL and Meta-DENDRAL: Their applications dimension. *Artif Intell* **11**: 5–24.

Burns, R.G. (1987) Gossans on Mars: spectral features attributed to jarosite. *LPSC* **XVIII**: 141–142.

Burns, R.G. (1994) Schwertmannite on Mars; deposition of this ferric oxyhydroxysulfate mineral in acidic saline meltwaters. *LPSC* **XXV**: 203–204.

Castelle, C.J., and Banfield, J.F. (2018) Major new microbial groups expand diversity and alter our understanding of the tree of life. *Cell* **172**: 1181–1197.

Castelle, C.J., Brown, C.T., Thomas, B.C., Williams, K.H., and Banfield, J.F. (2017) Unusual respiratory capacity and nitrogen metabolism in a Parcubacterium (OD1) of the Candidate Phyla Radiation. *Sci Rep* **7**: 40101.

Castelle, C.J., Brown, C.T., Anantharaman, K., Probst, A.J., Huang, R.H., and Banfield, J.F. (2018) Biosynthetic capacity, metabolic variety and unusual biology in the CPR and DPANN radiations. *Nat Rev Microbiol* **16**: 629–645.

Chin, J.P., Megaw, J., Magill, C.L., Nowotarski, K., Williams, J.P., Bhaganna, P., *et al*. (2010) Solutes determine the temperature windows for microbial survival and growth. *Proc Natl Acad Sci USA* **107**: 7835–7840.

Chu, J.S., and Evans, J.A. (2021) Slowed canonical progress in large fields of science. *Proc Natl Acad Sci USA* **118**: e2021636118.

Clark, B.C. (1979) Chemical and physical microenvironments at the Viking landing sites. *J Mol Evol* **14**: 13–31.

Clark, B.C. (1994) Acid waters as agents of change on a cold early Mars. *LPSC* **XXV**: 263–264.

Cross, K.L., Campbell, J.H., Balachandran, M., Campbell, A.G., Cooper, C.J., Griffin, A., *et al.* (2019) Targeted isolation and cultivation of uncultivated bacteria by reverse genomics. *Nat Biotechnol* **37**: 1314–1321.

Cray, J.A., Bell, A.N.W., Bhaganna, P., Mswaka, A.Y., Timson, D.J., and Hallsworth, J.E. (2013a) The biology of habitat dominance; can microbes behave as weeds? *Microbial Biotechnol* **6**: 453–492.

Cray, J.A., Russell, J.T., Timson, D.J., Singhal, R.S., and Hallsworth, J.E. (2013b) A universal measure of chaotropicity and kosmotropicity. *Environ Microbiol* **15**: 287–296.

Cray, J.A., Stevenson, A., Ball, P., Bankar, S.B., Eleutherio, E.C.A., Ezeji, T.C., *et al*. (2015a) Chaotropicity: a key factor in product tolerance of biofuel-producing microorganisms. *Curr Opin Biotechnol* **33**: 258–259.

Cray, J.A., Houghton, J.D., Cooke, L.R., and Hallsworth, J.E. (2015b) A simple inhibition coefficient for quantifying potency of biocontrol agents against plant-pathogenic fungi. *Biol Control* **81**: 93–100.

Cray, J.A., Connor, M.C., Stevenson, A., Houghton, J.D.R., Rangel, D.E.N., Cooke, L.R., *et al*. (2016) Biocontrol agents promote growth of potato pathogens, depending on environmental conditions. *Microbial Biotechnol* **9**: 330–354.

Curtis, T.P., Sloan, W.T., and Scannell, J.W. (2002) Estimating prokaryotic diversity and its limits. *Proc Natl Acad Sci USA* **99**: 10494–10499.

Dennett, D.C. (1987). The Intentional Stance. Cambridge, MA: The MIT Press.

Dennett, D.C. (1991) *Consciousness Explained*. Boston, MA: Little, Brown and Co.

Dennett, D.C. (1995) *Darwin's Dangerous Idea: Evolution and the Meanings of Life*. New York: Simon & Schuster.

Dennett, D.C. (2006) Breaking the Spell*: Religion as a Natural Phenomenon.* New York: Viking.

Dennett, D.C. (2017) *From Bacteria to Bach and Back: The Evolution of Minds.* New York: W.W. Norton & Company.

Dombrowski, N., Lee, J.H., Williams, T.A., Offre, P., and Spang, A. (2019) Genomic diversity, lifestyles and evolutionary origins of DPANN archaea. *FEMS Microbiol Lett* **366**: fnz008.

Dykhuizen, D.E. (1998) Santa Rosalia revisited: why are there so many species of bacteria? *Antonie Van Leeuwenhoek* **73**: 25–33.

Ehlmann, B.L., Swayze, G.A., Milliken, R.E., Wray, J.J., Mustard, J.F., Breit, G.N., *et al.* (2016) Discovery of alunite in Cross Crater, Terra Sirenum, Mars: Evidence for precipitation from acidic, sulfurous groundwaters on the margin of a paleolake. *Am Min* **101**: 1527–1542.

Ehrenzweig, A. (1960) Alienation versus self-expression *The Listener* **LXIII**: 1613.

Ehrenzweig, A. (1970) *The Hidden Order of Art: A Study in the Psychology of Artistic Imagination.* London: Palladin.

Farmer, J.D., Bell, J.F., III, Benison, K.C., Boynton, W.V., Cady, S.L., Ferris, F.G., *et al*. (2009) *Assessment of Planetary Protection Requirements for Mars Sample Return Missions/*Space Studies Board, National Research Council, National Academy Press: Washington, D.C.

Fox-Powell, M.G., Hallsworth, J.E., Cousins, C.R., and Cockell, C.S. (2016) Ionic strength is a barrier to the habitability of Mars. *Astrobiology* **16**: 427–442.

Gardner, H.E. (1983) *Frames of Mind: The Theory of Multiple Intelligences.* New York: Basic Books.

Giovannoni, S.J. Britschgi, T.B., Moyer, C.L., and Field, K.G. (1990) Genetic diversity in Sargasso Sea bacterioplankton. *Nature* **345**: 60–63.

Green, B.F., and Hall, J.A. (1984) Quantitative methods for literature reviews. *Annu Rev Psychol* **35**: 37–53.

Hagström, Å., Pommier, T., Rohwer, F., Simu, K., Stolte, W., Svensson, D., and Zweifel, U.L. (2002) Use of 16S ribosomal DNA for delineation of marine bacterioplankton species. *Appl Environ Microbiol* **68**: 3628–3633.

Hajishengallis, G., Liang, S., Payne, M.A., Hashim, A., Jotwani, R., Eskan, M.A., *et al*. (2011) Low-abundance biofilm species orchestrates inflammatory periodontal disease through the commensal microbiota and complement. *Cell Host Microbe* **10**: 497–506.

Hallsworth, J.E. (1998) Ethanol-induced water stress in yeast. *J Ferment Bioeng* **85**: 125–137.

Hallsworth, J.E. (2018) Stress-free microbes lack vitality. *Fungal Biol* **122**: 379–385.

Hallsworth, J.E. (2019) Microbial unknowns at the saline limits for life. *Nat Ecol Evol* **3**: 1503–1504.

Hallsworth, J.E. (2020) Salt deliquescence can support extraterrestrial life*. Nat Astron* **4**: 739–740.

Hallsworth, J.E. (2021) Mars’ surface is not universally biocidal. *Environ Microbiol* **23**: 3345–3350.

Hallsworth, J.E. (2022) Water is a preservative of microbes. *Microbial Biotechnol* **15**: 191–214.

Hallsworth, J.E., Heim, S., and Timmis, K.N. (2003a) Chaotropic solutes cause water stress in *Pseudomonas putida*. *Environ Microbiol* **5**: 1270–1280.

Hallsworth, J.E., Prior, B.A., Nomura, Y., Iwahara, M., and Timmis, K.N. (2003b) Compatible solutes protect against chaotrope (ethanol)-induced, nonosmotic water stress. *Appl Environ Microbiol* **69**: 7032–7034.

Hallsworth, J.E., Yakimov, M.M., Golyshin, P.N., Gillion, J.L.M., D’Auria, G., Alves, F.L., *et al*. (2007) Limits of life in MgCl_2_-containing environments: chaotropicity defines the window. *Environ Microbiol* **9**: 803–813.

Hamill, P.G., Stevenson, A., McMullan, P.E., Williams, J.P., Lewis, A.D.R., S., Sudharsan, *et al*. (2020) Microbial lag phase can be indicative of, or independent from, cellular stress. *Sci Rep* **10**: 5948

Hamm, J.N., Erdmann, S., Eloe-Fadrosh, E.A., Angeloni, A., Zhong, L., Brownlee, C., *et al.* (2019) Unexpected host dependency of Antarctic Nanohaloarchaeota. *Proc Natl Acad Sci USA* **116**: 14661–14670.

Hill, T.A. (1977) The Biology of Weeds. London, UK: E. Arnold.

Hirpara, D.G., Gajera, H.P., Hirapara, J.G., Golakiya, B.A. (2017) Inhibition coefficient and molecular diversity of multi stress tolerant *Trichoderma* as potential biocontrol agent against *Sclerotium rolfsii* Sacc. *Infect Genet Evol* **55**: 75–92.

Hirpara, D.G., Gajera, H.P., Patel, A.K., Katakpara, Z.A., and Golakiya, B.A. (2019) Molecular insights into development of *Trichoderma interfusants* for multistress tolerance enhancing antagonism against *Sclerotium rolfsii* Sacc. *J Cell Physiol* **234**: 7368–7383.

Hug, L.A., Baker, B.J., Anantharaman, K., Brown, C.T., Probst, A.J., Castelle, C.J., *et al.* (2016) A new view of the tree of life. *Nat Microbiol* **1**: 16048.

Imlay, J.A. (2019) Where in the world do bacteria experience oxidative stress? *Environ Microbiol* **21**: 521–530.

Kemp, L., Xu, C., Depledge, J., Ebi, K.L., Gibbins, G., Kohler, T.A., *et al*. (2022) Climate Endgame: Exploring catastrophic climate change scenarios. *Proc Natl Acad Sci USA* **119**: e2108146119.

Kitano, H. (2021) Nobel Turing Challenge: creating the engine for scientific discovery. *npj Syst Biol Appl* **7**: 29.

Kminek, G., Rummel, J.D., Cockell, C.S., Atlas, R., Barlow, N., Beaty, D., *et al*. (2010) Report of the COSPAR Mars special regions colloquium. *Adv Space Res* **46**: 811–829.

Kminek, G., Conley, C., Allen, C.C., Bartlett, D.H., Beaty, D. W., Benning, L.G., *et al*. (2014) Report of the workshop for life detection in samples from Mars. *Life Sci Space Res* **2**: 1–5.

Koski, M.H., Meindl, G.A., Arceo-Gómez, G., Wolowshi, M., LeCroy, K.A., and Asman, T.-L. (2015) Plant–flower visitor networks in a serpentine metacommunity: assessing traits associated with keystone plant species. *Arthropod Plant Interact* **9**: 9–21.

Kumpitsch, C., Fischmeister, F.Ph.S., Mahnert, A. Lackner, S., Wilding, M., Sturm, C., *et al.* (2021) Reduced B12 uptake and increased gastrointestinal formate are associated with archaeome-mediated breath methane emission in humans. *Microbiome* **9**: 193.

Kuntz, T.M., and Gilbert, J.A. (2017) Introducing the microbiome into precision medicine. *Trends Pharmacol Sci* **38**: 81–91.

La Cono, V., Messina, E., Rohde, M., Arcadi, E., Ciordia, S., Crisafi, F., *et al*. (2020) Symbiosis between nanohaloarchaeon and haloarchaeon is based on utilization of different polysaccharides. *Proc Natl Acad Sci USA* **117**: 20223–20234.

Lederberg, J. (1990) How DENDRAL was conceived and born. *A History of Medical Informatics.* Blum, B.I., and Duncan, K. (Eds). New York: Association for Computing Machinery Press. pp. 14–44.

Lee, C.J.D., McMullan, P.E., O’Kane, C.J., Stevenson, A., Santos, I.C., Roy, C., *et al*. (2018) NaCl-saturated brines are thermodynamically moderate, rather than extreme, microbial habitats. *FEMS Microbiol Rev* **42**: 672–693.

Lindsay, R., Buchanan, B., Feigenbaum, E., and Lederberg, J. (1993) DENDRAL: a case study of the first expert system for scientific hypothesis formation. *Artif Intell* **61**: 209–261.

Liu, L., Zhang, H., He, J., and Dong S. (2020) Investigation on the stress response of microbes in acute toxicity assay. *Anal Chim Acta* **1099**: 46–51.

Long, H.C. (1932) *Weeds of Grass Land*. London, UK: H.M.S.O.

Ma, Y., Guo, Z., Xia, B., Zhang, Y., Liu, X., Yu, Y., *et al*. (2022) Identification of antimicrobial peptides from the human gut microbiome using deep learning. *Nat Biotechnol* **40**: 921–931.

McCammick, E.M., Gomase, V.S., Timson, D.J., McGenity, T.J., and Hallsworth, J.E. (2010) Water-hydrophobic compound interactions with the microbial cell. In *Handbook of Hydrocarbon and Lipid Microbiology – Hydrocarbons, Oils and Lipids: Diversity, Properties and Formation*, vol 2, Timmis, K.N. (ed). New York, NY, USA: Springer, pp. 1451–1466.

Mehmood, S., Muneer, M.A., Tahir, M. Javed, M.T., Mahmood, T., Afridi, M.S., *et al.* (2021) Deciphering distinct biological control and growth promoting potential of multi-stress tolerant *Bacillus subtilis* PM32 for potato stem canker. *Physiol Mol Biol Plants* **27**: 2101–2114.

Meindl, R.S., Chaney, M.E., and Lovejoy, C.O. (2018) Early hominids may have been weed species. *Proc Natl Acad Sci USA* **115**: 1244–1249.

Mormile, M.R., Hong, B.Y., and Benison, K.C. (2009) Molecular analysis of the microbial communities of Mars-analog lakes in Western Australia. *Astrobiology* **9**: 919–930.

Mota, M.N., Martins, L.C., and Sá-Correia, I. (2021) The identification of genetic determinants of methanol tolerance in yeast suggests differences in methanol and ethanol toxicity mechanisms and candidates for improved methanol tolerance engineering. J Fungi **7**: 90.

National Research Council (1997) *Mars Sample Return: Issues and Recommendations.* Space Studies Board, National Research Council, The National Academies Press; Washington, D.C.

Noel, D., Gelhaye, E., Darnet, S., Hallsworth, J. E., Sormani, R., and Morel-Rouhier, M. (2023) Modes-of-action of antifungal compounds: stressors and (target-site-specific) toxins, toxicants, or *toxin*–*stressors. Microbial Biotechnol* In press.

Oren, A., and Hallsworth, J.E. (2014) Microbial weeds in hypersaline habitats: the enigma of the weed-like *Haloferax mediterranei*. *FEMS Microbiol Lett* **359**: 134–142.

Paine, R.T. (1969) A note on trophic complexity and community stability. *Am Nat* **103**: 91–93.

Parks, D.H., Chuvochina, M., Waite, D.W., Rinke, C., Skarshewski, A., Chaumeil, P.A. and Hugenholtz, P. (2018) A standardized bacterial taxonomy based on genome phylogeny substantially revises the tree of life. *Nat Biotechnol* **36**: 996–1004.

Pedrós-Alió, C. (2006). Marine microbial diversity: can it be determined? *Trends Microbiol* **14**: 257–263.

Pedrós-Alió, C. (2013) Rare biosphere. In *Encyclopedia of Biodiversity*, Levin SA (Ed.), second edition vol. 6: Chapter 407, pp. 345––351. Waltham, MA, USA: Academic Press.

Preto, G., Martins, F., Pereira, J.A., and Baptista, P. (2017) Fungal community in olive fruits of cultivars with different susceptibilities to anthracnose and selection of isolates to be used as biocontrol agents. *Biol Control* **110**: 1–9.

Puente-Sánchez, F., Arce-Rodríguez, A., Oggerin, M., Carcía-Villadangos, M., Moreno-Paz, M., Blanco, Y., *et al.* (2018) Viable cyanobacteria in the deep continental subsurface. *Proc Natl Acad Sci USA* **115**: 10702–10707.

Qin, Y., Havulinna, A.S., Liu, Y., Jousilahti, P., Ritchie, S.C., Tokolyi, A., *et al*. (2022) Combined effects of host genetics and diet on human gut microbiota and incident disease in a single population cohort. *Nat Genet* **54**: 134–142.

Rath, K.M., Maheshwari, A., Bengtson, P., and Rousk, J. (2016) Comparative toxicities of salts on microbial processes in soil. *Appl Environ Microbiol* **82**: 2012–2020.

Rummel, J.D., Beaty, D.W., Jones, M.A., Bakermans, C., Barlow, N.G., Boston, P.J., *et al*. (2014) A new analysis of Mars “special regions”: findings of the second MEPAG Special Regions Science Analysis Group (SR-SAG2). *Astrobiology* **14**: 887–968.

Ryle, G. (1949) The Concept of Mind. London: Hutchinson's University Library.

Sagan, L. (1967) On the origin of mitosing cells. *J Theoret Biol* **14**: 225–274.

Santos, R., Stevenson, A., de Carvalho, C.C.C.R., Grant, I.R., and Hallsworth, J.E. (2015) Extraordinary solute stress tolerance contributes to the environmental tenacity of mycobacteria. *Environ Microbiol Rep* **7**: 746–764.

Schrödinger, E. (1944). *What is Life? The Physical Aspect of the Living Cell.* Cambridge: Cambridge University Press.

Schuerger, A.C., Ulrich, R., Berry, B.J., and Nicholson, W.L. (2013) Growth of *Serratia liquefaciens* under 7 mbar, 0°C, and CO_2_-enriched anoxic atmospheres. *Astrobiology* **13**: 115–131.

Schuerger, A.C., Mickol, R.L., and Schwendner, P. (2020) The hypopiezotolerant bacterium, *Serratia liquefaciens* failed to grow in Mars analog soils under simulated Martian conditions at 7 hPa. *Life* **10**: 77.

Seers, K. (2015) Qualitative systematic reviews: their importance for our understanding of research relevant to pain. *Br J Pain* **9**: 36–40.

Serrao-Neumann, S., de Araújo Moreira, F., Dalla Fontana, M., Torres, R.R., Lapola, D.D., Nunes, L.H., *et al.* (2021) Advancing transdisciplinary adaptation research practice. *Nat Clim Chang* **11**: 1006–1008.

Shortliffe, E.H. (1974) *MYCIN: A Rule-Based Computer Program for Advising Physicians Regarding Antimicrobial Therapy Selection*. Doctoral Dissertation. Report number: STAN-CS-74-465. Stanford University: Stanford.

Spencer, C.J. (2022) Biogeodynamics: Coupled evolution of the biosphere, atmosphere, and lithosphere. *Geology* **50**: 867–868.

Spry, J.A., Siegel, B., Kminek, G., Bakermans, C., Benardini, J.N., Beltran, E., *et al*. (2021) Planetary protection knowledge gaps and enabling science for human Mars missions. *Bull Am Astron Soc* **53**: 205.

Sqyures, S.W., Grotzinger, J.P., Arvidson, R.E., Bell III, J.F., Calvin, W., Christensen, P.R., *et al*. (2004). In situ evidence for an ancient aqueous environment at Meridiani Planum, Mars. *Science* **306**: 1709–1714.

Steffen, W. (2009) Interdisciplinary research for managing ecosystem services*. Proc Natl Acad Sci U S A* **106**: 1301–1302.

Stevenson, A., Cray, J.A., Williams, J.P., Santos, R., Sahay, R., Neuenkirchen, N., *et al*. (2015a) Is there a common water-activity limit for the three domains of life? *ISME J* **9**: 1333–1351.

Stevenson, A., Burkhardt, J., Cockell, C.S., Cray, J.A., Dijksterhuis, J., Fox-Powell, M., *et al.* (2015b) Multiplication of microbes below 0.690 water activity: implications for terrestrial and extraterrestrial life. *Environ Microbiol* **2**: 257– 277.

Stevenson, A., Hamill, P.G., Medina, A., Kminek, G., Rummel, J.D., Dijksterhuis, J., *et al.* (2017a) Glycerol enhances fungal germination at the water-activity limit for life. *Environ Microbiol* **19**: 947–967.

Stevenson, A., Hamill, P.G., O’Kane, C.J., Kminek, G., Rummel, J.D., Voytek, M.A., *et al*. (2017b) *Aspergillus penicillioides* differentiation and cell division at 0.585 water activity. *Environ Microbiol* **19**: 687–697.

Suryawanshi, R.K., Patil, C.D., Koli, S.H., Hallsworth, J E., and Patil, S.V. (2017) Antimicrobial activity of prodigiosin is attributable to plasma-membrane disruption. *Nat Prod Res* **31**: 527–577.

Timmis, K., and Hallsworth, J.E. (2022) The darkest microbiome—a post-human biosphere. *Microbial Biotechnol* **15**: 176–185.

Timmis, K., and Ramos, J.L. (2021) The soil crisis: the need to treat as a global health problem and the pivotal role of microbes in prophylaxis and therapy. *Microb Biotechnol* **14**: 769–797.

Trinci, P.J., and Ryley, J.F. (Eds.) (1984) *Mode of Action of Antifungal Agents*. Cambridge (UK): Cambridge University Press.

Turing, A. (1950) Computing machinery and intelligence. *Mind* **LIX**: 433–460.

Valette, N., Renou, J., Boutilliat, A., Fernández-González, A.J., Gautier, V., Silar, P., *et al.* (2021) OSIP1 is a self-assembling DUF3129 protein required to protect fungal cells from toxins and stressors. *Environ Microbiol* **23**: 1594–1607.

Vick-Majors, T.J., Priscu, J.C., and Amaral-Zettler, L.A. (2014) Modular community structure suggests metabolic plasticity during the transition to polar night in ice-covered Antarctic lakes. *ISME J* **8**: 778–789.

van Wijngaarden, R.P.A., Van Den Brink, P.J., Oude Voshaar, J.H., and Leeuwangh, P. (1995) Ordination techniques for analysing response of biological communities to toxic stress in experimental ecosystems. *Ecotoxicology* **4**: 61–77.

Weart, S. (2013) Rise of interdisciplinary research on climate. *Proc Natl Acad Sci U S A* **110**: Suppl 1 (Suppl 1): 3657–3664.

Williams, J.P., and Hallsworth, J.E. (2009) Limits of life in hostile environments; no limits to biosphere function? *Environ Microbiol* **11**: 3292–3308.

Wrighton, K.C., Thomas, B.C., Sharon, I., Miller, C.S., Castelle, C.J., VerBerkmoes, N.C., *et al*. (2012) Fermentation, hydrogen, and sulfur metabolism in multiple uncultivated bacterial phyla. *Science* **337**: 1661–1665.

Yakimov, M.M., Lo Cono, V., La Spada, G., Bortoluzzi, G., Messina, E., Smedile, F., *et al*. (2015) Microbial community of seawater-brine interface of the deep-sea brine Lake *Kryos* as revealed by recovery of mRNA are active below the chaotropicity limit of life. *Environ Microbiol* **17**: 364–382.

Yu, V.L., Fagan, L.M., Wraith, S.M., Clancey, W.J., Scott, A.C., Hannigan, J., *et al*. (1979) Antimicrobial selection by a computer: A blinded evaluation by infectious diseases experts. *JAMA* **242**: 1279–1282.

Zaikova, E., Benison, K.C., Mormile, M.R., and Johnson, S.S. (2018) Microbial communities and their predicted metabolic functions in a desiccating acid salt lake. *Extremophiles* **22**: 367–379.

Ze, X., Duncan, S.H., Louis, P., and Flint, H.J. (2012) *Ruminococcus bromii* is a keystone species for the degradation of resistant starch in the human colon. *ISME J* **6**: 1535–1543.

1. Podar and colleagues have also isolated and cultivated human previously uncultured Gracilibacteria (candidate bacterial phylum within Patescibacteria lineage) (Cross *et al*., 2019). [↑](#footnote-ref-1)
2. This was confirmed by the cultivation of two different species of the *Candidate* phylum Nanohaloarchaeota (members of DPANN superphylum) in binary culture with corresponding haloarchaeal hosts (Hamm *et al*., 2019; La Cono *et al*., 2020). [↑](#footnote-ref-2)
3. N.B. The authors of the current manuscript do not wish to suggest that the creative process in scientific thinking is confined in any way to science beyond the experiment. [↑](#footnote-ref-3)
